# Non-invasive detection of hazardous materials with a thermal-to-epithermal neutron station: a feasibility study towards practical application

**DOI:** 10.1038/s41598-024-69290-x

**Published:** 2024-08-10

**Authors:** Michał Silarski, Katarzyna Dziedzic-Kocurek, Kacper Drużbicki, Radosław Reterski, Patryk Grabowski, Matthew Krzystyniak

**Affiliations:** 1 M. Smoluchowski Institute of Physics of the Jagiellonian University, Łojasiewicza 11, 30-348 Cracow, Poland; 2 Centre of Molecular and Macromolecular Studies, Polish Academy of Sciences, Sienkiewicza 112, 90-363 Lodz, Poland; 3 Faculty of Chemistry, Jagiellonian University, Gronostajowa 2, 30-387 Cracow, Poland; 4 ISIS Neutron and Muon Source, STFC Rutherford Appleton Laboratory, Oxford, OX11 0QX UK

**Keywords:** Limits of detection and quantitation of hazardous materials, Neutron Compton scattering, Neutron transmission, Total neutron cross section, Natural hazards, Structure of solids and liquids, Sensors

## Abstract

The growing scale of the devastation that even a single terrorist attack can cause requires more effective methods for the detection of hazardous materials. In particular, there are no solutions for effectively monitoring threats at sea, both for the off-shore infrastructure and ports. Currently, state-of-the-art detection methods determine the density distribution and the shapes of tested subjects but only allow for a limited degree of substance identification. This work aims to present a feasibility study of the possible usage of several methods available on the thermal-to-epithermal neutron station, VESUVIO, at the ISIS neutron and muon spallation source, UK, for the detection of hazardous materials. To this end, we present the results of a series of experiments performed concurrently employing neutron transmission and Compton scattering using melamine, a commonly used explosive surrogate, in order to determine its signal characteristics and limits of detection and quantitation. The experiments are supported by first-principles modelling, providing detailed scrutiny of the material structure and the nuclear dynamics behind the neutron scattering observables.

## Introduction

The 21st century appears to be when the dangers of hazardous materials are beginning to take on special significance. The global number of armed conflicts and terrorist threats is increasing, and, as a consequence, the number of unexploded ordinances (UXO) is growing rapidly, inducing serious threats for citizens (e.g. in Ukraine) now and soon. On the other hand, remnants of the old conflicts are also starting to pose severe environmental problems. A good example is the Baltic Sea, a “storage” for hundreds of thousands of tonnes of munition, being a kind of legacy of the two World Wars^1^. Apart from the explosive materials (TNT, RDX), the dump sites contain many chemical agents developed and produced both by the Axis and Allies during World War 2. These are mainly mustard gas, Clark I and II, and Adamsite^2^. This ammunition was thrown overboard, either loose (both bombs and shells) or in containers; some ships were also sunk. Moreover, part of this arsenal was tossed overboard during transport to the Baltic dump sites. Thus, the precise amount of sunk hazardous materials is not known^2^. Many of these shells have corroded and leaked, and as a result, they are releasing their toxic contents into the environment. This poses a threat not only to the flora and fauna of the Baltic Sea but also to the safety of the navy and civilians on the coasts. There are documented cases of fishermen who fished out munitions containing yperite or TNT. These materials have also been washed up by storms on Baltic beaches. Nowadays, this problem is recognised by the governments of the Baltic Sea countries and the European Union^3^.

Due to the limitations of the currently available methods of detecting and identifying hazardous materials, the costs of demining larger post-war areas are very high on the ground and even higher when it comes to the substances remaining at the seabed. The detection and identification of hazardous materials is currently based primarily on using X-rays, radars, and induction detectors. All these methods allow for the detection of metals or determination of the shape of the objects buried underground. In the aquatic environment, for detecting mines and dangerous chemicals, one primarily uses sonars, which allow one to determine only the position and shape of the object, yet without any information on its chemical composition. Therefore, the detection of any suspicious object requires additional verification. Thus, the limitations of commonly used methods and the growing need for mobile and non-invasive devices allowing for effective and rapid recognition of threats lead to the constant search for novel solutions^4^. Several methods are being attempted as alternatives to or in addition to standard detection techniques, including Raman spectroscopy^5^, gravimetry^6^, or high-frequency radars^7^. Particularly promising in terms of fast, remote, and non-destructive detection and recognition of dangerous substances is the use of neutron activation analysis (NAA)^8^. It employs fast neutrons as probes, penetrating the tested object even if it is concealed in a container or buried in the ground. Interacting with the material, they also induce the emission of secondary $\gamma$ radiation in the inelastic scattering or neutron capture processes. The latter occurs when neutrons are thermalised before or after entering the tested substance. Energies of the emitted $\gamma$-rays are characteristic of each isotope in the irradiated object. Thus, their detection allows for the identification of the elemental content of the tested substance. Several implemented devices based on the NAA operate on the ground (see e.g.^9–11^). In practice, however, there is no solution using neutrons to detect hazardous materials in the aquatic environment. So far, only the UNCOSS^12^ project has attempted to construct a prototype device operating underwater. The main issue is connected with the time of inspection needed to quantify whether the scanned object is dangerous. Moreover, a high $\gamma$ radiation background hinders detection, especially in the aquatic environment. These issues are being addressed within the SABAT project^13^ by applying air guides for neutrons and secondary $\gamma$–quanta, anti-Compton shielding, and the use of associated particle imagining (API)^8^. However, the best sensitivity of such sensors is expected to be at the level of grams^4,14^, making them unuseful in some applications (e.g. chemical agent contamination).

In this work, we report the results of a feasibility study employing neutron transmission (NT) and Compton scattering (NCS) for the detection of hazardous materials. To this end, we rely on a series of measurements performed using the VESUVIO thermal-to-epithermal neutron station at the ISIS neutron and muon spallation source near Oxford, UK, on a substance widely accepted as a surrogate of both explosives and drugs with similar elemental content and structure, the melamine ($\hbox {C}_{3}\hbox {H}_{6}\hbox {N}_{6}$)^15–21^.

A concurrent measurement employing NT and NCS can present an interesting complement or alternative to NAA as a potent tool for the detection and quantitation of hazardous and illicit substances for a number of reasons. Firstly, the neutron Compton scattering (NCS) is sensitive to the isotopic composition of an analyte^22,23^. In contrast, neutron transmission (NT), especially when applied to organic samples^24,25^, is very sensitive to both the isotopic content and the functional groups present in the analysed substance. Thus, NT and NCS, when applied concurrently, allow for distinguishing isomers, which presents a significant improvement over the use of NAA alone in hazardous and illicit substance detection.

Secondly, NT and NCS are much less sensitive to $\gamma$ radiation background than NAA. It owes to the fact that both NT and NCS are detected using the time-of-flight (TOF) technique, and the range of TOF values that are relevant for constructing the shapes of NT and NCS spectra is beyond the short TOF range, which is usually affected by an initial $\gamma$ flash produced either by a neutron spallation or a series of nuclear reactions in a compact neutron source. Moreover, the neutron detectors that are presently used at VESUVIO have been optimised over the past few decades towards the best possible signal-to-noise and count rates and low saturation and interference risk from $\gamma$ radiation^22,23,26^.

Finally, the use of the TOF technique allows for the detection of detailed shapes of NT and NCS spectra that can be modelled using *ab initio* materials modelling techniques such as harmonic lattice dynamics and molecular dynamics calculations. The experimental protocol presented here, whereby the NT and NCS experiments are augmented by the *ab initio* materials modelling techniques, exploits an intrinsic synergy between the theoretical and experimental results. On the one hand, NT and NCS techniques can be used as benchmarks for the *ab initio* results. NCS provides a unique set of quantum observables related to nuclear quantum effects (NQEs), the widths of nuclear momentum distributions (NMD widths) that describe the local binding and confinement of isotopic species and the accompanying degree of nuclear quantum delocalisation (NQD) in space. The NT technique provides the total neutron cross-section curves, measured as functions of incident neutron energy. Both types of observables can be compared against their counterparts computed from the atom-projected vibrational densities of states obtained from *ab initio* harmonic lattice dynamics or molecular dynamics simulations. Moreover, whereas the NMD widths are more sensitive to the high-frequency parts of the vibrational densities of states, the shapes of the total neutron cross-section curves are more sensitive to low-to-medium frequencies. Thus, concurrent use of both NT and NCS provides a very stringent, global benchmarking protocol of the *ab initio* simulations, which presents a value by itself. On the other hand, one can use the results of *ab initio* simulations of NT and NCS observables as constraints in the fitting of experimental NT and NCS data and then use such constraint fits to increase the accuracy and sensitivity of the detection protocol of the sought hazardous and illicit substances.

Apart from substance identification by concurrent application of NT and NCS and *ab initio* materials modelling, the experiments described in this work have been designed to determine the limits of detection (LODs) and quantification (LOQs). To this end, the present work extends the experimental protocol previously established for detecting hydrogen in the solid state using VESUVIO^27^. Moreover, we also discuss how the concept of a ’thermal-to-epithermal neutron station’ employing NT and NCS techniques could be used in an outdoor environment by combining a thermal-to-epithermal neutron station with a portable and compact laser-driven neutron source and a compact moderator assembly. We provide a short critical appraisal of the neutronic parameters of compact neutron source-moderator assemblies and contrast them with their counterparts characterising the Vesuvio plus beamline. An important conclusion from this comparison is that both types of neutron source-moderator assemblies can already support the experiments based on the idea of thermal-to-epithermal neutron station with similar neutron flux and neutron pulse resolution parameters, thus paving the way towards the application of the ’thermal-to-epithermal neutron station’ concept for the outdoor detection of illicit and hazardous substances. Therefore, the vast development of compact neutron sources (both, accelerator- and laser-based)^28,29^ opens a new possibility to develop sensors supplying all the mentioned experimental techniques. This has a big potential to greatly improve the sensitivity of neutron-based detection methods with no significant decrease in the sensor mobility. NCS, NT and NAA may be used with the same neutron source working in a mode with the moderator, and without it.

An alternative or supplementary method, especially in cargo scanning, could be augmenting the NAA with neutron transmission (NT) and Compton scattering (NCS) measurements. As recently proved by the work introducing the average functional group approximation (AFGA)^24,25^, the concurrent use of both methods may give information about the elemental content and the functional group present in the tested substance. This allows for distinguishing isomers, which would be a significant improvement concerning NAA used alone. In this work, we report the results of a feasibility study employing neutron transmission and Compton scattering for the detection of hazardous materials. To this end, we rely on a series of measurements performed using the VESUVIO thermal-to-epithermal neutron station at the ISIS neutron and muon spallation source near Oxford, UK, on a substance widely accepted as a surrogate of both explosives and drugs with similar elemental content and structure, the melamine (C$_3$H$_6$N$_6$)^15–21^. Apart from substance identification by concurrent application of NT and NCS, the experiments described in this work have been designed to determine the limits of detection (LODs) and quantification (LOQs).

In parallel, the application of the NCS technique in tandem with *ab initio* materials modelling allowed for a critical appraisal of nuclear quantum effects (NQEs) in melamine in an isotopic mass-resolved manner. NQEs were characterised by the widths of nuclear momentum distributions (NMD widths) that describe the local binding and confinement of isotopic species and the accompanying degree of nuclear quantum delocalisation (NQD) in space. To this end, the present work extends the VESUVIO experimental protocol previously established for the detection of hydrogen in the solid state^27^.

## Results

### Structure and stability of melamine: insights from *ab initio* modelling

Melamine (1,3,5-triazine-2,4,6-triamine) is a highly symmetric (D_3h_) and very stable heterocyclic aromatic molecule based on a flat six-membered C-N aromatic ring of s-triazine with three NH_2_ amino groups attached to each carbon. According to theoretical predictions, the flat C-N ring is similar to the hypothetical hexagonal-C_3_N_4_, whose hardness might exceed that of a diamond^30^. Being commonly substituted with NO_2_ groups, the s-triazine ring further represents a common structural motif found among molecular explosives^31^. In that sense, melamine is a widely accepted surrogate model, being closely related to the widely used energetic materials, *i.e.*, RDX (hexahydro-1,3,5-trinitro-1,3,5-triazine) and TATB (2,4,6-triamino-1,3,5-trinitrobenzene). Nevertheless, melamine forms highly explosive nitrate salts (*i.e.*, melamine dinitrate, MDN), maintaining a sufficient explosive potential similar to RDX^31,32^. Furthermore, owing to its high propensity to hydrogen-bonding formation, it represents an important tecton for supramolecular chemistry^33^ and a promising platform for energetic co-crystal engineering^34^.

**Figure 1:**
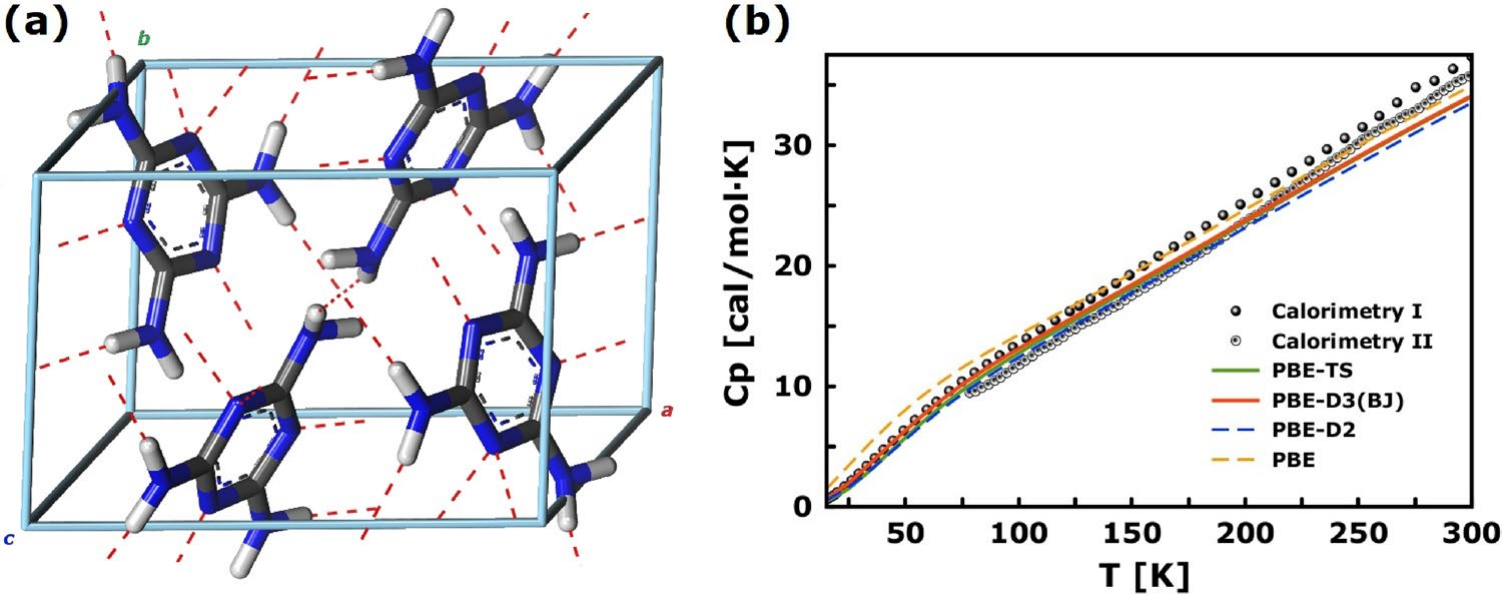
(**a**) The monoclinic phase of melamine (*P2*_1_/*a* with Z = 4) in stick representation, according to plane-wave DFT (PBE-TS) optimization at 0 K. Atom color coding: hydrogen (white), nitrogen (blue) and carbon (grey). Red dashed lines indicate hydrogen bonding. (**b**) Temperature dependence of the molar heat capacity for the *P2*/_1_*a* phase of melamine according to selected density functional theory approximations. The scatter plot comes from the experimental data of Stephenson^41^ (Calorimetry I) and Peng et al. (Calorimetry II)^42^. The theoretical results are based on harmonic phonon dispersion calculations presented in this work. See text for details.

Historically, melamine was the first-ever compound for which the least-squares refinement of a crystal structure was done^35,36^. Since 1941, the molecular and crystal structure of melamine has been thoroughly explored with multiple radiation-scattering techniques, including x-ray^36,37^, electron^38^, and neutron diffraction^39^ and continued to date with high-resolution techniques^33,40^. The general consensus from the structural refinement work has been that no polymorphs are formed. As originally determined by Hughes^35^, the crystal structure of melamine at room temperature is monoclinic (space-group *P2*/_1_*a*, see Fig. 1a)^33^. As confirmed with thermophysical data (see Fig. 1 b)^41,42^, the *P2*/_1_*a* phase persists down to cryogenic conditions, without any signatures of phase transition. Similarly, recent accurate and precise neutron diffraction experiments on the perdeuterated sample (–*d*_6_) confirm the phase stability up to 5 GPa, *i.e.*, far beyond the limits of pressures at ocean depths^33^.

Owing to high chemical and physical stability, the monoclinic phase of melamine constitutes a testbed, allowing the optimisation of first-principles calculations for accurate prediction of the neutron scattering observables. To date, the reports on the first-principles calculations on melamine have been sparse, particularly regarding the crystal environment. Vosegaard et al.^43^ and Grabska et al.^44^ reported some periodic DFT calculations with atom-centred orbitals using an original formulation of hybrid B3LYP functional, yet did not account for van der Waals (vdW) interactions. Fortes and co-workers reported an extensive structural analysis based on the dispersion-corrected semi-local plane-wave DFT scheme for an improved description of the cell parameters^33^. To further scrutinise the performance of the dispersion-corrected schemes, we follow the latter approach, providing a benchmark of the off-the-shelf vdW-corrections combined with the Perdew-Burke-Ernzerhof (PBE) functional^45^. To this end, we utilise the plane-wave DFT implementation using the Cambridge serial total energy package (CASTEP) in conjunction with pair-wise and many-body dispersion correction schemes proposed by Grimme^46–51^ and Tkatchenko and Scheffler^52–54^. The accuracy and efficiency of both strategies for energetic materials have been recently examined by Liu et al. and O’Connor et al. respectively, showing considerable improvement in terms of lattice energy and cell volumes compared to the original PBE formulation^55,56^.

Geometry optimisation in CASTEP, performed at the limit of T = 0 K, yields a structure summarised in Table 1, where the internal bond lengths agree with the experimental measures within the error bars^38^. Through the comparison between the experimental data reported for both hydrogenous and perdeuterated samples, no considerable isotope effects on the cell volume could be found. The *k*–point spacing of 0.05 1/Å was required to converge calculations of the cell constants. The structure optimised with dispersion-corrected DFT (DFT-D) compares favourably with the structure obtained from neutron single-crystal (SC) and high-resolution powder (HRP) neutron diffraction (ND) cryogenic data^40^, considering large errors in predicted cell parameters by the PBE functional. On the contrary, the standard pairwise PBE-D2 scheme visibly overestimates the influence of the vdW interactions, yet noting that the theoretical predictions at 0 K do not account for any vibrational contributions to thermal expansion. A considerable improvement is delivered in conjunction with modern pairwise correction schemes (PBE-TS, PBE-D3, PBE-D4). The more recent Becke-Johnson-damping version of DFT-D3, DFT-D3(BJ), provides improvement compared to the original zero-damping correction (hereafter, DFT-D3(0)). Further improvement is recognised with the most recent DFT-D4 scheme, which is adequately balanced regarding the cell constants description compared to any other approach, including the computationally demanding range-separated many-body dispersion model (PBE-MBD@rsCSC), accounting for nonadditive many-body contributions to the dispersion energy and the effect of dielectric screening. Contrary to previous findings for energetic materials^56^, the inclusion of the many-body contributions is not critical for the description of the crystal structure of melamine. This is further evidenced by a comparison between the PBE-D3(BJ) and PBE-D3(BJ)-ATM models, the latter accounting for a three-body term of the Axilrod-Teller-Muto (ATM) triple-dipole variety. Owing to their simplicity, standard PBE-D3(BJ) and PBE-TS corrections can then be selected as the methods of choice. The latter approach is favourable in the present case, owing to efficient and accurate internal implementation in CASTEP, allowing for analytical calculations of phonon properties according to the linear-response scheme.

It’s important to note that our optimisation at the limit of zero temperature does not consider any cell expansion effects due to the absence of vibrational entropy contributions to the cell volume. This limitation is significant and should be taken into account when interpreting our results. However, by examining the results obtained at both 0 K (PBE-TS) and finite temperatures using *ab initio* molecular dynamics simulations (PBE-TS AIMD 300 K), we can observe a systematic error in the description of the longest cell *a*-axis, while properly accounting for the cell expansion. This discrepancy underscores the need for further research and the potential for more accurate predictions when considering finite-temperature effects.

**Table 1:** Comparison of the lattice parameters and unit cell volume for the *P2*/_1_*a* structure of melamine as obtained from experimental data and semi-local plane-wave DFT predictions utilising different DFT-D formulations.

Method	a (Å)	b (Å)	c (Å)	$\alpha$ (°)	$\beta$ (°)	$\gamma$ (°)	Volume (Å$^3$)
SCND^40^ (14 K)	10.433	7.458	7.238	90.0	113.30	90.0	517.25
HRPND (–*d*_6_) ^33^ (15 K)	10.448	7.456	7.243	90.0	113.37	90.0	517.97
PBE-TS [0.07 1/Å]	10.592	7.409	7.152	90.0	113.28	90.0	515.54
PBE-TS [0.05 1/Å]	10.566	7.411	7.166	90.0	113.21	90.0	515.70
PBE-TS [0.02 1/Å]	10.566	7.411	7.166	90.0	113.21	90.0	515.70
PBE-TS-SCS [0.05 1/Å]	10.832	7.352	7.282	90.0	109.89	90.0	545.30
PBE-MBD@rsSCS [0.05 1/Å]	10.645	7.321	7.170	90.0	111.64	90.0	519.33
PBE-D4 [0.05 1/Å]	10.533	7.380	7.231	90.0	112.24	90.0	520.17
PBE-D3(BJ)-ATM [0.05 1/Å]	10.573	7.382	7.253	90.0	111.86	90.0	525.37
PBE-D3(BJ) [0.05 1/Å]	10.573	7.382	7.253	90.0	111.87	90.0	525.37
PBE-D3(0) [0.05 1/Å]	10.625	7.398	7.253	90.0	111.82	90.0	529.28
PBE-D2 [0.05 1/Å]	10.329	7.368	7.147	90.0	113.74	90.0	497.86
PBE [0.05 1/Å]	11.459	7.370	7.626	90.0	104.05	90.0	624.76
HRPND (–*d*_6_) ^33^ (297 K)	10.583	7.479	7.283	90.0	112.27	90.0	533.38
SCXRD^39^ (300 K)	10.606	7.495	7.295	90.0	112.26	90.0	536.68
SCXRD^36^ (300 K)	10.537	7.477	7.275	90.0	112.90	90.0	527.99
PBE-TS AIMD 300 K [0.05 1/Å]	10.686	7.496	7.247	90.0	113.21	90.0	533.47

The experimental data were obtained from single-crystal neutron (SCND)^40^ and x-ray (SCXRD)^36,39^ diffraction along with high-resolution time-of-flight neutron powder diffraction (HRPND) experiments^33^. The finite-temperature structural parameters obtained from AIMD simulations are compared with the room-temperature experimental data^33,36,39^. The *k*–point spacing given in square brackets [in 1/Å] defines the accuracy of the Brillouin zone sampling, which converges with the density mesh of 0.5 1/Å. The unit cell dimensions are in the units of Å. The cell volume is tabulated in Å$^{3}$.

Further assessment of selected DFT-D models is obtained by analysing the vibrational properties naturally reflected in the thermophysical data. Figure 1b compares the experimental data from adiabatic calorimetry with the theoretical predictions obtained with PBE and pairwise DFT-D schemes (PBE-D2, PBE-D3(BJ), and PBE-TS—the latter with and without the self-consistent screening, SCS). The reference experimental data slightly differ in absolute values yet match well with the results of theoretical predictions. While the low-temperature regime is driven by the population of the lowest-energy phonon modes of melamine, the range above 100 K shows a rather linear increase, suggesting the dominant role of the vibrational contribution to molar heat capacity in the whole temperature regime under consideration and so rather constrained molecular dynamics of the melamine units, in line with the experimental findings^39^. The theoretical results were based on harmonic calculations of the phonon band structure, yielding the vibrational density of states (VDoSs) compared in Section S1 of the Supplementary Materials (see Fig. S1). In general, local density approximation (LDA) leads to a well-known problem of overbinding, resulting in stiffer interisotopic force constants (IFCs), whereas reduction of the overbinding trends in the generalised gradient approximation (GGA) yields softening of the IFCs^57,58^. Similarly, a different description of the crystal environment is reflected in the force constants and the resulting VDoSs. The softening of the IFCs with the original PBE scheme results in an increase of the vibrational density at the lowest energies (see Fig. S1), providing an excursion from the expected temperature dependence of the molar heat capacity (see Fig. 1b). All the equilibrium models but DFT-D2 were found mechanically stable at the atmospheric pressure. The DFT-D2 model shows several imaginary modes (< 1 meV) beyond the $\Gamma$-point. Owing to the high numerical precision adopted in the present work, such a deficiency can be associated with the overbinding effects shown in Table rather than with numerical instabilities. While PBE-D2 provides slightly stiffer IFCs, resulting in lower heat capacity values compared to modern DFT-D schemes, it follows the trend revealed by PBE-TS and PBE-D3(BJ), which are generally indistinguishable in terms of predicted VDoSs and resulting thermalisation reflected in thermophysical data.

### Vibrational response

**Figure 2:**
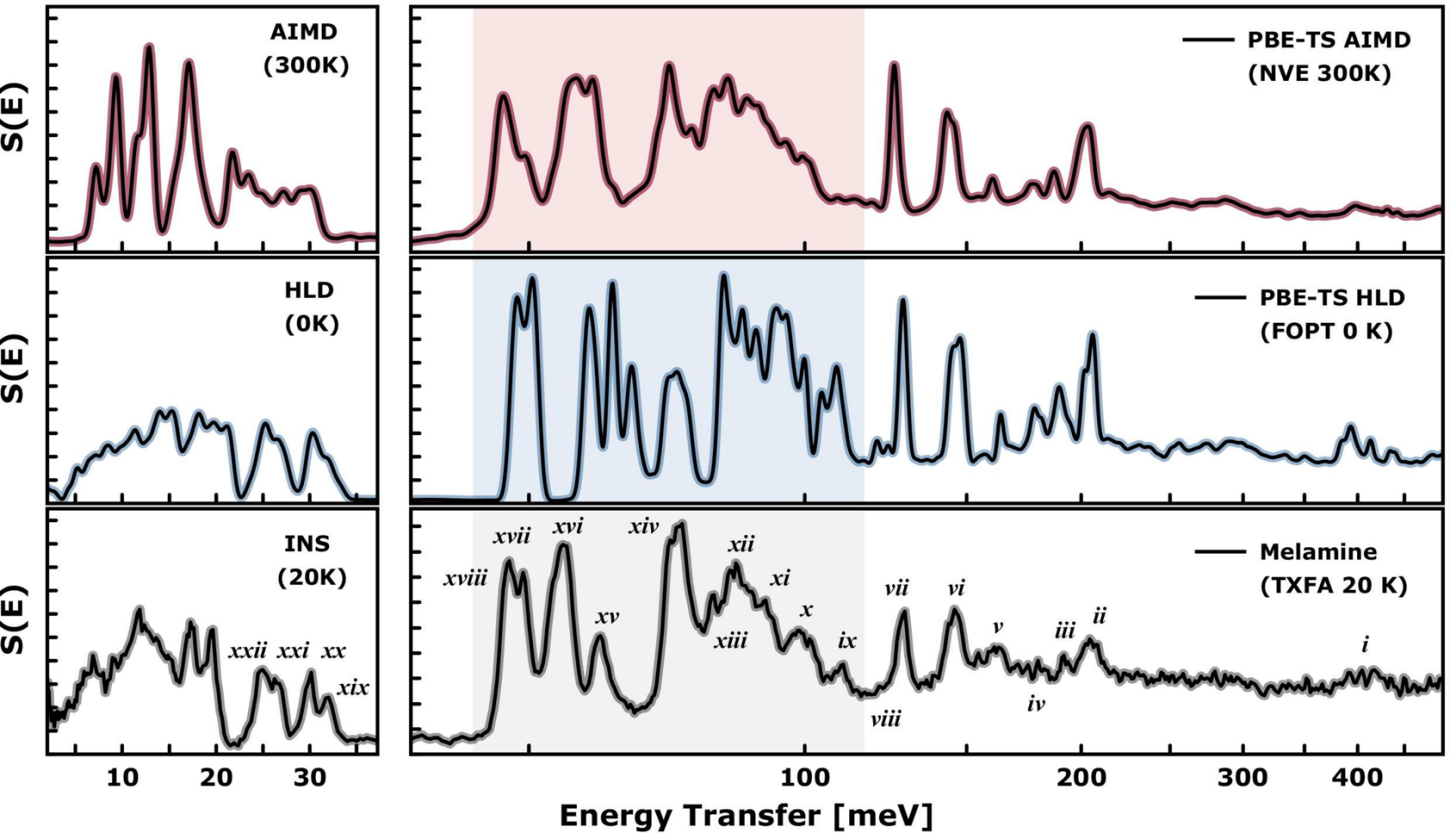
Experimental (bottom)^59^ and calculated (middle and top)^61^ INS spectra of the monoclinic (*P*2_1_/*a*) phase of melamine. The most prominent experimental bands are denoted by Roman numerals. The shaded spectral regime highlights out-of-plane deformations of the hydrogen-bonded species. See the main text for numerical details and the Supplementary Information (Table S2 and Figs. S5–S15) for a detailed assignment and visualization of associated vibrations.

To scrutinise further the vibrational properties of melamine, we refer to the experimental inelastic neutron scattering (INS) spectrum originally presented and analysed by Fernández-Liencres and co-workers (see Fig. 2)^59^. These data were collected at a temperature of 20 K using an indirect-geometry TFXA spectrometer at the ISIS Facility and are freely available for download from the INS Database^60^. A detailed inspection of nuclear dynamics behind the neutron scattering observables is further provided in the Supplementary Materials by referring to first-principles predictions. Sections S2 and S3 of the SI provide a detailed analysis of the underlying vibrations at the $\Gamma$–point and beyond, respectively.

Four melamine molecules building the unit cell give rise to 180 zone-centre modes, which are listed in Table S1 and analysed in detail in Figs. S2–S15. The *A*– and *B*–type symmetry modes further split into *gerade* (Raman active) and *ungerade* (IR) active species, where fourfold degeneracy is expected for each type of vibration. While mutually excluded in optical spectroscopy due to symmetry selection rules, all vibrations are allowed to be observed with INS spectroscopy. A detailed interpretation of both optical and INS spectra of melamine was presented by Fernández-Liencres et al.^59^ based on a discrete molecular model and scaled quantum-mechanical force fields. Here, for the first time, we present the vibrational analysis based on periodic DFT calculations. According to Fig. S1 and Table S1, twenty-four modes are of purely external character, expressing translational and rotational motions of the molecular centroids, heavily affected by intermolecular contacts. These lattice vibrations are spanned over the energy transfers below 22 meV, being visibly decoupled from the internal molecular modes, which are spread across the 24–440 meV range. In Fig. S3, the zone-centre modes are further decomposed into nuclear contributions with hydrogen-dominated vibrations above 50 meV. An indirect-geometry chemical spectrometer TXFA was originally optimised for the detection of proton dynamics, allowing the highlighting of vibrational excitations with strong projection on the hydrogen nuclei and delivering the hydrogen-projected VDoS. Thus, the TXFA spectrum (see Fig. 2) coincides with the modes characterised by the strongest proton contributions displayed in Fig. S3. A detailed assignment of the INS spectrum is shown in Table S2 and is accompanied by a schematic representation of the phonon eigenvectors (see Figs. S4–S15).

The upper panels in Fig. 2 show the theoretical INS spectrum of the monoclinic phase of melamine, according to harmonic lattice dynamics (HLD at 0K) and finite-temperature *ab initio* MD simulations (AIMD at 300 K). The most prominent experimental bands are denoted by Roman numerals, and quantitative spectral analysis is presented in Table S3 of the Supplementary Information. Concerning the crystallographic structure and previous spectroscopic analysis of melamine, Fig. 2 highlights the range of 50–110 meV, which has been recognised as particularly sensitive to selected methodology (see Fig. S1), being ascribed to proton motions in considerably anharmonic potential. According to Table S2, this spectral regime mainly reflects the out-of-plane deformations of the hydrogen-bonded NH_2_ group. The analysis of the anharmonic behaviour of melamine provided by Ozaki and co-workers delivered important conclusions on the extent of anharmonicity in this compound^44^. The authors found that the out-of-plane fundamental transitions are markedly more sensitive to out-of-plane inter-molecular interactions than the near-infrared (NIR) overtone transitions. A proper description of the molecular surroundings was found to be more important than the anharmonicity of its vibrations^44^. The NIR bands were mostly linked to in-plane vibrations, remaining surprisingly insensitive to the chemical environment, which fully agrees with the spectroscopic picture presented in this work^44^. While the s-triazine ring in melamine was found to be nearly planar and the three amine groups pyramidal^40^, the calculated barrier of inversion of the NH_2_ group is very low (< 1 kcal/mol)^62^, indicating possible disorder of NH_2_ groups at room temperature as well as tunnelling effects at the cryogenic conditions owing to the shallow potential energy landscape. The out-of-plane NH_2_ deformations can be recognised as sensitive probes of the crystallographic environment around the melamine molecule. The discussed regime has been described remarkably well using AIMD simulations, yet noting that these were performed at elevated temperatures of 300 K due to the inability of the Born-Oppenheimer MD simulations to account for the zero-point energy (ZPE) properly.

The presented spectroscopic picture confirms the credibility of the adopted computational models to be used for further predictions of the neutron Compton scattering observables. To scrutinise the phonon picture further, we refer to the lattice dynamics calculations beyond the centre of the first Brillouin zone (1BZ). The phonon dispersion calculation on the optimised unit cell structure yields phonon dispersion along 29 points in the 1BZ in the reciprocal space (see Fig. 16 along with Tables 3–5 in the SI). Little mode dispersion can be seen, a feature typical for molecular crystals with weakly interacting molecules in the unit cells. A pronounced gap in the mode energy is visible between ca. 225 and ca. 385 meV, separating the lower-lying modes from the high-energy modes in the region between 385 and 440 meV. These high-energy NH-group stretching modes contribute mostly to the values of the nuclear momentum distribution widths and kinetic energies of hydrogen and partially to their counterpart in the case of nitrogen (see Figs. 17–19 in the SI). The lower-lying modes contribute mainly to the NMD widths and kinetic energy values of carbon and nitrogen.

### Neutron Compton scattering

#### Nuclear momentum distributions

**Figure 3:**
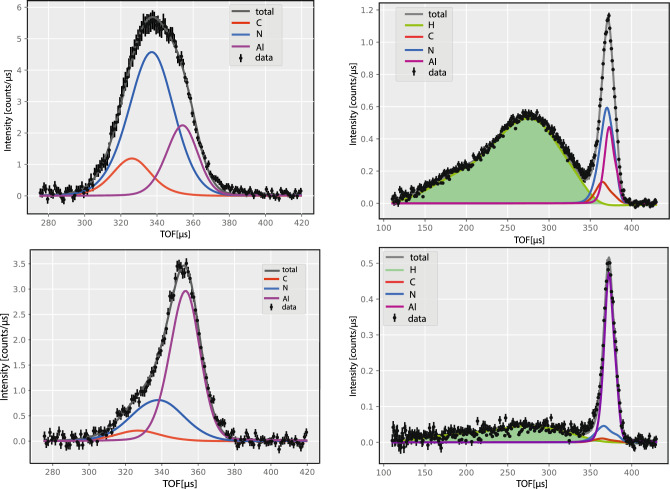
NCS data and fitting curves for the powder sample of melamine of mass *M* = 2.298 g (top panels) and mass *M* = 0.290 g (bottom panels). The data recorded at backscattering are shown in the left panels, and the data recorded at forward scattering in the right panels. Colour coding: the total (focused in the TOF domain) NCS data (black points and error bars), total fitted signals (solid red grey line), the fitted recoil peaks of hydrogen (solid green line and green shaded area), carbon (solid red line), nitrogen (solid blue line), and aluminium from the sample container (solid violet line).

The mass-resolved NCS spectra of powder melamine sample of mass *M* = 2.298 g and fitting curves in the TOF domain, summed over all backscattering and forward scattering detectors, are shown in Fig. 3 in the left and right panels, respectively. The mass-resolved NCS spectra of the powder melamine samples of mass *M* = 2.298 g and *M* = 0.290 g and fitting curves in the TOF domain, summed over all backscattering and forward scattering detectors, are shown in Fig. 3 in the left and right panels, respectively. The data recorded at backscattering are shown in the left panels, and the data recorded at forward scattering in the right panels.

**Figure 4:**
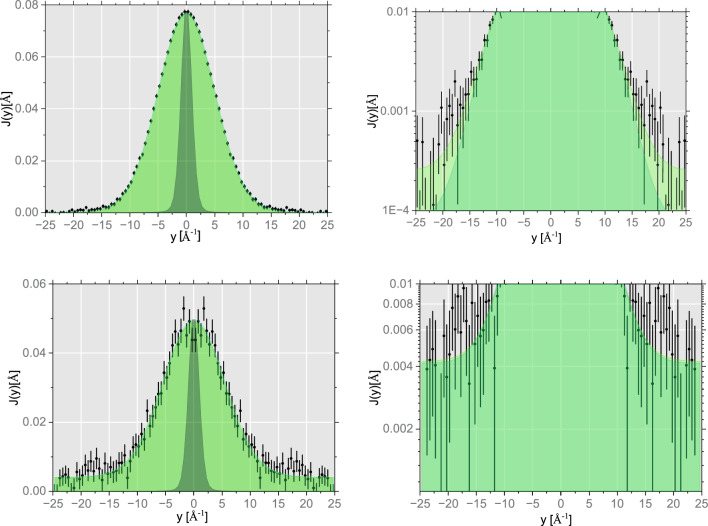
Powder sample of melamine of mass *M* = 2.298 g (top panels) and mass *M* = 0.290 g (bottom panels). The total, focused in the proton longitudinal momentum domain, *y*, proton NMDs (black points and error bars) and NMD fitting curves (solid red green lines) for the Gaussian (shaded green area) and multivariate Gaussian (shaded light green area) NMD models. Additionally, in the left panels, the instrument resolution functions for hydrogen in the longitudinal momentum space is are shown as a dark grey shaded area. In the right panels, the zoomed areas of proton NMDs are shown to emphasise the differences between the Gaussian and multivariate Gaussian model fits.

The mass-resolved nature of the recorded NCS spectra is clearly visible. In the TOF domain, the recoil peaks are ordered from the lowest to the highest isotopic mass. Due to the much higher values of the neutron momentum transfers in the backscattering regime, the mass resolution of isotopic species heavier than hydrogen is much better than in the case of the forward scattering (Fig. 3, left panels). In the backscattering data, the recoil peaks are ordered, starting from carbon (solid red curve) with the recoil peaks centred at ca. 325 $\mu$s, followed by nitrogen with the recoil peaks centred at ca. 335 $\mu$s, and aluminium from the sample container with the recoil peaks at around 355 $\mu$s. This picture justifies the data treatment strategy chosen for the analysis of the melamine NCS data, whereby the NMD widths of the isotopic species heavier than hydrogen are first fitted in backscattering, and then the averaged over all detectors values of the NMD widths are fixed in fitting the forward scattering data (Fig. 3, right panel), with the aim of the separation of the proton recoil peaks (solid green line) and further fitting of the proton NMD in the TOF and proton longitudinal momentum domains.

Owing to the excellent recoil peak separation of the protons from the recoil peaks of heavier isotopic species in the forward scattering NCS data, the fitting curves of carbon, nitrogen, and aluminium can be easily subtracted from the total recorded signal, and the remainder, being the proton recoil peaks, can be sequentially (detector-by-detector) transformed from the TOF to the proton longitudinal momentum domain, *y*, to form the proton NMDs, shown in Fig. 4. It can be seen that the quality of the data focused in the proton *y* space domain (expressed in terms of the signal-to-noise ratio and the sizes of typical error bars) is better than that of the data focused in the TOF domain. This is the direct consequence of the fact that, in the *y* space domain, the loci of the recoil peaks are aligned around the $y=0$ value for any given recoiling mass, whereas in the TOF domain, the recoil peaks move with the scattering angle (detector).

In the bottom panels of Figs. 3 and  4, the mass-resolved NCS data and the proton NMDs are shown for the melamine sample of mass *M* = 0.290 g, which was the smallest melamine sample considered in this work. In Fig. 3, it can be clearly seen that the overwhelming share of the total scattered intensity, in this case, is attributed to the neutron Compton scattering off the sample aluminium container, rendering the precise analysis of the recoil peaks of hydrogen, nitrogen and carbon in melamine difficult. Moreover, as clearly seen in Fig. 4, the precise analysis of the shapes of the proton NMD, involving the NMD model selection, is very difficult in the case of the sample of mass *M* = 0.290 g, due to poor signal-to-noise ratio and relative large error bars of the NMD data. It is for this reason that the NMD model selection and analysis have been performed for the melamine data recorded for the sample of mass *M* = 2.298 g.

**Figure 5:**
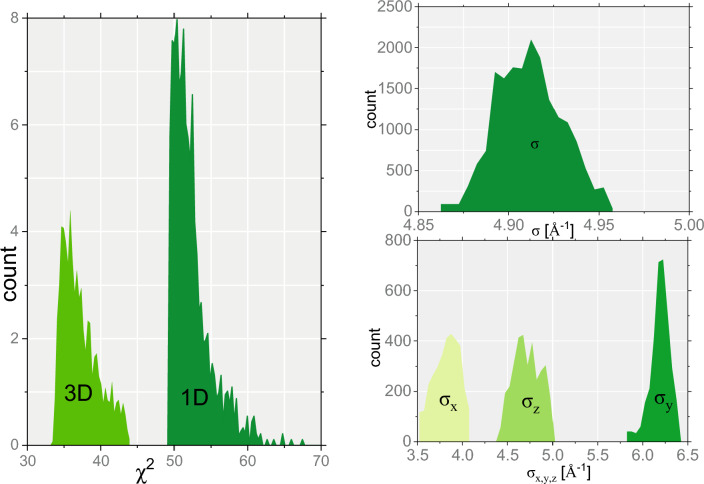
Powder sample of melamine of mass *M* = 2.298 g. The results of the selection process of the best-fitting model for the proton NMD data. The selection process has been performed based on the Fitting Algorithm for Bayesian Analysis of DAta (FABADA)^63^. Probability distribution functions for the $\chi ^2$ variable (left panel) and NMD widths (right panel) were calculated using the FABADA software for the Gaussian and multivariate Gaussian NMD models. The left panel shows the probability distribution functions for the absolute (not divided by the number of degrees of freedom) $\chi ^2$ values in the case of the multivariate Gaussian model (3D) and the isotropic Gaussian model (1D). The top right panel shows the probability distribution function of the width of the proton NMD, $\sigma$, as calculated for the isotropic Gaussian model. The bottom right panel shows the probability distribution functions of the widths, $\sigma _x$, $\sigma _y$, and $\sigma _z$, as calculated for the multivariate Gaussian NMD model.

The selection of the best-fitting model for the proton MDS NMD data shown in Fig. 4 has been performed based on the Fitting Algorithm for Bayesian Analysis of DAta (FABADA)^63^. In FABADA, a Monte Carlo (MC) algorithm is applied to randomly vary the fitting model parameters and accept those compatible with data and experimental error. The probability distributions of the chi-square figure of merit and model parameters are then calculated and used in the model selection process. FABADA has been successfully applied for model selection in fitting data in astrophysics^64^, dielectric spectroscopy^65^, neutron diffraction^66^, quasi-elastic^67^, and Compton scattering ^68^ neutron spectroscopy. Probability distribution functions for the $\chi ^2$ variable and NMD widths calculated using the FABADA software (available via Mantid computational environment) for the Gaussian and multivariate Gaussian NMD models are shown in Fig. 5.

In the case of the Bayesian analysis, the figure of merit in the model selection is the absolute $\chi ^2$ value, rather than its reduced (calculated per number of degrees of freedom) counterpart^63^. The $\chi ^2$ distributions, shown in the left panel of Fig. 5, clearly indicate that the multivariate Gaussian model is more likely to produce a good fit of the proton NMD data in melamine. Also, the distributions of the individual NMD widths in the multivariate Gaussian model, plotted in the bottom right panel of this figure, do not overlap, indicating a good quality fit. Indeed, whereas the central part of the proton NMD is fitted equally well by both models (as shown in the left panel in Fig. 4, the tails of the distribution are fitted better by the multivariate Gaussian model, as shown in the right panel in Fig. 4 (shaded light green area).

The values of the NMD widths and nuclear kinetic energy of protons, carbon, and nitrogen nuclei for the powder sample of melamine of mass *M* = 2.298 g obtained by fitting the experimental data to the isotropic Gaussian NMD model are contrasted with the *ab initio* predictions from the HLD and AIMD simulations in Tables 2 and 3, respectively.

**Table 2:** The experimental values of NMD widths of protons, carbon, and nitrogen nuclei for the powder sample of melamine of mass *M* = 2.298 g contrasted with *ab initio* predictions from the HLD and AIMD simulations, both obtained from the isotropic Gaussian model of the nuclear momentum distribution.

Nucleus	HLD prediction	AIMD prediction	Isotropic Gaussian fit
H	5.08	5.22	4.87 ± 0.02
C	13.54	14.17	9.2 ± 1.8
N	12.97	14.17	12.7 ± 1.2

NMD width values are given in units of Å$^{-1}$.

**Table 3:** The experimental values of the kinetic energy of protons, carbon, and nitrogen nuclei for the powder sample of melamine of mass *M* = 2.298 g contrasted with the *ab initio* predictions from the HLD and AIMD simulations, both obtained from the isotropic Gaussian model of the nuclear momentum distribution using Eq. 20.

Nucleus	HLD prediction	AIMD prediction	Isotropic Gaussian fit
H	160.5	169.5	147.5 ± 1.2
C	95.8	104.9	44.2 ± 17.3
N	75.3	89.9	72.2 ± 13.6

The kinetic energy values are given in units of meV.

There is a good agreement between the experimental and simulated data, especially in the case of hydrogen, where the instrument resolution function is best, thus allowing for better peak separation and resolution of fine details in the peak shape. However, another trend is visible for all three types of isotopic species: the systematic increase of the NMD width value from the experimental, through the HLD-simulated, towards the AIMD-simulated. Such trends have been reported before in NCS studies of condensed matter systems^69,70^. They can be attributed to the fact that both HLD and AIMD simulate nuclear species motion in local potentials, whereby classical (point-like) particles move between the classical turning points. Thus, both methods do not capture the nuclear quantum tunnelling and delocalisation effects that cause nuclei to continue their motions beyond the classical turning points^71^. Consequently, the HLD and AIMD-predicted values of the widths of position distributions are narrower than their experimental counterparts. Moreover, it is known that most lightweight nuclear species, even close to room temperature, are in ground states of their respective local potentials. Thus, by virtue of the Heisenberg uncertainty principle between position and momentum in the ground state, too narrow (with respect to the experimental counterparts) Gaussian (or nearly Gaussian in the case of moderately anharmonic local potentials sampled by the AIMD simulations) position distributions are accompanied by too wide momentum distributions^71^. The explanation of why the AIMD-predicted values of the NMD widths are highest is more complicated. In general, anharmonic potentials lead to the temperature dependence of the ZPE values and thus also the values of the NMD widths^72^. Only the simultaneous accounting for the NQEs and anharmonicity (by the application of either AIMD with the so-called ’quantum thermostats’ or the path-integral molecular dynamics (PIMD)) can lead to the correct prediction of the temperature dependence of the ZPE in the presence of anharmonicity^72^. Thus, most likely, the trend observed for the melamine in this work is a manifestation of the inability to account for the anharmonic and nuclear quantum effects simultaneously.

The values of the NMD widths of protons, carbon, and nitrogen nuclei for the powder sample of melamine of mass *M* = 2.298 g obtained by fitting the experimental data to the multivariate Gaussian NMD model are contrasted with the *ab initio* predictions from the HLD simulations in Table 4. In this case, the multivariate Gaussian fits of the experimental data were only performed for the proton recoil peaks, while the recoil peaks of all heavier isotopic species were fitted using the isotropic Gaussian models using the same fitting protocol as in the case of the results listed in Table 2. This reflects the current limitation of the VESUVIO instrument, whereby only lightweight nuclear species can have their NMDs resolved sufficiently to be able to fit it with non-Gaussian NMDs^22,23,26^.

**Table 4:** The experimental values of the NMD widths and excess kurtosis of protons, carbon, and nitrogen nuclei for the powder sample of melamine of mass *M* = 2.298 g contrasted with the *ab initio* predictions from the HLD simulations, both obtained from the multivariate Gaussian model of the nuclear momentum distribution.

Nucleus	HLD prediction				multivariate Gaussian fit				Isotropic Gaussian fit
	$\sigma _x$	$\sigma _y$	$\sigma _z$	c$_4$	$\sigma _x$	$\sigma _y$	$\sigma _z$	c$_4$	$\sigma$
H	3.98	6.03	5.01	0.044	3.8 ± 0.2	6.1 ± 0.2	4.7 ± 0.6	0.059 ± 0.011	—
C	12.0	14.3	14.1	0.009	–	–	–		9.2 ± 1.8
N	11.3	13.9	13.6	0.013	–	–	–		12.7 ± 1.2

All values of NMD widths in units of Å$^{-1}$. The values of the excess kurtosis, c$_4$, are dimensionless.

Table 5 lists experimental values of the kinetic energy of protons, carbon, and nitrogen nuclei for the powder sample of melamine of mass *M* = 2.298 g compared with *ab initio* predictions from the HLD simulations. In the case of protons, both experimental values and *ab initio* predictions are obtained from the multivariate Gaussian model of the nuclear momentum distribution using Eq. 20. The experimental values for carbon and nitrogen atoms are obtained from the isotropic Gaussian model and *ab initio* predictions from the multivariate Gaussian model.

**Table 5:** The experimental values of the kinetic energy of protons, carbon, and nitrogen nuclei for the powder sample of melamine of mass *M* = 2.298 g compared with *ab initio* predictions from the HLD simulations.

Nucleus	HLD prediction			Multivariate Gaussian fit			Isotropic Gaussian fit
	$E_k (x)$	$E_k (y)$	$E_k (z)$	$E_k (x)$	$E_k (y)$	$E_k (z)$	$E_k$
H	32.8	75.4	52.1	29.9 ± 3.1	77.2 ± 5.1	45.8 ± 11.7	–
C	25.1	35.6	34.6	–	–	–	44.2 ± 17.3
N	19.1	28.8	27.6	–	–	–	72.2 ± 13.6

In the case of protons, both experimental values and *ab initio* predictions are obtained from the multivariate Gaussian model of the nuclear momentum distribution using Eq. 19. The experimental values for carbon and nitrogen atoms are obtained from the isotropic Gaussian model and *ab initio* predictions from the multivariate Gaussian model. All values of kinetic energy are given in units of meV.

In the case of protons, the agreement between the HLD-predicted and experimental values of the NMD width and kinetic energy is reasonable, given the degree of experimental error. Additionally, the value of the excess kurtosis, $c_4$, for the protons, obtained using Eq. (7), can be compared between the experiment and HLD calculation. It can be seen that both values are very similar, taking into account the standard deviation of the value of $c_4$ calculated from the errors of the values of the fitted parameters $\sigma _x$, $\sigma _y$, and $\sigma _z$. Moreover, both values of $c_4$ are lower than 0.8, which signifies that the excess kurtosis of the proton NMDs may come from the NMD anisotropy due to harmonic (or nearly harmonic) local binding potential of the protons rather than from the anharmonicity of the potential alone^73^.

In terms of the degree of the anisotropy of proton NMD in melamine, modelled by the multivariate Gaussian function, one can trace its origins to the specific vibrational modes within the 1BZ in the melamine crystal. To this end, Eqs. 13 and  14 prove useful. Figures S18 and S19 in the Supplementary Information (SI) show the contributions of individual modes to the total kinetic energy values along the *x*, *y*, *z* directions in space and their contributions to the total kinetic energy values summed over all three directions in space, respectively. We can see that, in the case of the protons, the biggest contributions to the total kinetic energy are from the modes no. 157–180. Modes 173–176 contribute most to $\sigma _H(\widehat{x})$ and $\sigma _H(\widehat{z})$, whereas modes no. 177–180 to $\sigma _H(\widehat{z})$. Cartoons representing the isotopic displacements for particular modes in the $q=[0,0,0]$ ($\Gamma$ point) are shown in Figs. S4–S15 in the SI. Modes 157–160 involve large NH-group proton displacements nearly parallel (or antiparallel) to the crystal c-axis (z-direction) with some components along the a-axis (which is nearly parallel to the x-direction). Modes 161–165 and modes 167–169 have large displacement components along the y and z directions, and mode 166 along the z and x directions. Modes 170–172 have large displacements along the x and y directions. Modes 173-176 are characterised by large displacements along the x and z directions. Finally, modes 177–180 have large displacement components along all three directions in space. Given the fact that all those modes have the highest vibration energies, 383–440 meV in the $\Gamma$ point of the 1BZ (see Tables S1 and S2 and Figures 18–19 in the SI), in the harmonic picture, they carry the biggest amounts of the nuclear kinetic energy, and their anisotropy contributes the greatest to the proton NMD anisotropy.

Table 6 lists the experimental values of the force constants of protons, carbon, and nitrogen nuclei for the powder sample of melamine of mass *M* = 2.298 g contrasted with the *ab initio* predictions from the HLD simulations, both obtained using Eq. (21) within the isotropic Gaussian NMD model.

**Table 6:** The experimental values of the mean force constants of protons, carbon, and nitrogen nuclei for the powder sample of melamine of mass *M* = 2.298 g contrasted with *ab initio* predictions from the HLD and AIMD simulations, both obtained from the isotropic Gaussian model of the nuclear momentum distribution using Eq. (21).

Nucleus	HLD prediction	AIMD prediction	Isotropic Gaussian fit
H	0.160	0.160	0.160 ± 0.001
C	1.918	1.918	1.918 ± 0.001
N	2.238	2.238	2.238 ± 0.001

Force constant values are given in units of eV/Å.

Table 7 lists the experimental values of the force constants of protons, carbon, and nitrogen nuclei for the powder sample of melamine of mass *M* = 2.298 g contrasted with the *ab initio* predictions from the HLD simulations, both obtained using Eq. 21. In the case of protons, both experimental and simulated values are obtained from the multivariate Gaussian model of the nuclear momentum distribution. In the case of carbon and nitrogen, the values listed are calculated based on the isotropic Gaussian model for the NMD.

**Table 7:** The experimental values of the force constants of protons, carbon, and nitrogen nuclei for the powder sample of melamine of mass *M* = 2.298 g contrasted with the *ab initio* predictions from the HLD simulations, both obtained using Eq. (21).

Nucleus	HLD prediction			Multivariate Gaussian fit			Isotropic Gaussian fit
	$\sigma _x$	$\sigma _y$	$\sigma _z$	$\sigma _x$	$\sigma _y$	$\sigma _z$	$\sigma$
H	0.160	0.161	0.160	0.159 ± 0.001	0.160± 0.001	0.160± 0.001	–
C	1.918	1.918	1.918	–	–	–	1.918 ± 0.001
N	2.238	2.238	2.238	–	–	–	2.238 ± 0.001

In the case of protons, both experimental and simulated values are obtained from the multivariate Gaussian model of the nuclear momentum distribution using Eq. 21. In the case of carbon and nitrogen, the values listed are calculated based on the isotropic Gaussian model for the NMD. Force constant values are given in units of eV/Å.

For both NMD models used to fit the NCS data, the experimental values of force constants and their counterparts, obtained from theoretical predictions (Table 6 and Table 7), do not depend on the values of the NMD widths, for a given isotopic species at room temperature. This is a consequence of the approximations employed in the derivation of the expression for the magnitude of the mean force constant based on the PIMD computational scheme^74^. Interestingly, the PIMD scheme used to derive the expression for the mean force constant does not rely on the harmonic approximation^74^, but the values of the magnitudes of the force constants obtained for the HLD scheme end up being very similar to their counterparts obtained from the inherently anharmonic (especially at room temperature) AIMD scheme.

#### Limits of detection and quantitation as observed by the neutron Compton scattering

In the case of hydrogenous analytes that are dissolved (or immersed) in water, the NCS spectra will contain proton recoil peaks stemming from at least two different chemical environments that are difficult to resolve without exact prior knowledge of their respective NMD widths. Such prior knowledge would require dedicated *ab initio* simulations of both the solute and the solvent molecules, thereby complicating the detection protocol. Thus, in order to determine the LOD and LOQ values for the melamine in a realistic setting, the recoil peaks of nitrogen nuclei instead of protons should be analysed. It is worth emphasizing that, unlike in many other analytical techniques, the NCS spectra can be fitted under a series of constraints due to the prior knowledge of the analyte sought. The recoil peak positions of all constituent isotopic species are fixed in the TOF domain due to the conservation of energy and momentum in the impulsive neutron Compton scattering process. Secondly, the recoil peak intensity ratios can be fixed during fitting using the stoichiometry of the analyte. Finally, the recoil peak widths can be constrained from below by the values of the NMD widths corresponding to Maxwell-Boltzmann momentum distributions^23,26^.

Usually, the recoil peak intensities for individual isotopic species are output as relative contributions to the total signal. However, if the recoil peak intensity needs to be plotted versus the analyte mass or concentration for the determination of the LOD and LOQ values, the procedure needs to be modified to output the absolute values of the integrated recoil peak intensities^27^. In the specific case of melamine, numerical integration was performed of the sums (over the range of all detector spectra in the backscattering regime) of the fitted curves of the nitrogen recoil peaks and the integrals were plotted versus sample masses. Following the LOD and LOQ protocol established in the earlier NCS work^27^, the highest values of the integrals (corresponding to the highest values of sample masses) were used for the calculation of the sensitivity parameter, *S*:

1 $$\begin{aligned} S = \frac{[\text {nitrogen in sample mass -- nitrogen in blank mass}]}{[\text {nitrogen in sample counts -- blank counts}]} \end{aligned}$$

**Figure 6:**
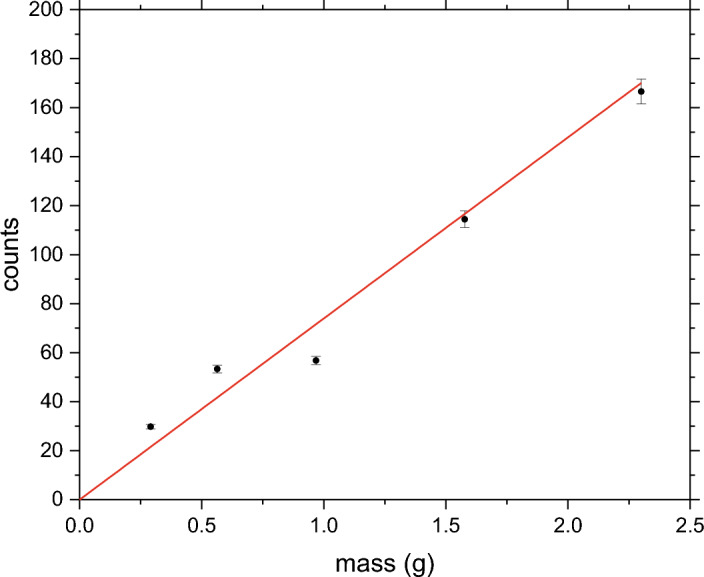
The sensitivity curve for the melamine sample. Total integrated intensities of the nitrogen recoil peaks recorded in backscattering NCS experiments are plotted as a function of the sample mass. The data are plotted as solid black points with error bars. The fitted linear relationship between the counts and the mass is shown as a solid red line. See text for details.

The value of the blank counts was set to zero, reflecting the choice of the blank measurement (empty aluminium sample container). As the blank measurement did not have any neutron detector counts corresponding to the neutron scattering on nitrogen, the value of the nitrogen mass corresponding to the blank measurement was set to zero, with the standard deviation of one count that was established by a series of twenty independent NCS measurements. The nitrogen mass in the melamine ($C_3H_6N_6$) sample of mass 2.298g is $\frac{6 \times 2.298}{126.123}\times 14.01 = 1.5305~\text {g}$. Thus, the value of $S = \frac{[1.5305-0]}{[167.03-0]}=0.00916$ [g/counts]. The same value can be obtained from linear regression by fixing the value of the intercept to zero (see Figure 6).

Following the methodology already described in our earlier work on the limits of detection and quantitation in the NCS technique^27^ and using the value of *S* and the standard deviation of the blank measurement, one obtains the value of the LOD of $S \times 3 \times 1 = 3 \times S$ and the value of the LOQ of $S \times 10 \times 1 = 10 S$. Using these two relations, the values of the LOQ and LOQ are 0.027 and 0.092 grams, respectively.

### Neutron transmission and total neutron cross-section

#### Total neutron cross-section and nuclear quantum dynamics

**Figure 7:**
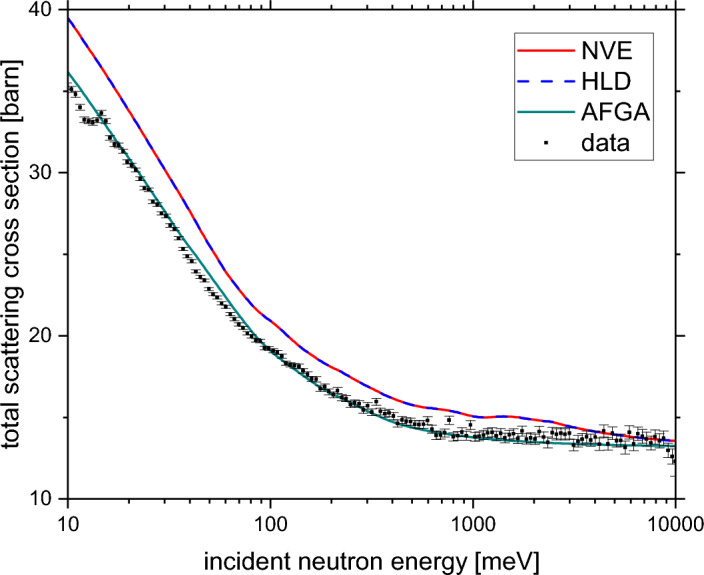
The total neutron cross-section data obtained from the incident neutron energy-dependent sample transmission of the melamine sample of mass of 2.298 g (solid black markers with error bars) and the results of the *ab initio* simulations of the total cross-section: HLD simulation (the solid dashed blue line), AIMD simulation (solid red line), and AFGA approximation (solid green line).

Figure 7 shows the total neutron cross-section data obtained from the incident neutron energy-dependent sample transmission of the melamine sample of mass of 2.298 g and the results of the *ab initio* simulations of the total cross-section. The *ab initio* simulations have been performed within the multi-phonon expansion (MPE) formalism using the underlying apVDoSs of carbon, nitrogen and hydrogen obtained from the HLD, AIMD and AFGA approximation.

Qualitatively, the overall shape of the incident neutron energy dependency of the total scattering cross section is well approximated by all three types of  *ab initio* simulations. However, the AFGA approximation describes the experimental data quantitatively and accurately, and there is a systematic discrepancy between the HLD and AIMD results on one side and the results of the AFGA approximation on the other. The reason for such a discrepancy lies in a way in which the MPE approximates the experimental total scattering cross-section data^24^. For systems where the AFGA approximation describes well the experimental data, the leading contribution to the total scattering cross-section stems from the apVDoS of the hydrogen and the contributions from the other types of isotopic species can be approximated as total free scattering cross-sections being independent of the dynamics of the heavier isotopic species^24^. Moreover, in the MPE, the leading term contributing the most to the total neutron cross-section at lower incident neutron energies is the contribution from the scattering process involving a single phonon^24,75,76^. The single-phonon term, due to the $\frac{1}{\omega }$ expression, favours the contribution from the low and medium energy parts of the apVDoS of the hydrogen. The low-to-medium energy parts of the apVDoS of the hydrogen do not differ markedly when simulated using the HLD and AIMD approaches, which explains why the total neutron cross-section curves simulated using these two methods are almost identical in Fig. 7. Moreover, the high-energy part of the hydrogen apVDoS is where the bulk of the contribution towards the magnitude of the proton kinetic energy and the widths of the proton momentum distribution in melamine comes from. Thus, the discrepancies in the values of these two observables are still consistent with the lack of discrepancies between the HLD and AIMD-based predictions of the total scattering cross-section curves.

#### Limits of detection and quantitation as observed by neutron transmission

**Figure 8:**
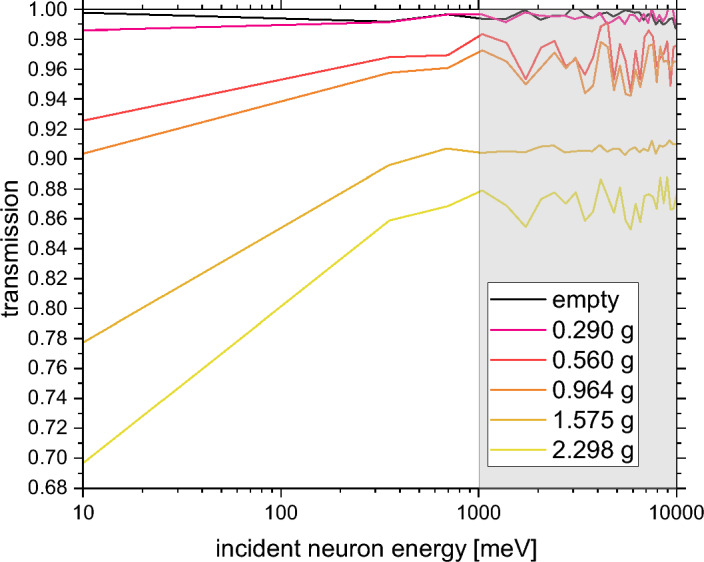
A series of incident neutron energy-dependent melamine sample transmission experiments were performed for samples with decreasing mass. The shaded area, corresponding to the epithermal incident neutron energies, was chosen to calculate the average sample transmission and scattering power values as a function of the sample mass. Additionally, the empty aluminium container transmission curve is shown as a solid black line.

A series of incident neutron energy-dependent melamine sample transmission experiments were performed for samples with decreasing mass. The results of this series of experiments are shown in Fig. 8. Additionally, the empty aluminium container transmission curve is shown. The shaded area, corresponding to the epithermal incident neutron energies, 1–10eV, was chosen to calculate the average sample transmission and scattering power values as a function of the sample mass. It is clearly seen that as the melamine sample mass decreases, the sample transmission in the epithermal region decreases. However, in the case of the samples of masses of 0.560 and 0.964 g, the plateau regions in the transmission curves, corresponding to the epithermal incident neutron energies, are mostly at the same level. Moreover, the plateau of the transmission curve of the melamine sample of mass of 0.290 g is almost at the same level as its counterpart obtained from the transmission measurement of the empty aluminium sample container.

Looking at Fig. 8, it appears that there is a higher sensitivity of the incident neutron energy-dependent transmission to the sample mass at lower neutron energies. Thus, it would be tempting to consider using the low-energy regions of the transmission curves for the determination of the LOD and LOQ values. However, using epithermal incident neutron energy regions of the neutron transmission curves to determine LOD and LOQ is dictated by the context of the particular application of the NT method, i.e. environmental detection of illicit substances. Considering that the neutron transmission-based detection will have to be performed at different temperatures and pressures (e.g. different locations at the sea bed and depths below the sea surface), one has to assume that the sample structure and dynamics will change with the changing external stimuli. Such changes may involve phase transitions or simple compression and expansion of the sample structure, both leading to changes in the atom-projected vibrational densities of states of the constituent atomic species. Such changes would change the shapes of the transmission curves at lower energies, which would complicate the data analysis protocols, including the instrument calibration and the determination of the LOD and LOQ if these values were to be established from the low-energy regions. Contrary to the low-energy regions, in the epithermal incident neutron energy region, the shapes of transmission curves are solely determined by the sample stoichiometry weighted by the total free scattering cross-sections of the individual atomic species, rendering the epidermal regions of the transmission curves independent on the environmental conditions.

**Figure 9:**
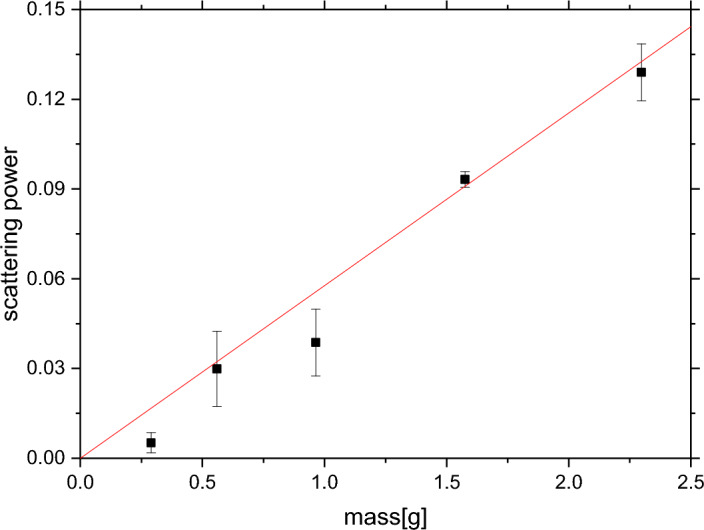
The sensitivity plot for the calculation of the values of the LOD and LOQ for the detection of melamine using the technique of incident neutron energy-dependent sample transmission. The values of the sample scattering power, S = 1–T(1–10 eV), have been calculated as weighted averages of the transmission curves in the epithermal incident neutron energy region and plotted as a function of the melamine sample mass.

In order to calculate the values of the LOD and LOQ of melamine as obtained from the incident neutron energy-dependent sample transmission, the values of the sample scattering power were calculated as 1–T(1–10eV), where T(1–10eV) are weighted averages of the transmission curves in the epithermal incident neutron energy region. The transmission curves are shown in Fig. 9 as a function of the melamine sample mass. As expected from the analysis of the transmission data shown in Fig. 8, the values of the scattering power obtained for the melamine samples of masses 0.560 and 0.964 grams are very similar, and the value of the scattering power for the melamine sample with the smallest mass of 0.290 g is almost at the level of its counterpart calculated for the blank (empty aluminium container). The sensitivity curve for the calculation of the values of LOD and LOQ has been obtained using data shown in Fig. 9 through linear regression. Following the methodology already applied in the case of the calculation of the values of LOD and LOQ for the NCS technique (see Fig. 6), the value of the sensitivity parameter, S, can be obtained as an inverse of the value of the gradient obtained from the linear regression by fixing the value of the intercept to zero, S = 1/(0.058 ± 0.003) [gram] = 17.24 ± 0.89 [gram]. The standard deviation of the measurement of the scattering power of the blank (empty aluminium container) was calculated from the scattering power data collated in the epithermal incident neutron energy region of 1–10 eV and yielded a value of 0.003. Following the methodology described in reference^27^, one obtains the value of the LOD of $(17.24 \pm 0.89) \times 3 \times 0.003 = 0.16 \pm 0.01$ grams and the value of the LOQ of $(17.24 \pm 0.89) \times 10 \times 0.003 = 0.52 \pm 0.04$ grams.

## Discussion

The quantitative and non-destructive molecule-selective detection of hazardous materials remains a formidable experimental and methodological challenge for a variety of reasons. Firstly, the target molecules do not exist in a vacuum but are often embedded in solid-state environments, suspended or dissolved in solutions or solvents, which requires spectroscopic and structural probes that are able to operate in non-transparent media. Secondly, modelling the structure and dynamics of such systems requires sophisticated methods that can cope with relatively large numbers of atoms and degrees of freedom in the presence of non-negligible dynamical, electric, and/or magnetic couplings between the target substances in their immediate chemical environments.

Neutrons offer an attractive option in this context because they are deeply penetrating and relatively locally interacting with the target systems and are, in general, considered non-destructive probes^15,77^. The non-destructive nature of neutron scattering is very desirable when very restrictive experimental protocols are imposed (in the case of the detection of hydrogen in cultural artefacts, valuable specimens, or, indeed, hazardous materials)^15,27,77^. However, neutron-based structural and dynamic probing of molecular and solid-state systems aiming at the detection of specific substances is not free from challenges. Since the bound scattering cross-section of the hydrogen (82.02 barns)^78,79^ is the largest of all elements in the periodic table, the use of neutrons as probes is dominated by elastic and inelastic scattering off this isotopic species. This is not always a desired feature, especially when both the target substance to be detected and its immediate chemical environment contain hydrogen. In hydrogenous systems, not only is the elastic (inelastic) neutron scattering dominated by the static (dynamic) response function of the hydrogen but also both the elastic and inelastic response functions have a collective character, whereby all degrees of freedom of all species at different sites contribute to the scattering intensity^77^. Moreover, in a natural environment, techniques such as isotopic or site-selective isotopic labelling or nuclear spin polarisation cannot be applied to create a contrast between the system to be tackled and its environment.

Due to recent advances in instrumentation and methodology at the VESUVIO beamline at the ISIS neutron and muon source, neutron Compton scattering has entered the mainstream of non-invasive, selective detection of isotopic species in solid-state systems and molecules^27,77^, and it has done so due to a combination of unique features of this particular inelastic neutron scattering technique. NCS operates in an impulsive, incoherent scattering regime. It has several very important ramifications^22,23,77^. Firstly, the NCS scattering intensity is proportional to the total (coherent plus incoherent) bound scattering cross-section, thus maximising the sensitivity of the neutron detection of all isotopic species present in a system under consideration. Secondly, the total NCS spectrum is a sum of recoil peaks of all constituent isotopic species. Finally and most importantly, in the context of this particular work, the recoil peaks of hydrogen are only observed in the forward scattering regime. This last feature, allowing the selection of the contribution to the total signal from hydrogen by the analysis of the forward scattering data, has motivated the pioneering work on the limits of detection and quantitation of hydrogen by the NCS^27^.

In this work, the same kinematics-based detection scheme, motivated by our original work on hydrogen detection using the peculiarities of the impulsive neutron Compton scattering, the neutron Compton backscattering kinematics has been used to select the Compton scattering off isotopic species other than hydrogen (concretely, the nitrogen that is universally present in the explosives and their surrogates) in the backscattering regime. There are several reasons behind the choice of the backscattering geometry for the NCS-based detection of hazardous materials and their surrogates. First of all, the total bound scattering cross-section of nitrogen is ca. seven times lower than its hydrogen counterpart^78,79^. Thus, in the forward scattering geometry, the NCS spectra of the hazardous materials would contain dominating hydrogen recoil peaks that would most likely at least partially overlap with their nitrogen counterparts, rendering the data analysis difficult. The isotopic composition of most hazardous materials includes, besides hydrogen and nitrogen, only a handful of types of isotopic species, such as oxygen or carbon, having total bound neutron cross sections at least two times smaller than nitrogen^78,79^. Thus, the backscattering NCS spectra will most likely be dominated by the nitrogen recoil peaks. Secondly, the NCS forward scattering geometry-based hazardous materials detection would require placing detectors behind the target, which may prove difficult, if possible, in the presence of obstacles in the natural environment. In backscattering, the detectors are placed on the same side of the target as the incoming neutron beam, which is a much more practical solution in a natural environment, where a compact neutron source would be placed on a drone and detect neutrons Compton scattered backwards from an object lying on a surface or buried in the ground.

The NCS backscattering-based hazardous materials detection needs to be discussed in the context of the concept of the thermal-to-epithermal neutron station pioneered by the VESUVIO beamline at ISIS^22,23,77^. Namely, the availability of as many as five different neutron-based techniques that can be concurrently applied owing to the broadband incident neutron beam gives a unique opportunity to employ more than these techniques simultaneously for the target substance detection and quantification. In this work, due to the presence of a complicated sample environment (the closed-cycle refrigerator and sample containers that are made largely of aluminium), the detailed analysis of powder neutron diffraction data collected in the backscattering geometry was difficult due to the presence of spurious peaks due to elastic thermal neutron scattering off aluminium overlapping with Bragg peaks due to neutron diffraction in the melamine sample. However, such analysis of backscattering diffraction data would be possible in a compact neutron source-based device (e.g. drone) in the presence of a hazardous material in the natural environment. Finally, incident neutron energy-dependent neutron transmission on VESUVIO can also have its equivalent working with a compact neutron source. The standard NT setup with the transmission detector placed behind the sample is not practical in the case of hazardous material detection in the environment. However, two modifications are possible. The sample may be first identified using alternative techniques (such as NCS), and then – only for the sake of the quantification – the substance can be moved from its original position and placed between the incident and transmitted beam monitors to determine the amount of neutron transmission. The second possibility, inspired by the present VESUVIO instrument calibration protocol^80^, is to use a plane slab made out of lead that reflects the neutrons, scattering them back elastically towards the sample placed between the lead reflector and the second neutron monitor.

The recent instrumentation advances that have enabled the thermal-to-epithermal neutron station VESUVIO plus to enter the mainstream of material science would not have sufficed to advance the concept of an outdoor detection of illicit and hazardous substances if it was not for the progress in the field of compact neutron sources and compact moderators converting incident neutrons with mega-electronvolt (MeV) energies into epithermal and thermal neutrons^29,81–92^. Coincidentally, it was at the ISIS Pulsed Neutron & Muon Source where the milestone was achieved of experimental demonstration of a compact epithermal neutron source based on a high-power laser^81^. The authors demonstrated that laser-driven fast ( MeV) neutrons can be efficiently moderated to epithermal energies with intrinsically short burst durations. In a proof-of-principle experiment using a 100 TW laser, a significant epithermal neutron flux of the order of 10$^5$ n/sr/pulse in the energy range of 0.5–300 eV was measured, produced by a compact moderator deployed downstream of the laser-driven fast neutron source^81^. In the original setup, the epithermal neutrons were measured at a distance of 2.58 m from the moderator using $^3$He detectors in the TOF mode, the same type of detectors that had been originally used at the VESUVIO predecessor 30 years before^22^. Since then, VESUVIO has undergone a significant transformation regarding its neutron detection setup and the water moderator it is facing^23,93^. These changes were driven by the concept of the epithermal-to-thermal neutron station, as the interest in concurrent transmission, diffraction and Compton scattering measurements grew as the result of the removal of one of the two poisoning Gd foils within the water moderator in the target station 1 (TS-1) in February 2016, increasing the effective volume where neutrons are moderated and the thermal neutron flux at the beamline^93^. After the water moderator upgrade, the neutron flux measured at VESUVIO plus is 104 n/s/sr/meV and 102 n/s/sr/meV in the thermal and epithermal incident neutron energy regions, respectively^93^. At present, the VESUVIO plus instrument is equipped with the YAP detectors $\gamma$-ray detectors in the forward scattering geometry and $^6$Li doped lithium glass GS20 detectors for the detection of the back-scattered neutrons^23^. At VESUVIO plus, the sample is placed ca. 11 metres from the moderator position, and the forward and back-scattering detectors are placed ca 0.5 metres from the sample position^23^. The YAP detectors are known to have lower count rates than the lithium glass counterparts, but they are much less prone to saturation, which was the reason why they have been chosen to detect neutrons forward scattered off the hydrogen in the Compton scattering regime for this particular detector setup and neutron flux^23^. This Vesuvio plus setup must be contrasted with the laser-driven compact neutron source described in Ref.^81^. In that latter setup, the laser pulses were focused onto 10 $\mu$m gold targets. The fast neutrons were produced by impinging the ions from the gold foil (pitcher) onto a 2 cm thick block of lithium (catcher), placed 5 mm away from the pitcher. At a distance of 11 cm from the catcher, a dedicated compact moderator was placed. Within the overall moderator size, the main component was a 5 cm 5 cm 4 cm block of high-density polystyrene, which was designed to slow down MeV neutrons to the epithermal range, with the mean free path of 1 MeV neutron being 3 cm. From the side of the neutrons entering the moderator, 2.4 cm thick lead and 1.2 cm thick tungsten plane slabs were placed, mainly serving as reflectors to the moderated neutrons produced by the polystyrene block. The polystyrene, tungsten, and lead blocks were housed in a cm-thick aluminium structure, surrounded by 1.5 cm thick lead to reduce neutron leakage from the side walls of the moderator. In order to produce a pure epithermal beam and filter the lower energy neutrons, a 2 mm cadmium sheet was placed at the exit plane of the moderator^81^. This moderator assembly resulted in an anisotropic neutron flux past the moderator with the sub-MeV neutrons efficiently moderated to the epithermal range inside the polystyrene block. However, despite the anisotropy, the central part of the moderated beam cross-section in such an assembly would be of sufficient size (5 cm by 5cm rectangular area) to cover the typical plane-slab sample container used presently on Vesuvio plus with a relatively homogenous epithermal neutron beam^81^. According to the specification given in Ref.^81^, the neutron flux per unit lethargy of such beam would be relatively stable (at the level of 10$^{-5}$ dn/du/cm$^2$) across the epithermal neutron energy range. Due to the high sensitivity of the $^3$He detectors, the gamma flash produced by the laser interaction saturated the signal at early times, and only neutrons of energy up to 300 eV could be detected in a TOF mode^81^. Thus, if an epithermal-to-thermal neutron station employing $^3$He detectors were to work with a compact neutron source of that type, the range of epithermal neutrons would be constrained from above by the value of 300 eV, which is sufficient not only for the incident neutron-dependent transmission and Compton scattering experiments but also for the neutron resonant transmission and capture analysis to be conducted concurrently on the same sample^23^. However, depending on the epithermal neutron flux levels and specific assemblies of future compact neutron sources (metre-size $^3$He detectors are often used in such source-moderator-detector assemblies^92^), epithermal-to-thermal neutron stations could use $^6$Li doped lithium glass GS20 neutron detectors or YAP $\gamma$ detectors with gold foils converting neutron counts into $\gamma$ rays.

Further progress in the design of target and moderator assemblies working with compact neutron sources was achieved at the ISIS Pulsed Neutron & Muon Source a few years after the original work by Mirfayzi et al.^92^. The authors reported the production of an intense burst of thermal neutrons from a laser-driven miniature neutron source. The novel innovative solution for the target and moderator assembly involved a $^7$Li converter embedded in the moderator housing, which made a compact design and allowed an efficient coupling of fast neutrons into the moderator^92^. The newly designed moderator was employed together with a proton beam produced in the Target Normal Sheath Acceleration (TNSA) mechanism. By bombarding secondary targets (e.g., blocks of lithium, Beryllium, or deuterated plastic) using the ions produced owing to the TNSA mechanism, nuclear reactions produced high fluxes of beamed neutrons with sub-MeV energies^92^. Such neutrons can be efficiently moderated to thermal energies, compared to the multi-MeV neutrons that drive the thermal neutron sources at the spallation facilities. Moreover, deploying commonly used moderator materials (such as light and heavy water and polyethene) at room temperature, a significant flux of the order of 10$^6$ n/sr/pulse of thermal (1 meV–0.5 eV) and epithermal (0.5–65 eV) neutrons was measured^92^. Additionally, neutronic simulations of the moderator performance showed that the moderation to thermal energies reaches an optimum for moderator thicknesses of a few centimetres.

Neutron resonance spectroscopy (NRS), using epithermal neutrons for the elemental analysis in a plethora of solid state systems, has also been realised with compact laser-driven neutron sources coupled with dedicated compact MeV-to-epithermal neutron moderators^29,84,85^. Zimmer et al.^84^ developed a neutron moderator and analysed epithermal neutrons transmitted through the samples of tungsten and tantalum. Yogo et al.^29,85^ performed the laser-driven NRS with a single pulse of neutrons generated by a single laser shot. In their setup, the neutron detector for the TOF analysis was located 1.8 m downstream of the compact neutron source. In general, the neutron pulse is expanded as the moderator thickness is increased while the number of moderated neutrons is enhanced. In order to provide sufficient resolution with the 1.8-m beamline, Yogo et al. designed a compact moderator to compromise the duration and the number of epithermal neutrons. The Berylium secondary target (10 mm in thickness, 5 mm in diameter) was surrounded by the moderator made from HDP, which had a thickness of 30 mm. The neutron pulse duration, as measured behind the moderator, was estimated by a Monte-Carlo simulation to be 0.6 $\mu$s at the incident neutron energy of 5 eV. These are important developments in the context of further development of the concept of the thermal-to-epithermal neutron station, such as Vesuvio plus. Although not employed for the work presented here, Vesuvio plus beamline also offers the NRS method, and the NRS method on Vesuvio plus comes in three different flavours^23,94–97^: (i) as neutron resonant capture analysis using YAP detectors in forward scattering geometry, (ii) neutron resonant transmission analysis using $^6$Li glass monitors, and (iii) prompt gamma activation analysis using a dedicated high-purity Germanium detector. On Vesuvio plus, the sample placed at 11 metres from the water moderator asymmetrically poisoned with a Gadolinium foil produces neutron pulses of duration of 0.4 $\mu$s at the incident neutron energy of 5 eV as confirmed by Monte Carlo simulations. Thus, already at present, the neutronic performance of compact neutron moderators and sources is almost at par with the specification of the Vesuvio plus beamline working with the ISIS neutron and muon spallation sources, and both types of assemblies can be used for the NRS and NCS methods with similar resolution and flux parameters. Indeed, NRS measurements have been successfully performed on Vesuvio plus using samples containing Au, W, Ta, U, Mo, Nd, Pr^23,94–97^ and other nuclei with resonant neutron absorption in the epithermal neutron energy region. Conversely, one can imagine performing NCS experiments characterised by similar resolution and flux parameters as their Vesuvio plus counterparts using compact neutron sources and moderators already shown to be able to realise NRS experiments.

There is considerable scope for further improving the thermal neutron flux and shortening neutron pulse duration from compact neutron sources by optimising the moderator design, as well as the fast input neutron source beyond the limits of the sources presented in the work by Mirfayzi et al. Fast neutron fluxes of the order of 10$^{10}$ n/sr, which can be further improved by capitalising on advances in laser technology, are already available. Moreover, laser-driven neutron sources produce neutron pulses whose duration is defined solely by the moderator design, which should enable placing samples sub-metre distances from the moderators, a feature extremely important for the construction of remotely operated outdoor (or submerged) drones for illicit and/or hazardous substance detection^81,92^. To this end, Lee et al.^88^ designed an epithermal neutron moderator at ambient temperature for short-distance TOF measurements using assemblies with compact laser-driven neutron sources. The design was based on the empirical relationships between the neutron intensity and energy resolution of epithermal neutrons and moderator size obtained from Monte Carlo simulation. The authors came up with an interesting design of a hollow cylinder moderator placed around the target source composed of a main moderator made out of poly-ethylene, a top moderator, and a backside reflector, both composed of Berylium or Tungsten. The total size of the moderator should not be more than 3 cm in order to achieve the energy resolution of 0.008 at a neutron flight distance of less than 5 m^88^.

Before we proceed to the discussion of how the LOD and LOQ values obtained for melamine compare to those obtained using other techniques, we have to understand why one can compare the values of the LOD and LOQ of these two techniques. As described in the Methods section, fitting the NCS spectra can be more accurate by applying the stoichiometric fixing technique, whereby the ratios of the integral recoil peak areas are fixed using the known sample isotopic composition. In detecting hazardous materials, one always knows beforehand the type of the substance sought. Thus, a given amount of the total scattering intensity of nitrogen, obtained from the fitting procedure of the backscattering NCS data, subject to the stoichiometric constraint reflecting the isotopic composition of melamine, is equivalent to detecting the melamine itself. In the neutron transmission technique, the asymptotic part of the transmission curve, corresponding to the incident energies of epithermal neutrons, is also directly proportional to the sample stoichiometry, but the proportionality constants are, unlike in the case of the NCS, the total free scattering cross-section values.

In this work, using the NT technique, we have obtained the LOD value of 0.16 grams (1269 $\mu$ mol) and the LOQ value of 0.52 grams (3807 $\mu$ mol) for the detection of melamine. In the case of the NCS technique, the LOQ and LOQ values are 0.027 grams (214 $\mu$ mol) and 0.092 grams (642 $\mu$ mol), respectively. These values are much higher than the LOD values obtained using other existing techniques of melamine detection as a contaminant in food, wastewater and other liquid or semi-liquid media^98^. For this purpose, techniques such as gas chromatography/mass spectrometry (GC/MS), enzyme-linked immunosorbent assay (ELISA), terahertz time-domain spectroscopy, surface-enhanced Raman scattering (SERS), UV-SERS and electrochemical sensing methods are widely applied^99^. In the case of the measurements of LODs of melamine in solid-state systems, methods such as solid phase extraction^100^ and mass-spectrometric techniques such as Direct Analysis in Real Time (DART) are frequently applied^101^. An interesting method for indirect melamine detection was presented as a by-product of the absolute determination of the neutron source yield^102^. With this method, the amounts of melamine as low as 10 mg could be detected. However, the major drawback of this methodology is that the sample preparation for the analysis consists of direct combustion of melamine and graphitisation of obtained CO$_2$^102^, which cannot be performed in situ in a natural environment setting. Moreover, in order to achieve the required melamine LOD of 10 mg, the irradiation of the melamine sample had to be performed continuously for the period of four months^102^, which is a prohibitively long period of time for the environmental detection. Melamine plays an important role as a surrogate substance in the context of neutron-based explosive detection. To this end, there is ongoing research and development of advanced humanitarian landmine detection systems using a compact discharge-type fusion neutron source called IECF (Inertial-Electrostatic Confinement fusion) devices ^103^. IECF-based setup would be able to detect sub-kilogram (800 g) amounts of melamine^103^. An interesting alternative to this protocol was proposed based on $\gamma$–$\gamma$ coincidence measurements to detect weak transitions in deuterated melamine^104,105^. With this method, melamine detection limits are in the order of 100 grams^104^. The tagged neutron method (TNM) has also been applied for detecting and identifying explosive materials^105,106^, with samples of melamine of masses of ca. 4 kg successfully detected^106^.

Apart from the fact that many experimental protocols involving the above methods require transparent media, many of the techniques mentioned above are designed to operate in controlled environments, where backgrounds coming from other substances or solvents produce vibrational responses well separated in frequency and/or magnitude from the characteristic vibrational peaks of the melamine. Thus, in the natural environment, poor signal-to-background ratios may hinder successful melamine detection, and one has to resort to more robust methodologies, such as those based on direct or indirect detection of the interaction of neutrons with matter. To this end, an interesting method for indirect melamine detection was presented as a by-product of the absolute determination of the neutron source yield^102^. Melamine has the ability to detect neutrons via $^{14}$N(n, p)$^{14}$C reaction and subsequent determination of $^{14}$C content. A cross-section for this reaction is relatively high for thermal neutrons (1.827 barns) and much lower for fast neutrons. A concentration of $^{14}$C nuclei created in the irradiated sample of melamine can be reliably measured with the aid of accelerator mass spectrometry (AMS). The mass of melamine sufficient for this analysis was determined to be only 10 mg^102^. However, the major drawback of this methodology is that the sample preparation for the AMS analysis consists of direct combustion of melamine and graphitisation of obtained CO$_2$^102^, which cannot be performed in situ in a natural environment setting. Moreover, in order to achieve the required melamine LOD of 10 mg, the irradiation of the melamine sample had to be performed continuously for the period of 4 months^102^, which is a prohibitively long period of time for the environmental detection.

Melamine plays an important role as a surrogate substance in the context of neutron-based explosive detection. To this end, there is ongoing research and development of advanced humanitarian landmine detection systems using a compact discharge-type fusion neutron source called IECF (Inertial-Electrostatic Confinement fusion) devices^103^. In this setup, 10.8 MeV $\gamma$-rays produced through $(n,\gamma )$ reaction with nitrogen atoms in the melamine powder were demonstrated to be able to be measured by a BGO(bismuth-germanium-oxide-sodium iodide)-combined scintillation sensor^103^. This setup works in realistic conditions for landmines buried relatively deeply in the soil and in the presence of $\gamma$-rays emitted by the soil elements, for example, Si^103^. The values of the LOD and LOQ of melamine detected in this experimental protocol were not given in the original work, however, and the only hint was provided that the IECF-based setup would be able to detect sub-kilogram (800g) amounts of melamine^103^. An interesting alternative to this protocol was proposed based on $\gamma$–$\gamma$ coincidence measurements to detect weak transitions in deuterated melamine^104^. Two 90 high-purity germanium (HPGe) $\gamma$ detectors were set 2 cm from the deuterated melamine target, and the distance between them was 4 cm. In this work, the deuterated melamine powder was compressed into a tablet of 20 mm diameter and 5 mm thickness, which would imply that the LODs for this particular experimental protocol would be in the region of 6 grams (assuming the packing density of one gram per cubic centimetre). Further improvement with respect to the protocol published in Ref.^104^ involved simultaneous measurements using anticoincidence and coincidence methods and a combination of BGO and NaI(Tl) scintillators, resulting in lower (in the order 100 gram) melamine detection limits^107^.

An interesting turn in the history of the development of (n,$\gamma$) reaction-based nitrogen in explosives detection methods is due to the work of Nasrabadi et al.^105^. The authors developed a protocol whereby only the ratios of the net full-energy peak areas of nitrogen and hydrogen peaks in the $\gamma$ spectra from irradiated melamine were used to identify and quantify the substance. Using relative instead of absolute peak areas simplifies the analysis and renders it much more robust as it becomes independent of the technique and spectrometer-specific data acquisition parameters^105^. In its spirit, this technique is reminiscent of the stoichiometric fixing technique used in this work to treat the NCS spectra. In both approaches, the relative peak areas are proportional to the relative amounts of nitrogen and hydrogen in the melamine sample, and in both cases, the peak area ratios can be compared between the experiment and the simulations. Conducting this benchmarking procedure as a function of the decreasing mass of the sample allowed Nasrabadi et al. to approach the limit of detection of melamine for the samples of mass of 100 grams^105^.

Following this spirit of neutron spectroscopic data analysis, Bishnoi et al. conducted a feasibility study of applying the tagged neutron method (TNM) for detecting and identifying explosive materials^106^. In a D-T (14.1 MeV) neutron source, each neutron is emitted almost back-to-back with an alpha particle, whose detection by a position-sensitive detector determines the direction of the outgoing neutrons. Thus, neutrons are tagged by time and direction. Neutron-induced $\gamma$ rays are detected in coincidence with alpha particles, which allows the construction of a neutron time-of-flight spectrum. TOF determines the position of neutron interaction along the tagged neutron path, and spectroscopic analysis of the $\gamma$-ray spectrum associated with a given volume provides information about the chemical elements located in it^105^. The geometrical configuration includes 14.1 MeV-tagged neutrons, a position-sensitive alpha detector and an array of BGO $\gamma$ detectors arranged in a square geometry around the sample to collect the neutron-induced $\gamma$-ray spectra^106^. Simulation results have shown the system’s capability in detecting and imaging hidden explosives (of masses of the order of 1 kg) within metallic and wooden matrices^106^. In a follow-up study^108^, the authors provided a comprehensive characterization of the TNM setup by performing calibration measurements using objects were graphite, water, melamine and explosive simulant (RDX- C$_3$H$_6$N$_6$O$_6$). Sample size (3–10 kg) and the experimental geometry (distance and position of the sample) were chosen so that the tagged neutron beam intercepted the whole sample, and the $\gamma$ detector effectively collected the neutron-induced gammas from the sample. A 4 kg sample of melamine was irradiated for 20 min, and all its $\gamma$ spectral signatures were properly detected^106^.

On the whole, for studies mimicking environmental detection of nitrogen in explosives with neutrons, the values of the LOD of nitrogen in melamine reported in the literature (or those that could be inferred from the descriptions present in the literature) for different detection protocols are, in most cases, higher than the values obtained in this work using the NT and NCS techniques. Moreover, there is further room for improvement in the setup described in this work. Firstly, the concurrent use of the NCS, NT, and prompt $\gamma$ activation analysis is possible with portable high-purity germanium or high-resolution scintillating $\gamma$ detectors^4,8^. Work is currently being done to set up such a $\gamma$ detection protocol on the VESUVIO beamline. An adequately designed sample environment with a low neutron background for neutron powder diffraction detection in hazardous materials and their surrogates could augment the existing protocol by simultaneous structural refinement.

The *ab initio* modelling methodology of the NCS and NT spectra, described in the Methods section, could further improve the accuracy of the determination of the values of LOD and LOQ of the NCS and NT techniques. Namely, one could fix the values of the widths of the momentum distributions underlying the shapes of the recoil peaks of all constituent isotopic species and simultaneously apply the stoichiometric fixing of their relative intensities. Similarly, in treating the transmission curves, one could approximate the experimental data with synthetic curves obtained from DFT-based simulations. As much as DFT-augmented NCS and NT data approximation can be used in the future in hazardous material detection on an industrial scale to speed up and streamline the detection process, this study has merely used it to benchmark against the experimental data. Due to the presence of non-negligible nuclear quantum effects that can, in principle, only be exactly simulated using the path-integral molecular dynamics methods, the DFT methodology used in this work will always produce small systematic discrepancies with respect to the experimental data obtained from both the NCS and NT techniques. Thus, it is important to assess how well the harmonic lattice dynamics and *ab initio* molecular dynamics techniques approximate the local nuclear quantum dynamics of hydrogen, nitrogen and oxygen in melamine, with the aim of devising more accurate *ab initio* computational schemes for future research related to nuclear quantum effects in this important system.

From the computational perspective, our systematic study illustrates the importance of materials modelling towards understanding the microscopic structure and nuclear dynamics in molecular explosives, reflected in neutron scattering observables. A numerically accurate benchmark indicates room for further improvements. First, calling for future extension toward the use of hybrid density functional approximations in conjunction with more advanced vdW-correction methods, including MBD, D4, or XDM^109^. Second, intrinsic limitations of classically sampled MD simulations call for further improvement, making melamine a good candidate for benchmark studies using semi-quantum *ab initio* approaches invoking path integrals or anharmonic lattice dynamics^110–112^. A good description of both external lattice modes and anharmonic internal vibrations is of pivotal importance for understanding the mechanisms behind the detonation of molecular explosives^113^.

## Methods

### Sample preparation and sample environment

Melamine was purchased from Sigma Aldrich in a powder form of 99% purity (product number M2659) and used directly in this form in the NCS and NT experiments. The powder was loaded into flat cylindrical aluminium containers, routinely used for VESUVIO experiments. To determine the values of LOD and LOQ, different amounts of powder were loaded into the containers, and the space inside the containers was filled by placing flat aluminium disks. The diameters of the flat aluminium disks and the aluminium containers were matched to the average incident neutron beam diameter of 5 cm^93^. The containers were oriented perpendicular to the direction of the incident neutron beam. Five samples containing 0.290, 0.575, 1.152, 1.580, and 2.299 grams of melamine were prepared.

NCS experiments were performed at the VESUVIO spectrometer installed at the ISIS Pulsed Neutron & Muon Source^22,23,26,71,93,94,114,115^. The melamine powder samples were measured at 300 K, with the temperature values stabilised using a closed-circuit refrigerator (CCR) mounted inside the VESUVIO beamline. For each sample, data were collected for approximately 27h, corresponding to an integrated proton current within the ISIS synchrotron of 2400 $\mu$Ah.

### Neutron transmission

Neutron transmission was measured employing an established protocol described elsewhere^24,25,116–118^. As widely explained in the literature, VESUVIO is unique in the context of NT experiments as it allows for neutron transmission (total neutron cross-section) to be measured in an extensive range of incident neutron energies, *E*, from a fraction of a mili electron-volt to thousands of electron volts. The *E*-dependent sample transmission is defined as:

2 $$\begin{aligned} T_s(E) = \exp {\left( -S_s(E)\right) } \end{aligned}$$

where *S*(*E*) is the *E*-dependent sample scattering power.

In the presence of a sample container, characterised by the transmission curve given by $T_c(E)$, the total sample and container transmission is $T_s(E) T_c(E)$. Thus, the $T_s(E)$ curve can be obtained by the pointwise division of the measured sample and container transmission curve by the pre-measured transmission curve of an empty container.

The asymptotic (measured for values of *E* in the range of tens to hundreds of electron-volts) values of $S_s(E)$ (S$_{as}$) and $T_s(E)$ (T$_{as}$) are connected so as $S_{as} = 1 - T_{as}$. Owing to this exceptionally wide incident neutron energy range, the values of S$_{as}$ and T$_{as}$ can be very easily obtained directly from the experiment. The value of S$_{as}$ obtained directly from the VESUVIO experiment can then be compared with its theoretical counterpart given by:

3 $$\begin{aligned} S_{as} = d n \sum _{i=1}^{N} c_i \frac{4\pi b_i^2}{(1 + m/M_i)^2} \end{aligned}$$

where *d* is the sample thickness along the direction of the incident neutron beam, *n* is the sample number density, $c_i$ is the number of the i-th isotopic species per sample formula unit, $4\pi b_i^2$ is the value of the total bound neutron scattering cross-section (with $b_i^2$ being the value of the total bound scattering length) of the i-th isotopic species. It is worth noting that the expression $\frac{4\pi b_i^2}{(1 + m/M_i)^2}$ denotes the value of the total free scattering cross-section of the i-th isotopic species. Finally, *m* and $M_i$ are the neutron and isotopic species masses, both expressed in atomic mass units, and *N* is the total number of different types of isotopic species present in the sample under investigation.

Equation 3 is very useful in two experimental aspects. Firstly, it allows the planning of each NT and NCS experiment by adjusting the value of *d* for each sample (given the known values of *n* and sample composition as well as tabulated values of total neutron cross-sections) in order for the value of *S* not to exceed 10%, which ensures that the ratio of multiple neutron scattering (MS) in the sample to the total scattering is less than 1%, and thus the effects of the MS can be neglected during the analysis of NT data. Secondly, Eq. (3), in the absence of the exact values of *d* and *n*, allows for the calibration of the total neutron cross-section curve obtained from the experimental transmission curve by requiring that the asymptotic value of the scattering cross-section be equal to $\sum _{i=1}^{N} c_i \frac{4\pi b_i^2}{(1 + m/M_i)^2}$.

### Neutron Compton scattering

A detailed description of the raw NCS data treatment is described elsewhere^23,26,27,115,119–122^, and thus here only main points of the data analysis workflow will be briefly described.

The NCS spectra of C$_3$H$_6$N$_6$, recorded in the Time-Of-Flight (TOF) domain (hereinafter denoted as *t*), were treated as a superposition of recoil peaks attributed to hydrogen, carbon and nitrogen, as well as aluminium from the sample container and sample spacer material. The NMDs underlying the recoil peaks were fitted in the domain of the longitudinal momentum *y*(*t*) with longitudinal momentum distributions denoted as *J*(*y*(*t*)). For isotopic species other than hydrogen, the single-dimensional isotropic Gaussian distributions were used:

4 $$\begin{aligned} J(y_M(t))= \frac{1}{\sqrt{2\pi \sigma _M^2}}\exp {\left( -\frac{y_M(t)^{2}}{2\sigma _M^2}\right) } \end{aligned}$$

In the case of hydrogen, apart from the single-dimensional isotropic Gaussian distributions, powder-averaged multivariate (three-dimensional) Gaussian distributions were also fitted:

5 $$\begin{aligned} J(y_M(t))= \frac{1}{\sigma _{M,x}\sigma _{M,y}\sigma _{M,z}\sqrt{2\pi }}\frac{2}{\pi }\int _0^{1} d(\cos {(\theta )}) \int _0^{\pi /2} d\phi S^2(\theta ,\phi )\exp {\left( -\frac{y_M(t)^{2}}{2S^2(\theta ,\phi )}\right) } \end{aligned}$$

where

6 $$\begin{aligned} \frac{1}{S^2(\theta ,\phi )} = \frac{\sin ^2{\theta }\cos ^2{\phi }}{\sigma _{M,x}^2}+\frac{\sin ^2{\theta }\sin ^2{\phi }}{\sigma _{M,y}^2}+\frac{\cos ^2{\theta }}{\sigma _{M,z}^2} \end{aligned}$$

There is an associated value of the excess peak kurtosis, $c_{4,M}$, that can be computed based on the values of $\sigma _{M,x}$, $\sigma _{M,y}$, and $\sigma _{M,z}$^123^:

7 $$\begin{aligned} c_{4,M} = \frac{2/5[(\sigma _{M,x}^2- \sigma _{M,y}^2)^2+(\sigma _{M,z}^2- \sigma _{M,x}^2)^2+(\sigma _{M,y}^2- \sigma _{M,z}^2)^2]}{(\sigma _{M,x}^2+ \sigma _{M,y}^2+ \sigma _{M,z}^2)^2} \end{aligned}$$

In fitting the NCS spectra, the ratios of the relative integral intensities, $\frac{I_M}{I_M'}$ of the recoil peaks of isotopic species of masses *M* and $M'$ were linearly constrained according to the formula:

8 $$\begin{aligned} \frac{I_M}{I_M'} = \frac{4\pi b_M^2 A_M}{4\pi b_M'^2 A_M'} \end{aligned}$$

where $A_M$ and $A_M'$ are number of isotopes of type *M* and $M'$ respectively per formula unit of melamine. This technique, referred to as stoichiometric fixing, is widely used in fitting NCS data^122,124–127^. It is worth noting that, due to kinematic constraints, the NCS spectra recorded in backscattering do not contain recoil peaks of hydrogen. Thus, to increase the accuracy of the hydrogen recoil peak fitting, the data were first fitted sequentially (detector-by-detector) in backscattering, and the average values of the NMD widths of isotopic species other than hydrogen were calculated. The stoichiometric fixing was employed for all isotopic species, with the exception of aluminium, and the NMD width of aluminium was fixed at the value of 14 Å$^{-1}$ obtained from a series of calibration experiments, as described in^116^. Following this, the sequential fitting of the forward scattering data was performed using the values of the NMD widths of those masses fixed to the average values obtained from backscattering, and only the NMD width of the hydrogen was free in fitting.

Both the raw NCS data recorded in backscattering and forward scattering were subject to an iterative correction procedure to include the effects of the multiple neutron scattering in the sample as well as the sample composition-dependent $\gamma$ background, according to the procedures described in the literature^23,26,27,115,119–122^.

### *Ab initio* modelling

#### *Ab initio* modelling of NCS observables

*Ab initio* Modelling of NCS observables was performed in order to reproduce experimental results recorded at room temperature. The electronic structure calculations were performed under Periodic Boundary Conditions (PBCs). The calculation proceeded in two steps. First, the geometry optimization was performed. Subsequently, the vibrational responses were calculated using two different approaches. In the first approach, HLD calculation was performed, and the phonon dispersion, as well as the total (VDoSs) and partial (atom-projected) vibrational density of states (apVDoSs), were calculated. In the second approach, *ab initio* Molecular Dynamics (AIMD) simulation following the Born-Oppenheimer approximation (BOMD) was performed at T = 300 K.

In the context of the NQEs and as a matter of disambiguation, it is worth noting that both the HLD and AIMD should be referred to as ’classical’ computational schemes, with the term ’classical’ signifying that throughout the entire HLD and AIMD computational protocols, nuclei are treated as classical (i.e. point-like) particles^69^. Thus, the contribution of phenomena such as nuclear quantum delocalisation (NQD) or tunnelling (NQT) to the values of the NMD widths cannot be directly captured by these simulations, and additional considerations are necessary when comparing experimental and simulated values of NCS observables^71^.

The initial model was based on the low-temperature structure of melamine, solved from the neutron diffraction (ND) data at 14 K by Cousson et al.^40^. CASTEP code (version 22.1)^128,129^, employing the Plane-Wave Pseudo-Potential (PW-PP) formulation of Density Functional Theory (DFT). The Perdew-Burke-Ernzerhof (PBE) functional within the Generalised-Gradient-Approximation (GGA) augmented with semi-empirical dispersion corrections was used throughout the entire calculation. A set of on-the-fly-generated norm-conserving PPs described the core electrons. To build a credible reference for further investigation, we employed strict numerical conditions as developed in our previous works on similar molecular systems^130–135.^ The electronic wave functions were computed using an extended PW basis set with a kinetic energy cutoff of 1200 eV to ensure a proper description of intermolecular forces^134–137^. The convergence of the Monkhorst-Pack grid was tested within the *k*-point spacing interval of 0.07–0.02 Å$^{-1}$, finding 0.05 Å$^{-1}$ sufficient for well-converged phonon properties. The phonon band structure was used for thermophysical predictions according to Ref.^138^ The analysis of the vibrational modes was performed utilizing PDIelec code^139^. Simulations of the INS spectra were performed with the help of oCLIMAX^61^. Variable-cell relaxation of residual isotopic forces was performed under atmospheric pressure, imposing a three-point finite basis set correction. The convergence criteria in the self-consistent field (SCF) variation were set to 1.0$\times$^-12^ eV/atom. During the geometry optimization, the convergence threshold values for the maximum energy change in eV/atom, the maximum force in eV/Å, the maximum stress in GPa, and the maximum displacement in Å were set to 1.0$\times$e$^{-10}$, 1.0$\times$e$^{-5}$, 1.0$\times$e$^{-3}$, and 1.0$\times$e$^{-5}$, respectively.

The formulation of the Density Functional Perturbation Theory (DFPT) in the reciprocal space^23,140–142^ was employed in the limit of T = 0 K to calculate phonon-dispersion relations across the 1BZ. The dispersion relations were obtained by numerically mapping the dependence of phonon eigenvectors and eigenfrequencies on the momentum transfer by solving the eigenvalue problem of the Dynamical-Matrix (DM) as a function of the momentum transfer, where the DM is defined as^23,143^:

9 $$\begin{aligned} D_{\mu \nu }(q) = \frac{1}{N_R} \sum _{R, R'} D_{\mu \nu }(R-R') \exp (-iq(R-R')) \end{aligned}$$

where *q* is the momentum transfer, $N_R$ the number of Bravais lattice sites, *R* and $R'$ are isotopic equilibrium positions within the 1BZ and $D_{\mu \nu }(R-R')$ is the force constant matrix (FCM) defined as^23,143^:

10 $$\begin{aligned} D_{\mu \nu }(R-R') = \frac{\partial ^2 E}{\partial u_{\mu }(R)\partial u_{\nu }(R')}|_{u=0} \end{aligned}$$

where *u* is the displacement of a given atom from its equilibrium position and *E* is the total energy within the harmonic approximation.

Following the commonly adopted assumption that the nuclear momentum distribution (NMD) of isotopic species of mass *M* in a crystal along the $\widehat{q}$ direction assumes a purely multivariate Gaussian functional form, the second-moment of the NMD can be written as^23,143^:

11 $$\begin{aligned} \sigma _{M,HLD}(\widehat{q})^2 = \frac{M}{N_q \hbar ^2} \sum _{q\in 1BZ} \sum _{\lambda =1}^{N_{\lambda }}\left( e(\lambda ,q)\cdot \widehat{q}\right) ^2\frac{\omega (\lambda ,q)}{2}\coth \left( \frac{\omega (\lambda ,q)}{2k_BT}\right) , \end{aligned}$$

where $\omega (\lambda ,q)$ are phonon frequencies and $e(\lambda ,q)$ are phonon eigenvectors and the summation runs over all *q* vectors contained in the 1BZ $N_q$ and all phonon branches (q-dependent modes) $\lambda$. The expression $\coth \left( \frac{\hbar \omega }{2k_BT}\right)$ is the Boltzmann factor, which represents the temperature-dependent population of vibrational levels of the quantum harmonic oscillator accounting for the temperature effect, and $k_B$ is the Boltzmann constant.

The spherically averaged value of $\sigma _{M,HLD}^2$ can be obtained using two different computational routes^23,143^. The first route uses the following expression:

12 $$\begin{aligned} \sigma _{M,HLD}^2 =\frac{1}{3}(\sigma _{M,HLD}(\widehat{x})^2 +\sigma _{M,HLD}(\widehat{y})^2+ \sigma _{M,HLD}(\widehat{z})^2) \end{aligned}$$

The multivariate Gaussian model of the NMD, apart from being directly linked to the phonon dispersion, allows for a detailed analysis of the contributions to the NMD of a given nuclear species from individual modes of vibration in a crystal. Namely, for a given nucleus of mass *M*, one can define the *q*-averaged contribution of a given mode, $\lambda$, to the nuclear kinetic energy of this nucleus along the *x*, *y*, *z* directions:

13 $$\begin{aligned} \frac{\sigma _{M,\lambda ,HLD}(\widehat{x},\widehat{y},\widehat{z})^2}{\sigma _{M,HLD}(\widehat{x},\widehat{y},\widehat{z})^2} = \frac{\sum _{q\in 1BZ} \left( e_{(\widehat{x},\widehat{y},\widehat{z})}(\lambda ,q)\right) ^2\frac{\omega (\lambda ,q)}{2}\coth \left( \frac{\omega (\lambda ,q)}{2k_BT}\right) }{\sum _{q\in 1BZ} \sum _{\lambda =1}^{N_{\lambda }}\left( e_{(\widehat{x},\widehat{y},\widehat{z})}(\lambda ,q)\right) ^2\frac{\omega (\lambda ,q)}{2}\coth \left( \frac{\omega (\lambda ,q)}{2k_BT}\right) } \end{aligned}$$

where $$e_{(\widehat{x},\widehat{y},\widehat{z})}(\lambda ,q) = e(\lambda ,q)\cdot (\widehat{x},\widehat{y},\widehat{z})$$.

Alternatively, one can define the *q*-averaged contribution of a given mode, $\lambda$, to the total nuclear kinetic energy of this nucleus, summed over the *x*, *y*, *z* directions:

14 $$\begin{aligned} \frac{\sigma _{M,\lambda ,HLD}(\widehat{x})^2 +\sigma _{M,\lambda ,HLD}(\widehat{y})^2+ \sigma _{M,\lambda ,HLD}(\widehat{z})^2}{3\sigma _{M,HLD}^2} = \frac{\sum _{q\in 1BZ} \sum _{(\widehat{x},\widehat{y},\widehat{z})} \left( e_{(\widehat{x},\widehat{y},\widehat{z})}(\lambda ,q)\right) ^2\frac{\omega (\lambda ,q)}{2}\coth \left( \frac{\omega (\lambda ,q)}{2k_BT}\right) }{\sigma _{M,HLD}(\widehat{x})^2 +\sigma _{M,HLD}(\widehat{y})^2+ \sigma _{M,HLD}(\widehat{z})^2} \end{aligned}$$

In the second route to compute the average NMD variance, one employs the atom-projected (partial) vibrational density of states (apVDoSs), $G_{M,HLD}(\omega )$, using the relation:

15 $$\begin{aligned} \sigma _{M,HLD}^2 = \frac{M}{\hbar ^2} \int G_{M,HLD}(\omega )\frac{\omega }{2}\coth \left( \frac{\omega }{2k_BT} \right) d\omega , \end{aligned}$$

where $G_{M,HLD}(\omega )$ is defined as^23,143^:

16 $$\begin{aligned} G_{M,HLD}(\omega ) = \frac{1}{3 N_q} \sum _{q\in 1BZ} \sum _{\lambda =1}^{N_{\lambda }} e(\lambda ,q)^2 \delta (\omega -\omega (\lambda ,q)) \end{aligned}$$

The second independent approach to accounting for vibrational properties of systems under investigation was to employ classical AIMD simulations following the Born–Oppenheimer MD scheme at T = 300 K. The unit cell of melamine was used with the *k*-point sampling specification identical to that used in the HLD simulation (*k*-point spacing of 0.02 Å$^{-1}$ and *k*-point grid of the size of 5$\times$7$\times$8). The other parameters associated with the electronic Hamiltonian, including the basis set and pseudopotentials, were kept at the values used for the HLD simulations, apart from the SCF convergence, which has been reduced to $\times$ 1$\times$e^−^ eV/atom, and the fine-grid multiplier was reduced to the FFT size. Each initial supercell was equilibrated for 5 ps under atmospheric pressure in the isothermal-isobaric ensemble (NPT). Additional tests were performed in the NPT ensemble to test the influence of *k*-point sampling and the second-grid definition to resulting volumetric properties. The influence was found to be negligible. The Equations Of Motion (EOMs) were solved every 0.5 fs. The isotropic Andersen barostat and a classical Nose-Hoover thermostat were used at a given temperature. The entire set of supercells obtained from all time steps of the NPT simulation was collected, and the average supercell was calculated. A 5 ps equilibration was performed in the canonical ensemble (NVT) with the time step of 0.5 fs using this average supercell. Finally, the production run of 25 ps was completed in the microcanonical ensemble (NVE), also using the time step of 0.5 fs. The NVE trajectories were used to model the apVDoSs. The apVDoS for a particular atom *M* was calculated as the Fourier-transform of its Velocity Auto Correlation Function (VACF):

17 $$\begin{aligned} G_{M,AIMD}(\omega , T)=\sum _{j}^{N}\int _{0}^{\infty }\frac{<\nu (t)_{j}\nu (0)_{j}>}{<\nu (0)_{j}^{2}>}exp\left( -i\omega t \right) dt, \end{aligned}$$

where $\nu (t)_{j}$ is the velocity at time $\textit{t}$.

The apVDoSs of individual isotopic species, obtained from the AIMD simulation scheme, were then used to compute the values of the second moments NMDs using the expression analogous to Eq. 15. However, in this case, the temperature dependence that, in the case of the HLD simulation, was accounted for by the Boltzmann factor is already incorporated in the AIMD trajectories, and thus, the expression is modified to:

18 $$\begin{aligned} \sigma _{M,AIMD}^2 = \frac{M}{\hbar ^2} \int G_{M,AIMD}(\omega )\frac{\omega }{2} d\omega , \end{aligned}$$

The analysis and the post-processing of the BOMD production runs were made with the help of the TRAVIS code^144,145^. The apVDoSs were further used for calculating the values of NMD widths, $\sigma _M$, for a direct comparison with the values of the NMDs widths obtained from the HLD calculations and directly from the NCS experiments, following the methodology widely adopted in the NCS work on solid-state systems ^69,146,147^.

The value of the nuclear kinetic energy associated with the NMD width along the direction in space, given by $\widehat{q}$, can be written as:

19 $$\begin{aligned} E_{k,M}(\widehat{q})= \frac{\hbar ^2\sigma _M(\widehat{q})^2}{2M} \end{aligned}$$

The average nuclear kinetic energy, $E_k$, of an isotopic species of mass *M* can be obtained using the value of the second moment of its NMD using the following formula:

20 $$\begin{aligned} E_{k,M}= \frac{3\hbar ^2\sigma _M^2}{2M} \end{aligned}$$

where $\sigma _M$ represents the average value of the NMD width obtained either through the HLD computational route (using Eqs. (12 or  15)) or the AIMD computational route, using Eq. 18.

Moreover, by virtue of the virial theorem, the value of the zero-point vibrational energy ($ZPE_M$) can, to a very good degree of accuracy, and even for considerably anharmonic local effective potentials, be expressed as twice the value of the average nuclear kinetic energy, $ZPE_M = 2 E_{k,M}$^26^.

The variances of the NMD widths can also be used to calculate the magnitudes of the effective force constants acting on nuclei in their local environments, employing the theory of the mean force function (MF)^74^. The MF is defined as the average force acting on an isotopic particle by keeping all other particles in the system fixed^74^. The force-constant magnitude, $k_M$, for a nucleus of mass *M*, at temperature, *T*, characterised by the NMD width, $\sigma _M = \sigma _{M,HLD}, \sigma _{M,AIMD}, \sigma _{M,HLD}(\widehat{q})$, can be calculated using the formula^74^:

21 $$\begin{aligned} k_M = \frac{(k_BT)^2M}{\hbar ^2} - k_BT \sigma _M^2 \end{aligned}$$

#### *Ab initio* modelling of the total neutron cross-sections

Simulations of the total neutron cross-section as a function of the incident neutron energy in the range between 0.1 meV and 10 followed the protocols already established in the literature^24,25,116^ and relied on the modelling of the underlying apVDoSs in the incoherent approximation. In this approximation, the total (summed over all isotopic species) cross-section curves are expressed as sums of the total incoherent scattering cross-sections and absorption cross-sections. The incoherent scattering cross-sections of individual isotopic species were calculated from the underlying modelled apVDoSs. In the case of the AFGA method^24,25^, the apVDoSs of protons were calculated as linear combinations of proton apVDoSs of functional groups present in the samples under investigation that were pre-computed using *ab initio* methodology. For isotopic species other than hydrogen, the AFGA used the Debye approximation to compute the apVDoSs. In the other computational route, all isotopic species present in the samples were treated on equal footing, and their apVDoSs were computed from Eq. (16) using the HLD approach. For both computational routes, the NCRYSTAL software (version 2.7.3) was employed^116–118,148^. The simulated neutron absorption cross-sections were added to the simulated scattering cross-sections for comparison with experimental data. Nuclear resonances were not considered in the calculation of the absorption cross-sections as they are not observed from H, O, C, and N isotopic species for incident neutron energy values between 0.1 meV and 10 eV.

### Supplementary Information

Supplementary Information.

## Data Availability

The datasets used and/or analysed during the current study are available from the corresponding author upon reasonable request.
