# Supplementary material for: Non-invasive detection of hazardous materials with a thermal-to-epithermal neutron station: a feasibility study towards practical application
Source: Sci Rep. 2024 Aug 10;14:18584. doi: 10.1038/s41598-024-69290-x (PMC11316788; doi:10.1038/s41598-024-69290-x)
Supplement: Supplementary file 1 — Supplementary Information. [file 41598_2024_69290_MOESM1_ESM.pdf]

Supplementary Information for Publication: Non-invasive detection of hazardous materials with thermal-to-epithermal neutron station: a feasibility study towards practical application

Michał Silarski[1,+], Katarzyna Dziedzic-Kocurek[1,+], Kacper Drużbicki[2,+], Radosław Reterski[3], Patryk Grabowski[1], and Matthew Krzystyniak[4,*,+]

[1]M. Smoluchowski Institute of Physics of the Jagiellonian University, Łojasiewicza 11, 30-348 Cracow, Poland
[2]Centre of Molecular and Macromolecular Studies, Polish Academy of Sciences, Sienkiewicza 112, 90-363 Lodz, Poland
[3]Faculty of Chemistry, Jagiellonian University, Gronostajowa 2, 30-387 Cracow, Poland
[4]ISIS Neutron and Muon Source, STFC Rutherford Appleton Laboratory, OX11 0QX, United Kingdom
[*]matthew.krzystyniak@stfc.ac.uk
[+]these authors contributed equally to this work

Contents:

Additional computational results: *in silico* lattice dynamics of melamine

S1. Performance of dispersion-corrected density functional approximations

Figure S1. Vibrational density of states according to different density functional approximations

S2. Analysis of the zone-centre phonon modes

Table S1. Eigenergies and symmetry of the zone-centre modes
Figure S2. Internal-external vibrational energy composition
Figure S3. Nuclear composition of vibrational energy
Table S2. Assignment of the inelastic neutron scattering spectrum of melamine
Figures S4–S15. Schematic vibration patterns of the zone-centre phonon modes

S3. Phonon properties beyond the Γ–point

Figure S16. Phonon dispersion relations according to harmonic lattice dynamics
Table S3. List of the q-points defining the first Brillouin Zone
Table S4–S5. List of the phonon modes used for calculations of the neutron Compton scattering observables
Figure S17. Atom-projected vibrational density of states
Figure S18. Q-averaged individual mode contributions to the total kinetic energy of the nuclei for each element
Figure S19. Spatial decomposition of the q-averaged individual mode contributions to the nuclear kinetic energy

S1. Performance of dispersion-corrected density functional approximations

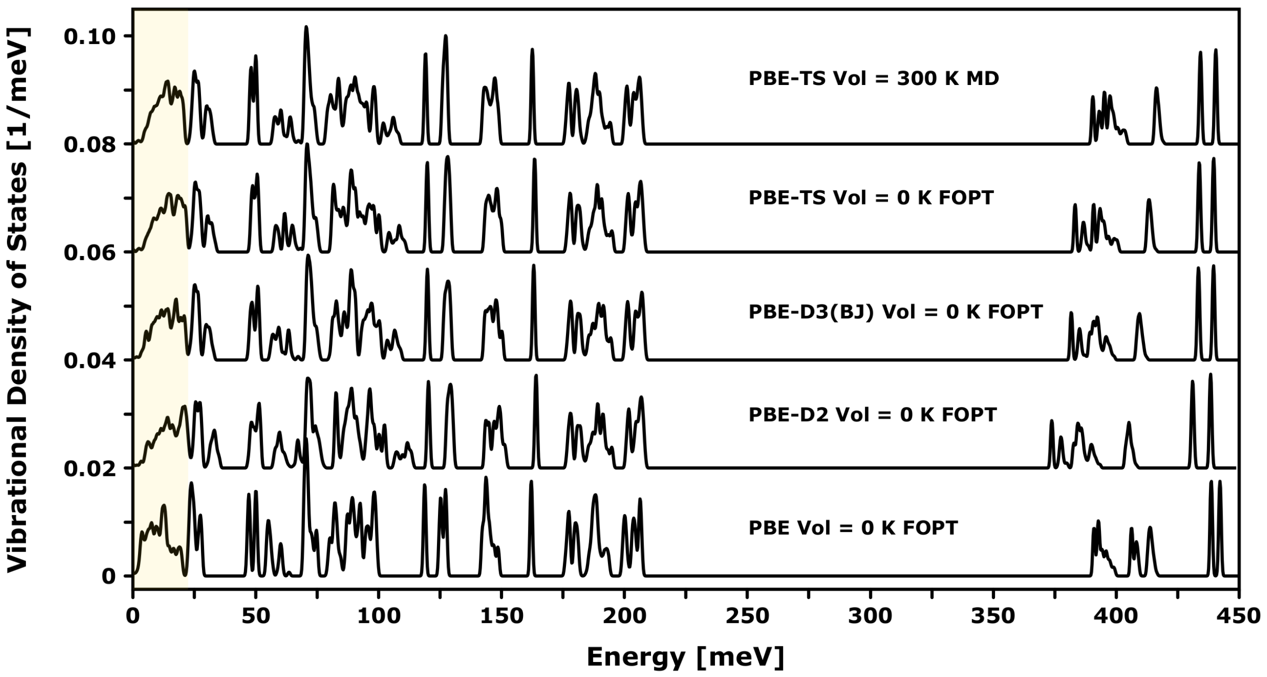

Figure S1. Theoretical vibrational density of states (VDoS) for the $P2_1/a$ phase of melamine according to different density functional approximations. The shaded regime highlights the external phonon modes. The cell volume was either fully optimised at 0 K (FOPT) or derived from AIMD simulations at 300 K (MD).

S2. Analysis of the zone-centre phonon modes

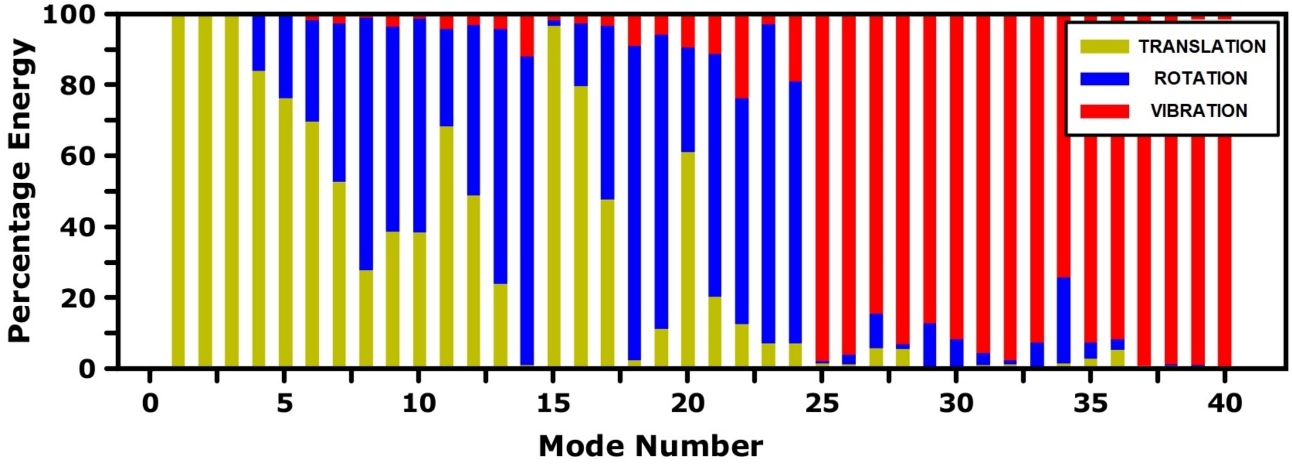

Figure S2. Vibrational energy decomposition of the zone-centre modes for the $P2_1/a$ phase of melamine according to harmonic lattice dynamics (linear response using PBE-TS functional). The contributions from external modes are further decomposed into translation and rotation of the molecular centroids (presented as yellow and blue bars, respectively). The contributions from internal modes are shown as red bars.

No.	meV [$\Gamma_{Symm.}$]	No.	meV [$\Gamma_{Symm.}$]	No.	meV [$\Gamma_{Symm.}$]	No.	meV [$\Gamma_{Symm.}$]	No.	meV [$\Gamma_{Symm.}$]
1	0.0 [Au]	37	48.1 [Ag]	73	88.3 [Ag]	109	143.8 [Au]	145	200.4 [Au]
2	0.0 [Bu]	38	48.3 [Bg]	74	88.7 [Bg]	110	144.0 [Bu]	146	200.5 [Bu]
3	0.0 [Bu]	39	49.4 [Au]	75	88.8 [Ag]	111	144.8 [Ag]	147	201.7 [Ag]
4	8.8 [Ag]	40	49.6 [Bu]	76	89.1 [Au]	112	145.0 [Bg]	148	201.8 [Bg]
5	9.5 [Bu]	41	50.2 [Ag]	77	89.3 [Bu]	113	145.2 [Au]	149	204.3 [Bg]
6	9.9 [Au]	42	50.3 [Bg]	78	89.8 [Bg]	114	145.8 [Ag]	150	205.1 [Au]
7	10.7 [Bg]	43	50.7 [Au]	79	90.4 [Bu]	115	146.4 [Bu]	151	205.3 [Bu]
8	11.1 [Au]	44	50.8 [Bu]	80	90.6 [Au]	116	147.5 [Au]	152	205.4 [Ag]
9	12.9 [Au]	45	58.3 [Au]	81	91.6 [Bg]	117	148.0 [Bg]	153	206.2 [Ag]
10	13.3 [Bg]	46	59.2 [Bu]	82	92.5 [Ag]	118	148.2 [Ag]	154	206.9 [Bu]
11	13.6 [Ag]	47	61.2 [Ag]	83	92.9 [Bu]	119	149.7 [Bg]	155	207.1 [Bg]
12	14.1 [Au]	48	62.7 [Au]	84	94.2 [Au]	120	150.0 [Bu]	156	207.6 [Au]
13	14.8 [Ag]	49	64.1 [Bg]	85	94.4 [Bg]	121	163.3 [Ag]	157	383.2 [Ag]
14	15.4 [Bu]	50	68.8 [Bu]	86	95.6 [Au]	122	163.3 [Bg]	158	383.4 [Bg]
15	15.6 [Bg]	51	69.8 [Ag]	87	97.3 [Ag]	123	163.5 [Bu]	159	386.6 [Au]
16	17.0 [Bg]	52	70.3 [Bg]	88	97.3 [Bu]	124	163.6 [Au]	160	388.8 [Bu]
17	17.9 [Ag]	53	70.6 [Au]	89	98.3 [Bg]	125	177.1 [Au]	161	390.8 [Bg]
18	18.3 [Bu]	54	70.6 [Ag]	90	98.4 [Ag]	126	177.3 [Bu]	162	390.9 [Ag]
19	19.0 [Au]	55	70.9 [Bg]	91	99.1 [Bu]	127	177.5 [Bg]	163	392.4 [Bu]
20	19.5 [Ag]	56	71.3 [Bu]	92	100.4 [Au]	128	177.7 [Ag]	164	393.4 [Au]
21	19.9 [Bu]	57	71.4 [Au]	93	105.1 [Au]	129	180.2 [Ag]	165	393.8 [Ag]
22	21.2 [Bg]	58	72.3 [Bg]	94	106.3 [Bu]	130	180.3 [Bg]	166	395.8 [Au]
23	21.3 [Bg]	59	72.5 [Ag]	95	107.6 [Ag]	131	181.3 [Au]	167	398.6 [Bg]
24	21.8 [Ag]	60	72.5 [Bu]	96	108.2 [Bg]	132	182.0 [Bu]	168	400.8 [Bu]
25	24.1 [Au]	61	74.7 [Bu]	97	119.4 [Ag]	133	186.1 [Ag]	169	412.6 [Ag]
26	25.0 [Au]	62	75.4 [Au]	98	119.6 [Au]	134	186.4 [Bu]	170	412.7 [Au]
27	25.1 [Bg]	63	80.9 [Au]	99	119.6 [Bg]	135	187.2 [Bg]	171	413.1 [Bg]
28	25.6 [Ag]	64	81.1 [Bg]	100	120.0 [Bu]	136	187.9 [Au]	172	413.3 [Bu]
29	25.9 [Bu]	65	81.5 [Ag]	101	126.9 [Au]	137	188.5 [Au]	173	433.6 [Ag]
30	26.8 [Bu]	66	83.3 [Bg]	102	127.2 [Ag]	138	189.0 [Ag]	174	433.7 [Bg]
31	27.5 [Bg]	67	83.7 [Ag]	103	127.5 [Bg]	139	190.6 [Bu]	175	433.9 [Au]
32	27.7 [Ag]	68	83.9 [Bu]	104	127.5 [Bu]	140	190.8 [Bg]	176	434.1 [Bu]
33	29.9 [Ag]	69	84.5 [Au]	105	128.0 [Bu]	141	190.9 [Bg]	177	439.5 [Ag]
34	30.1 [Bu]	70	85.3 [Ag]	106	128.1 [Bg]	142	191.8 [Ag]	178	439.6 [Au]
35	31.2 [Au]	71	86.8 [Bu]	107	128.5 [Ag]	143	192.6 [Au]	179	439.6 [Bu]
36	31.8 [Bg]	72	88.2 [Bg]	108	128.8 [Au]	144	194.1 [Bu]	180	439.6 [Bg]

Table S1. The full set of the Γ–point vibrations of melamine crystal ($P2_1/a$). The phonon eigenenergies (in meV) were obtained from harmonic linear-response calculations using PBE-TS functional. The phonon symmetries are given in square brackets.

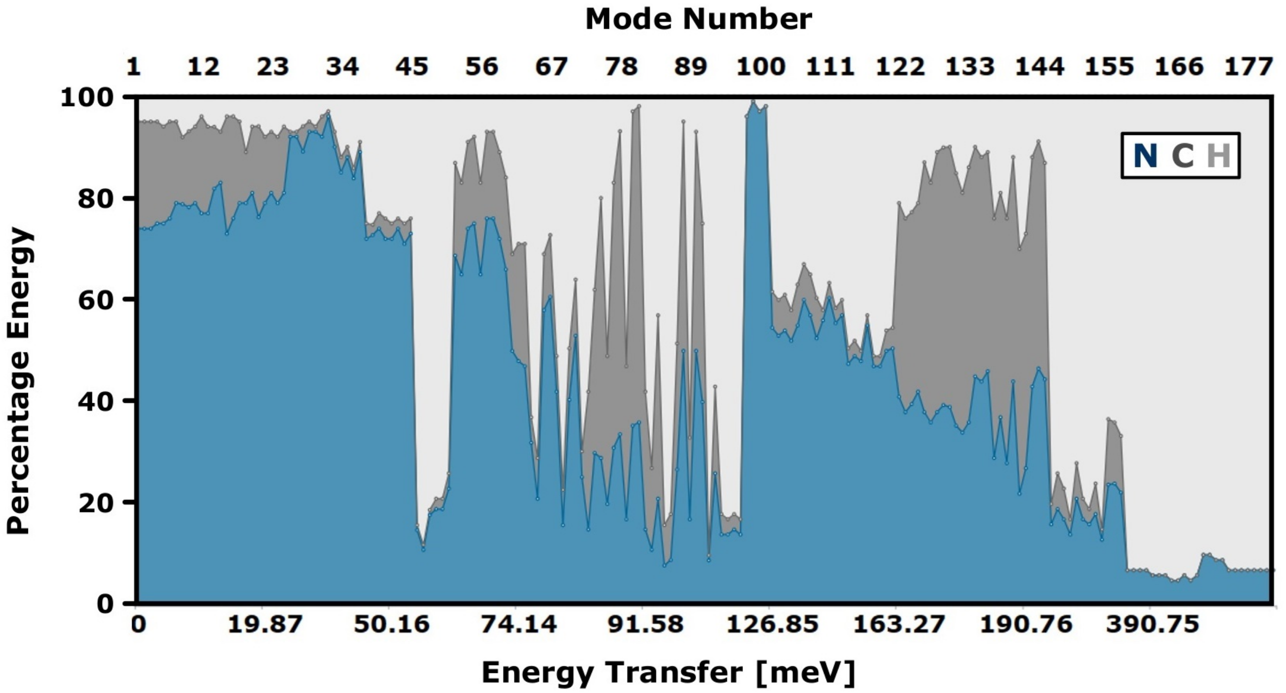

Figure S3. Percentage nuclear contributions to the zone-centre modes in the melamine crystal ($P2_1/a$) according to harmonic linear-response calculations with PBE-TS.

ID	INS	HLD	MD	Assignment	ID	INS	HLD	MD	Assignment
i	404.7	392.4 (No. 163)	398.5	ν_s(H-N-H)	*xii*	84.1	83.9 (No. 68)	79.8	R6–ring
ii	204.3	206.2 (No. 153)	203.7	δ(H-N-H)	*xiii*	79.6	81.1 (No. 64)	79.6	τ(H-N-H)
iii	191.5	190.8 (No. 140)	187.1	ν_s(C-N-C) + δ(H-N-H)	*xiv*	73.5	72.3 (No. 58)	71.2	ν_{as}(C-N-C)
iv	179.5	177.5 (No. 127)	178.2	ν_{as}(C-N-C) + ρ(H-N-H)	*xv*	59.9	64.1 (No. 49)	62.0	γ(N-H-N)
v	163.3	163.5 (No. 123)	160.1	ν_{as}(C-N-C) + ρ(H-N-H)	*xvi*	54.4	58.3 (No. 45)	56.0	γ(N-H-N)
vi	145.5	145.2 (No. 113)	143.1	ρ(H-N-H)	*xvii*	49.3	50.3 (No. 42)	49.5	δ(N-C-NH_2)
vii	128.4	128.5 (No. 107)	125.2	ρ(H-N-H)	*xviii*	47.6	48.3 (No. 38)	46.9	δ(N-C-NH_2)
viii	120.4	119.6 (No. 99)	113.3	R3–ring	*xix*	31.2	31.2 (No. 35)	29.9	γ(N-C-NH_2)
ix	110.1	107.6 (No. 95)	108.5	ω(H-N-H)	*xx*	30.1	30.1 (No. 34)	27.3	γ(N-C-NH_2)
x	98.7	94.2 (No. 84)	94.3	τ(H-N-H)	*xxi*	26.8	27.7 (No. 32)	24.9	γ(N-C-NH_2)
xi	90.6	89.1 (No. 76)	86.5	γ–ring	*xxii*	24.9	25.1 (No. 27)	23.6	τ(N-C-N)

Table S2. Main spectral features (ID) in the INS spectrum of melamine, assigned to the fundamental internal vibrational transitions. The experimentally observed (INS) energy transfers (in meV) are compared to the calculated ones in terms of both harmonic lattice dynamics (HLD) at 0 K and *ab initio* molecular dynamics simulations (MD) at 300 K. The labelling of normal mode in the HLD columns stay in line with Table S1 and Figs. S4–S15. Legend: ν_s, ν_{as} – symmetric and antisymmetric stretching; δ – bending; ρ – rocking; ω – wagging; τ – twisting; R3 – symmetric ring stretching; R6 – ring breathing; γ – out-of-plane deformation.

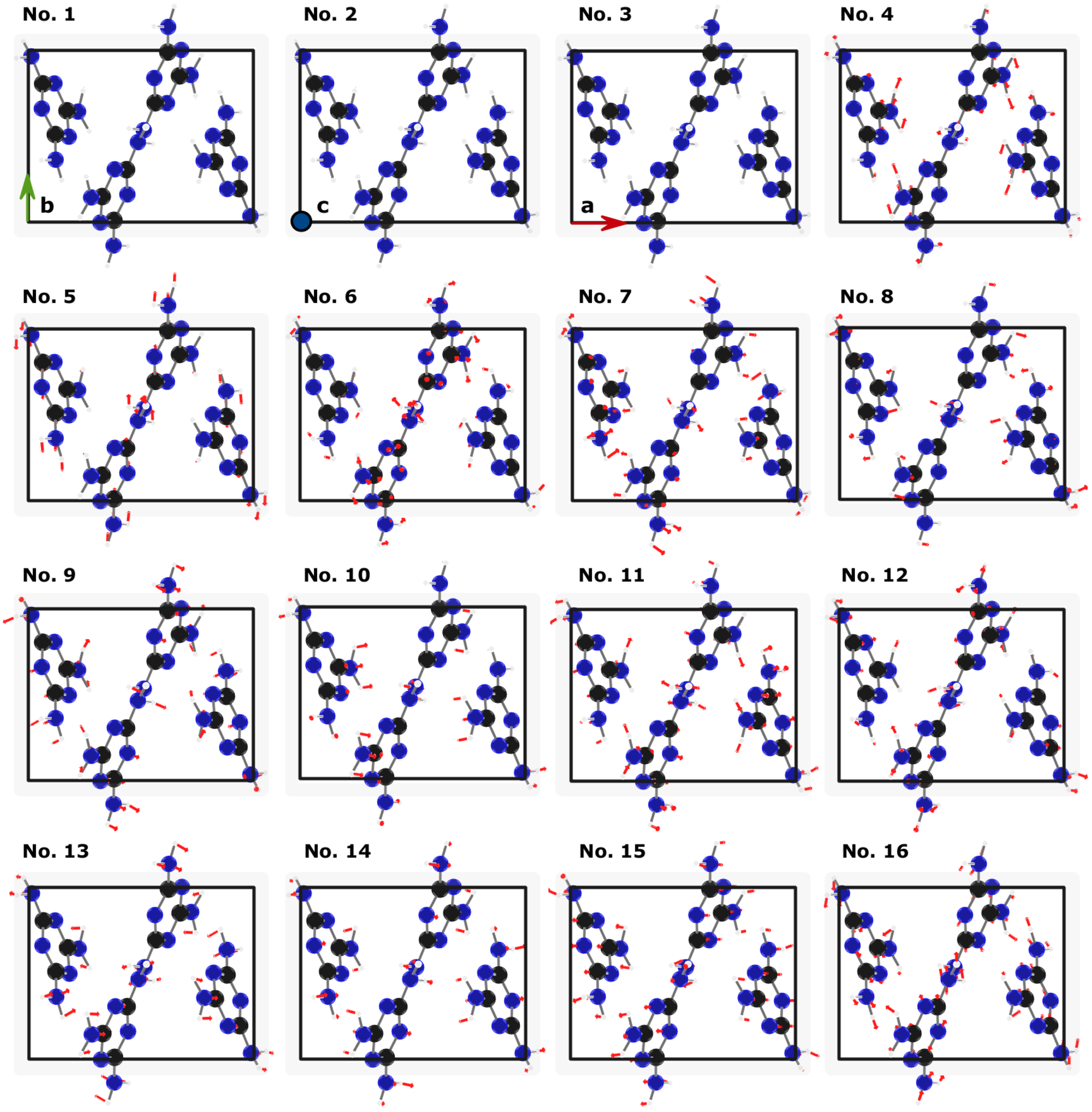

Figure S4. Schematic representation of the zone-centre modes eigenvectors (No. 1 - 16), according to harmonic lattice dynamics calculations (linear-response with PBE-TS). Atoms are shown as solid spheres: hydrogen (grey), carbon (black), and nitrogen (blue). Atomic displacements are shown as solid red vectors.

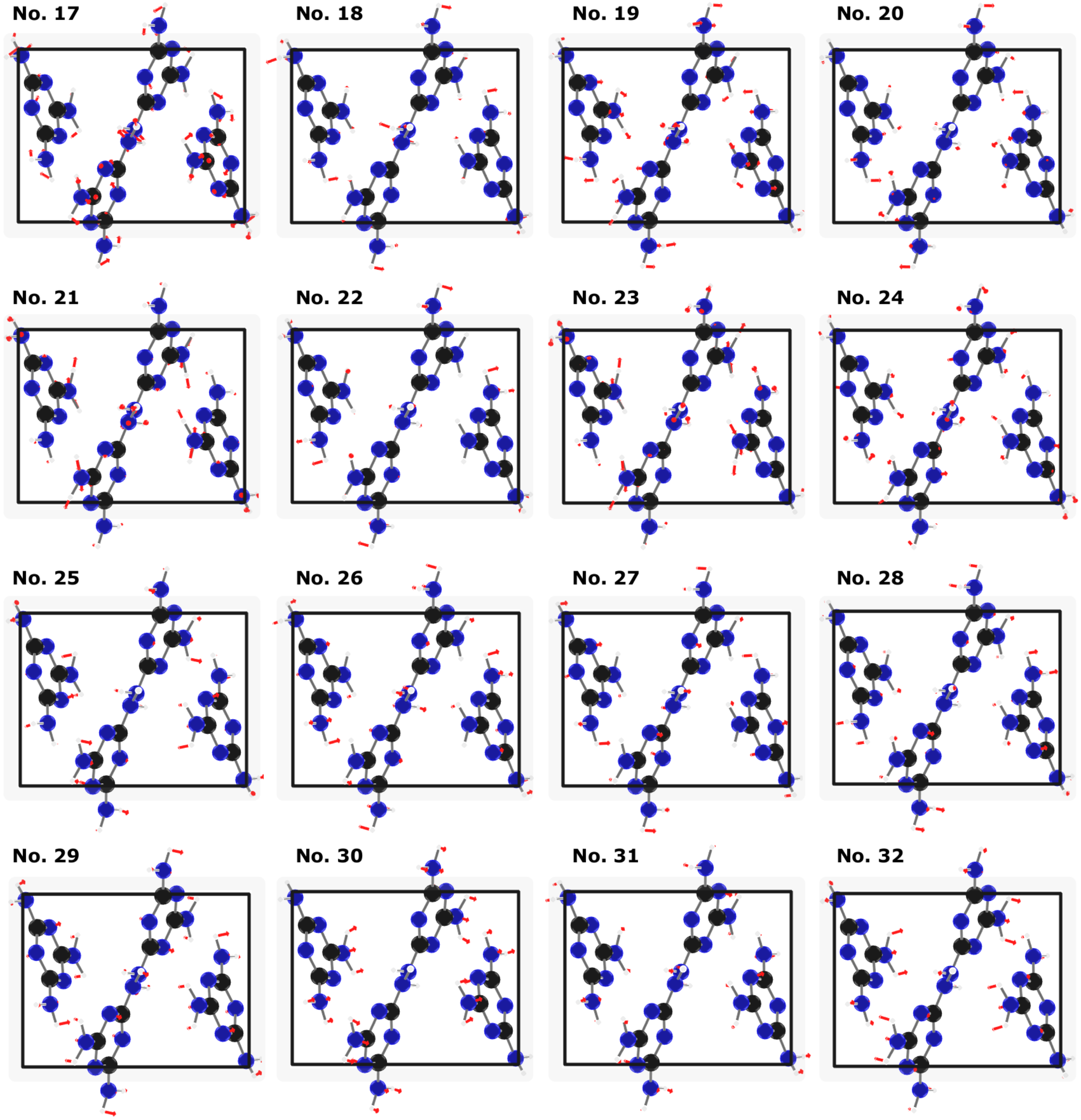

Figure S5. Schematic representation of the zone-centre modes eigenvectors (No. 17 - 32), according to harmonic lattice dynamics calculations (linear-response with PBE-TS). Atoms are shown as solid spheres: hydrogen (grey), carbon (black), and nitrogen (blue). Atomic displacements are shown as solid red vectors.

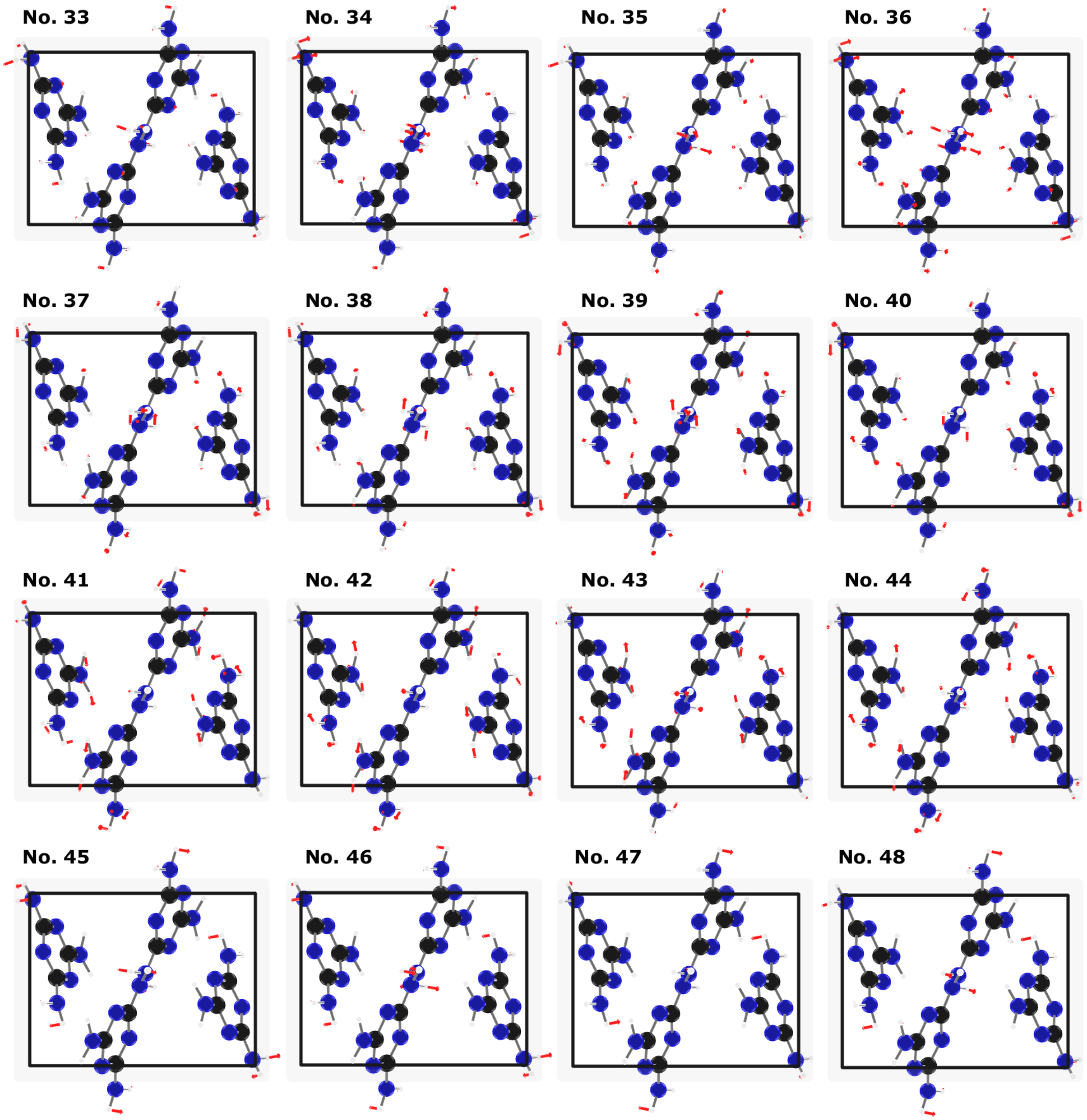

Figure S6. Schematic representation of the zone-centre modes eigenvectors (No. 33 - 48), according to harmonic lattice dynamics calculations (linear-response with PBE-TS). Atoms are shown as solid spheres: hydrogen (grey), carbon (black), and nitrogen (blue). Atomic displacements are shown as solid red vectors.

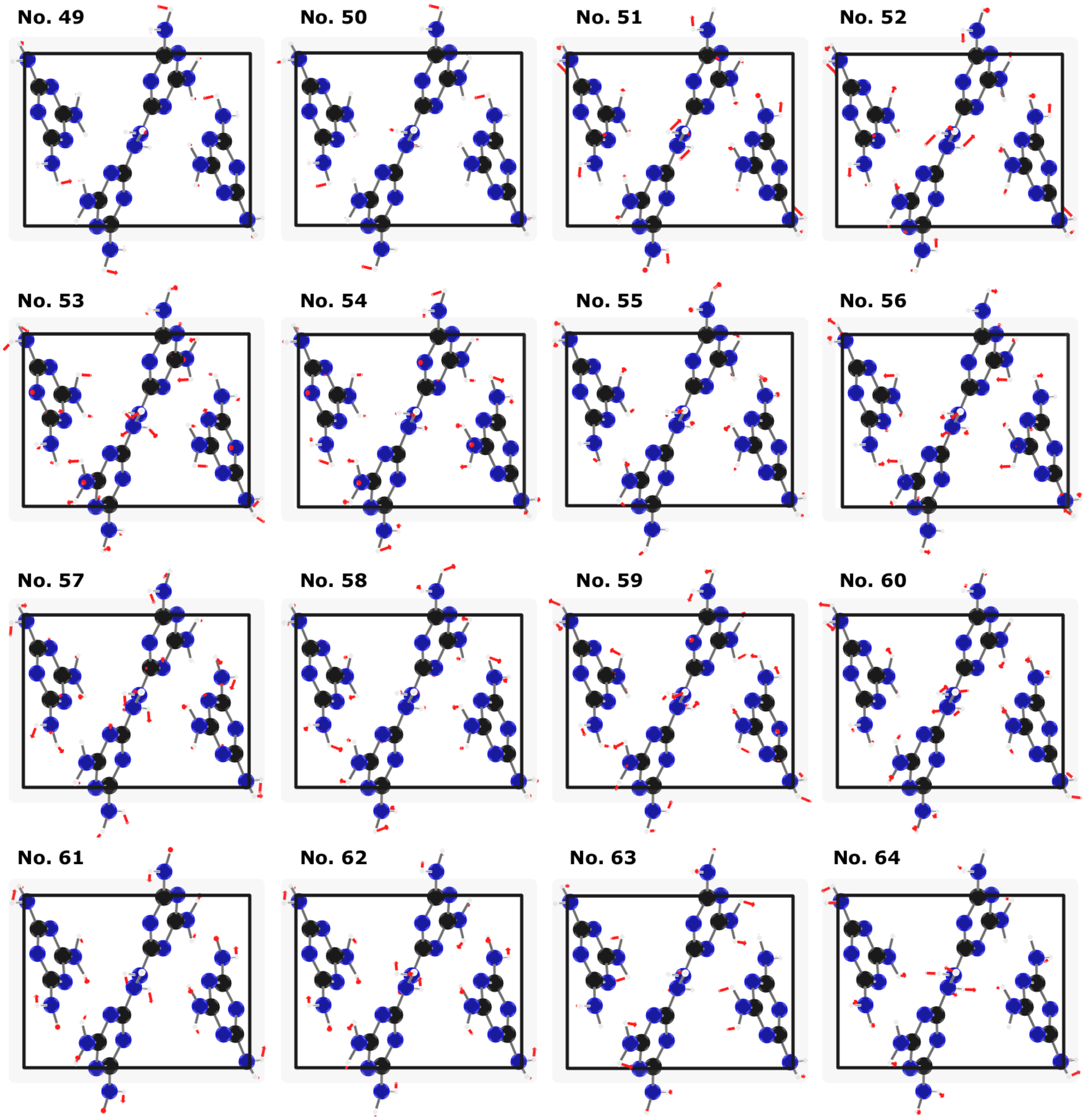

Figure S7. Schematic representation of the zone-centre modes eigenvectors (No. 49 - 64), according to harmonic lattice dynamics calculations (linear-response with PBE-TS). Atoms are shown as solid spheres: hydrogen (grey), carbon (black), and nitrogen (blue). Atomic displacements are shown as solid red vectors.

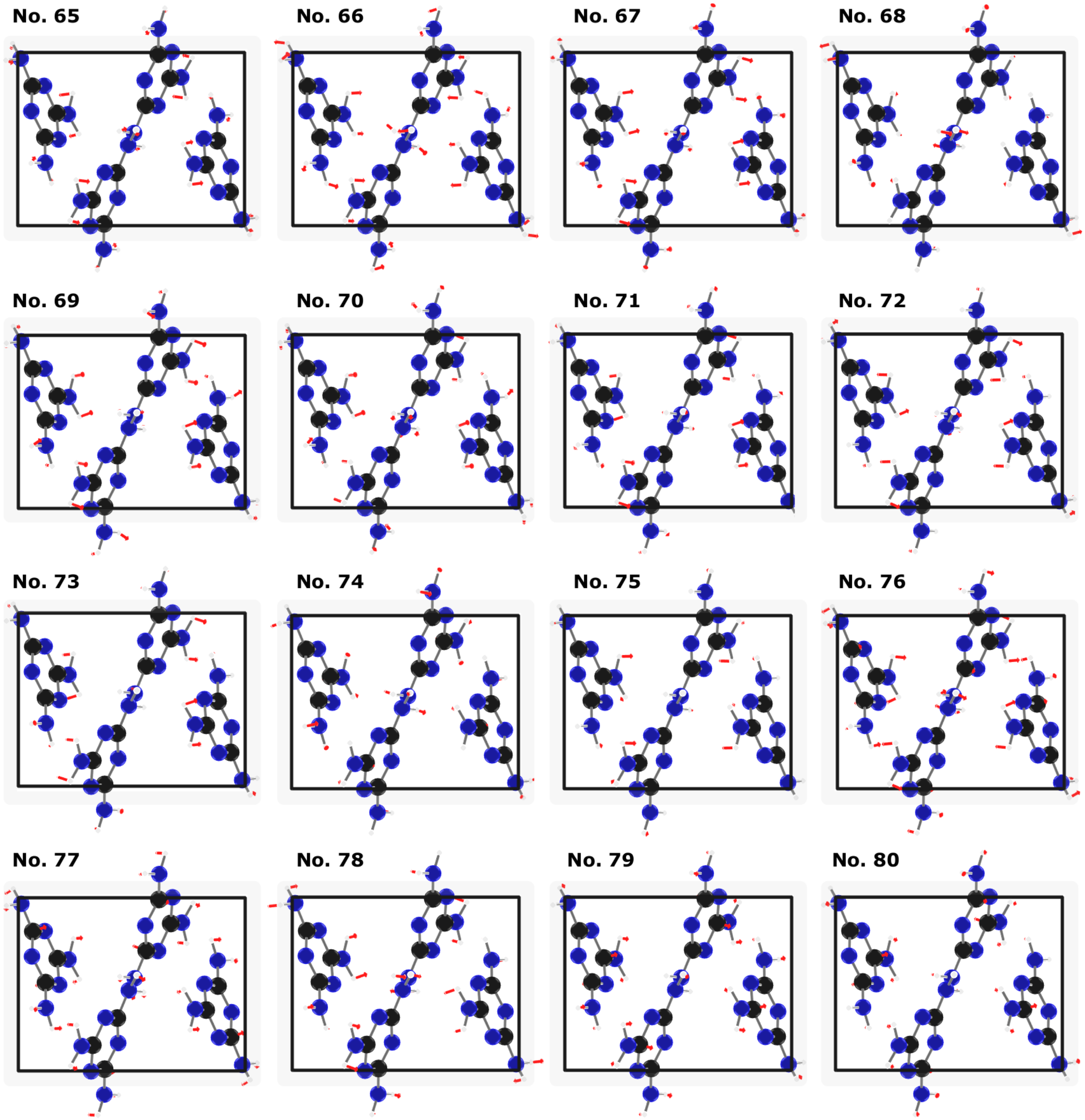

Figure S8. Schematic representation of the zone-centre modes eigenvectors (No. 65 - 80), according to harmonic lattice dynamics calculations (linear-response with PBE-TS). Atoms are shown as solid spheres: hydrogen (grey), carbon (black), and nitrogen (blue). Atomic displacements are shown as solid red vectors.

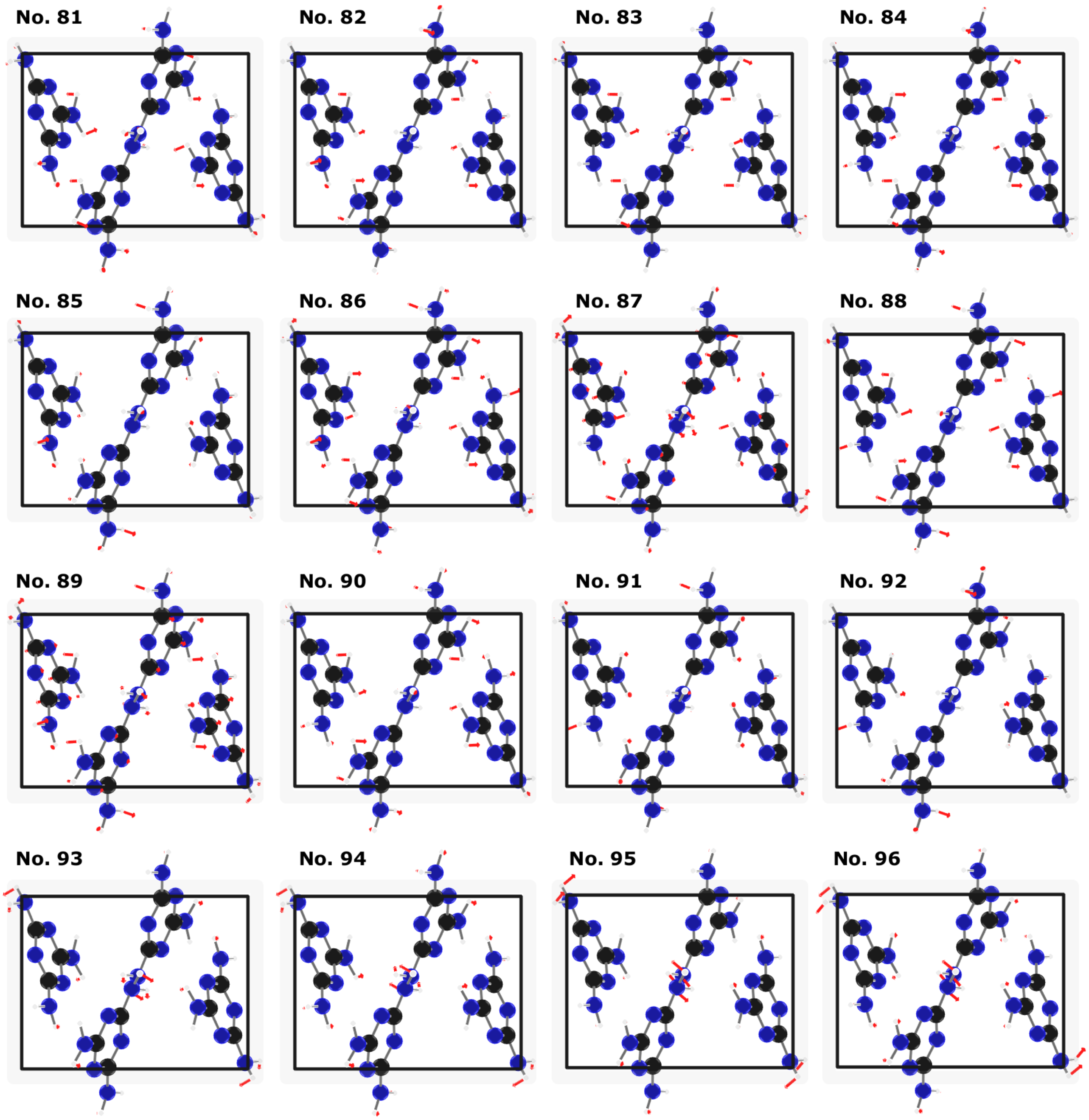

Figure S9. Schematic representation of the zone-centre modes eigenvectors (No. 81 - 96), according to harmonic lattice dynamics calculations (linear-response with PBE-TS). Atoms are shown as solid spheres: hydrogen (grey), carbon (black), and nitrogen (blue). Atomic displacements are shown as solid red vectors.

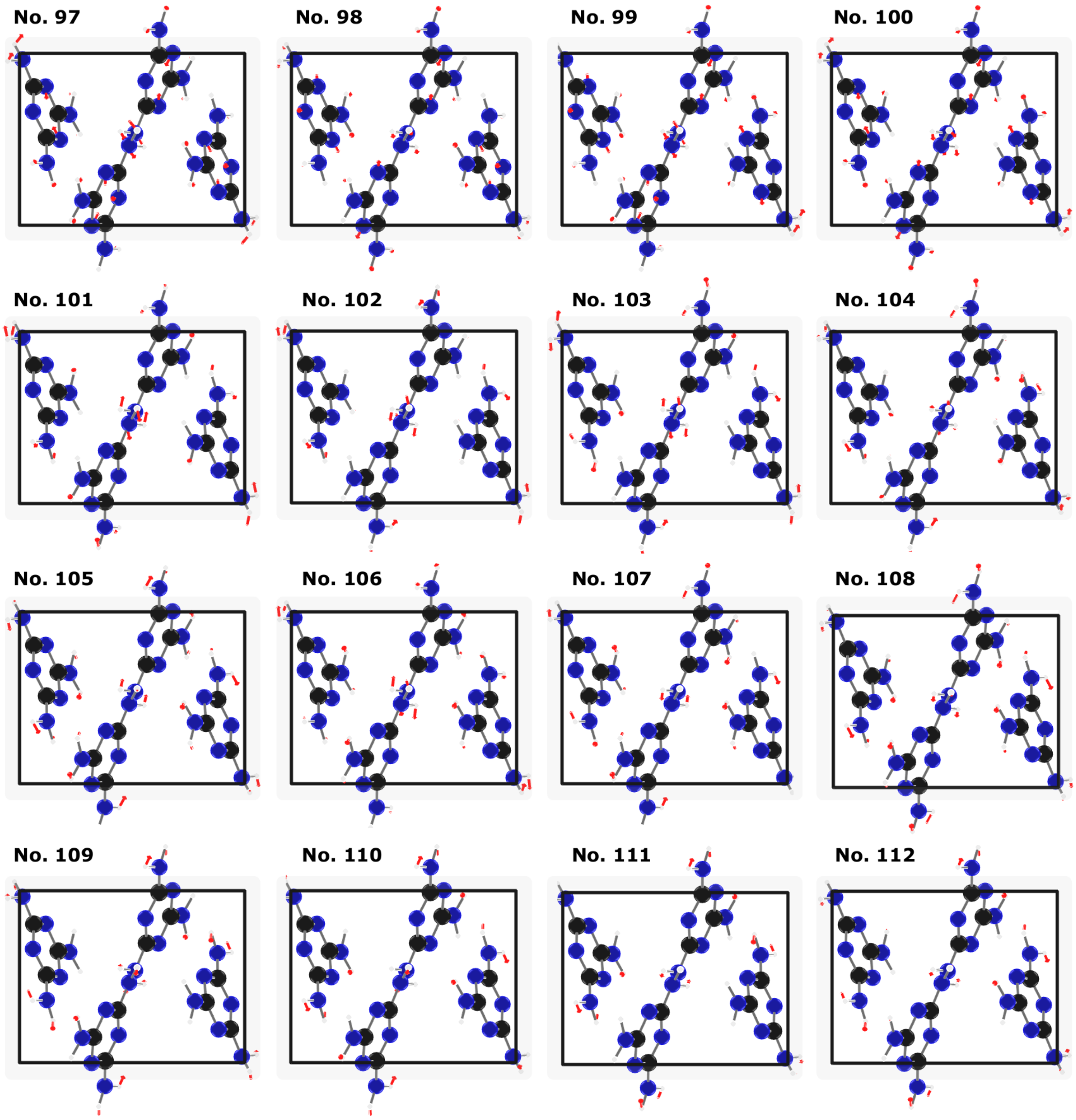

Figure S10. Schematic representation of the zone-centre modes eigenvectors (No. 97 - 112), according to harmonic lattice dynamics calculations (linear-response with PBE-TS). Atoms are shown as solid spheres: hydrogen (grey), carbon (black), and nitrogen (blue). Atomic displacements are shown as solid red vectors.

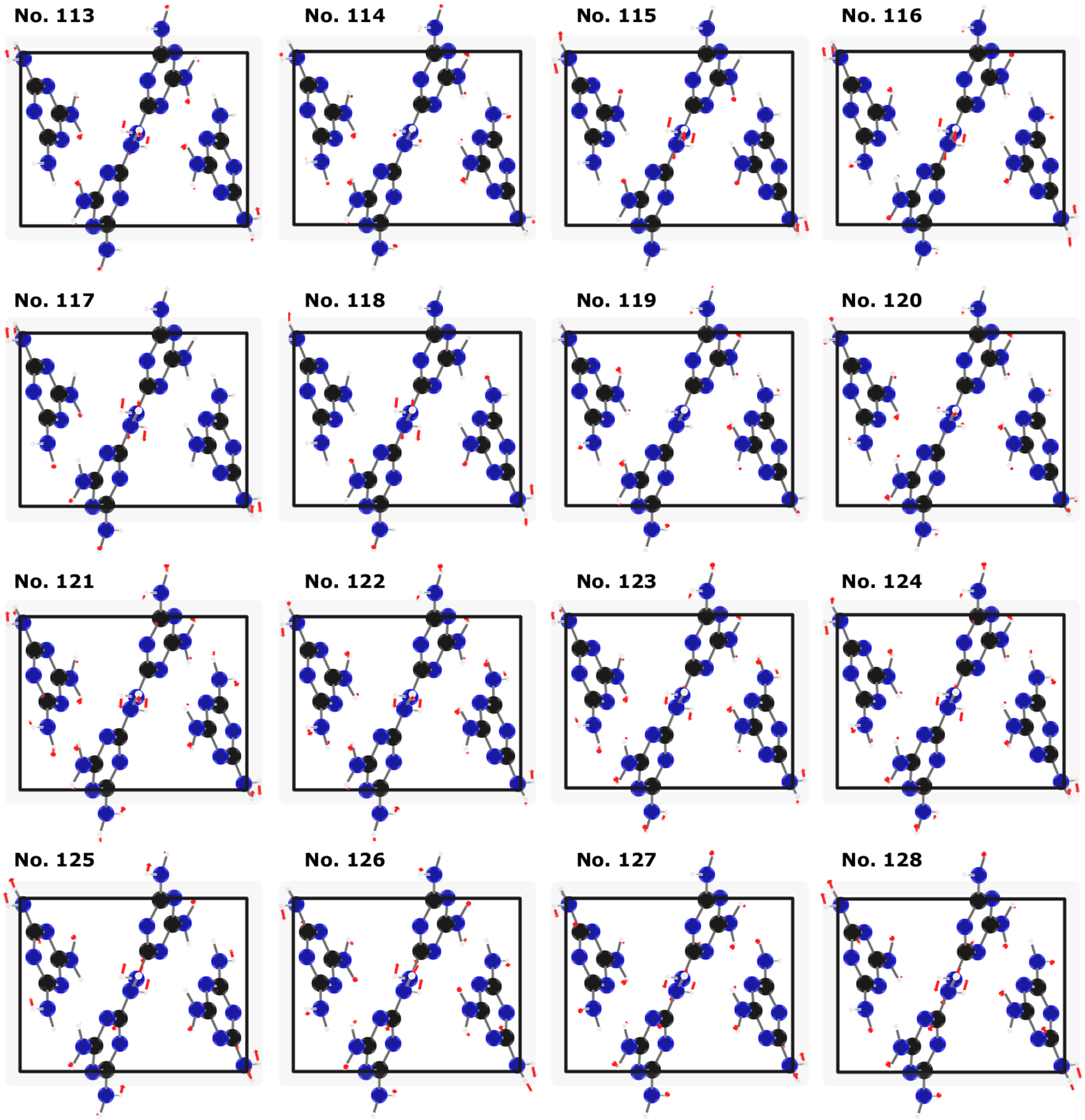

Figure S11. Schematic representation of the zone-centre modes eigenvectors (No. 113 - 128), according to harmonic lattice dynamics calculations (linear-response with PBE-TS). Atoms are shown as solid spheres: hydrogen (grey), carbon (black), and nitrogen (blue). Atomic displacements are shown as solid red vectors.

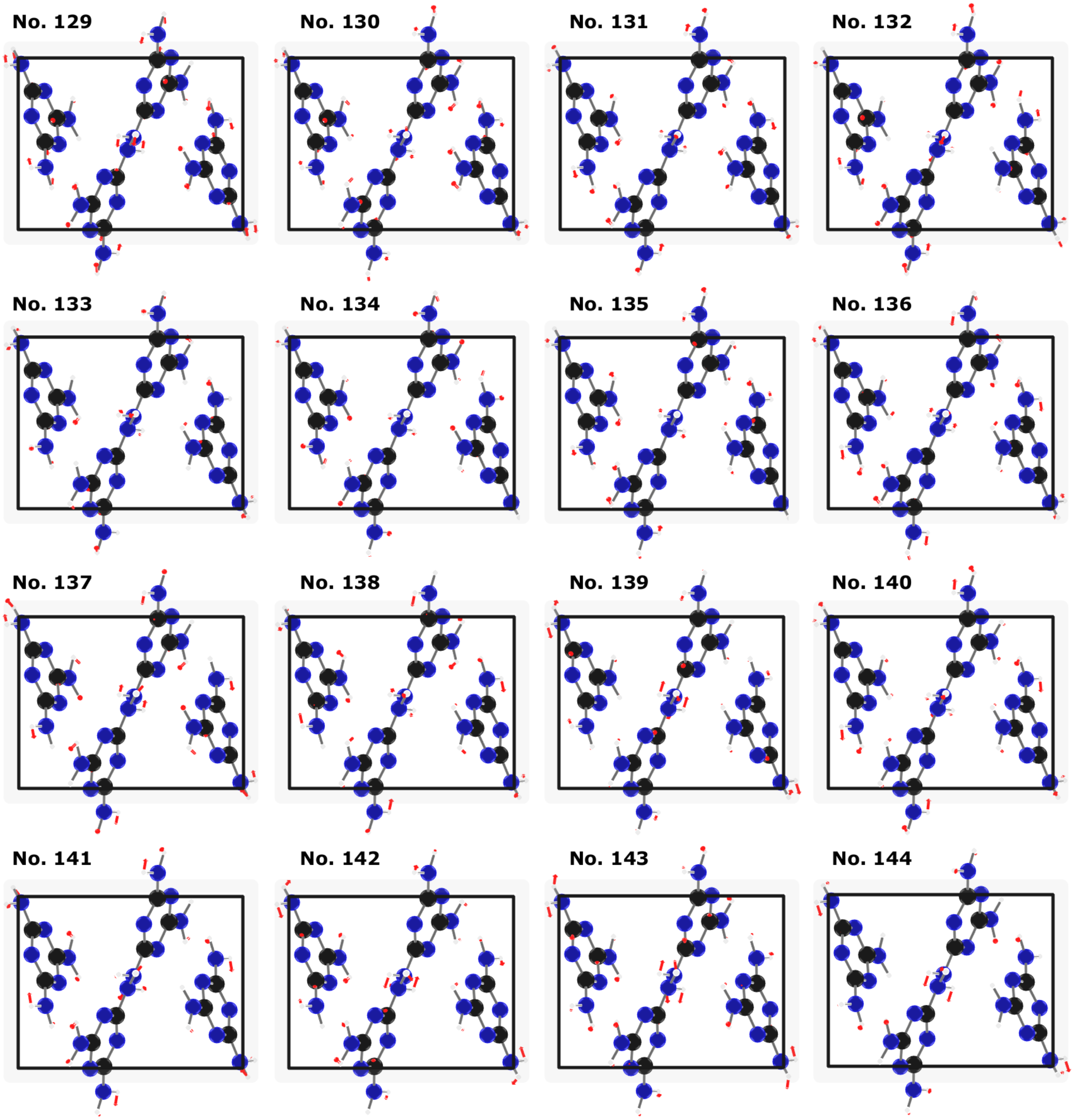

Figure S12. Schematic representation of the zone-centre modes eigenvectors (No. 129 - 144), according to harmonic lattice dynamics calculations (linear-response with PBE-TS). Atoms are shown as solid spheres: hydrogen (grey), carbon (black), and nitrogen (blue). Atomic displacements are shown as solid red vectors.

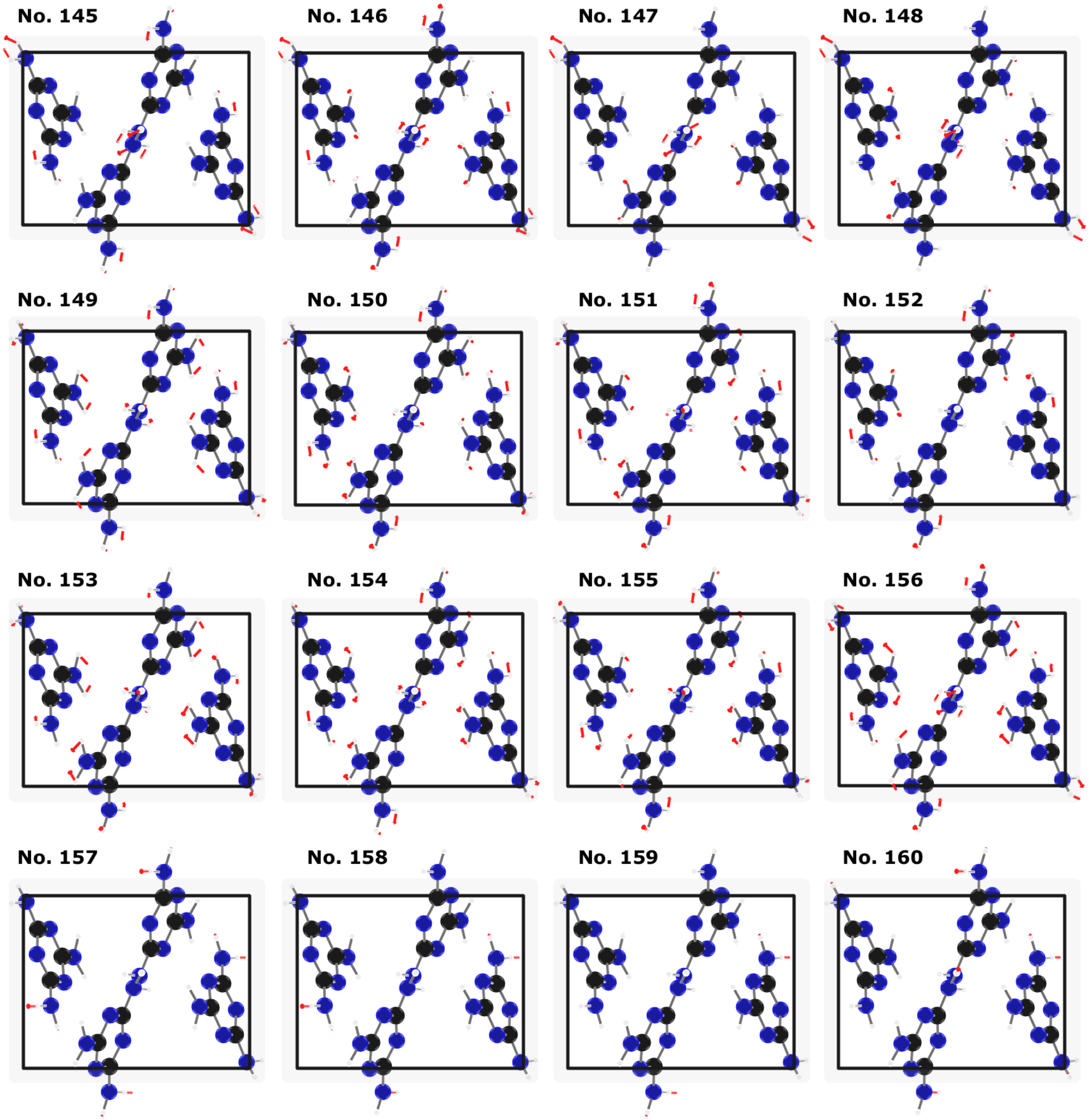

Figure S13. Schematic representation of the zone-centre modes eigenvectors (No. 145 - 160), according to harmonic lattice dynamics calculations (linear-response with PBE-TS). Atoms are shown as solid spheres: hydrogen (grey), carbon (black), and nitrogen (blue). Atomic displacements are shown as solid red vectors.

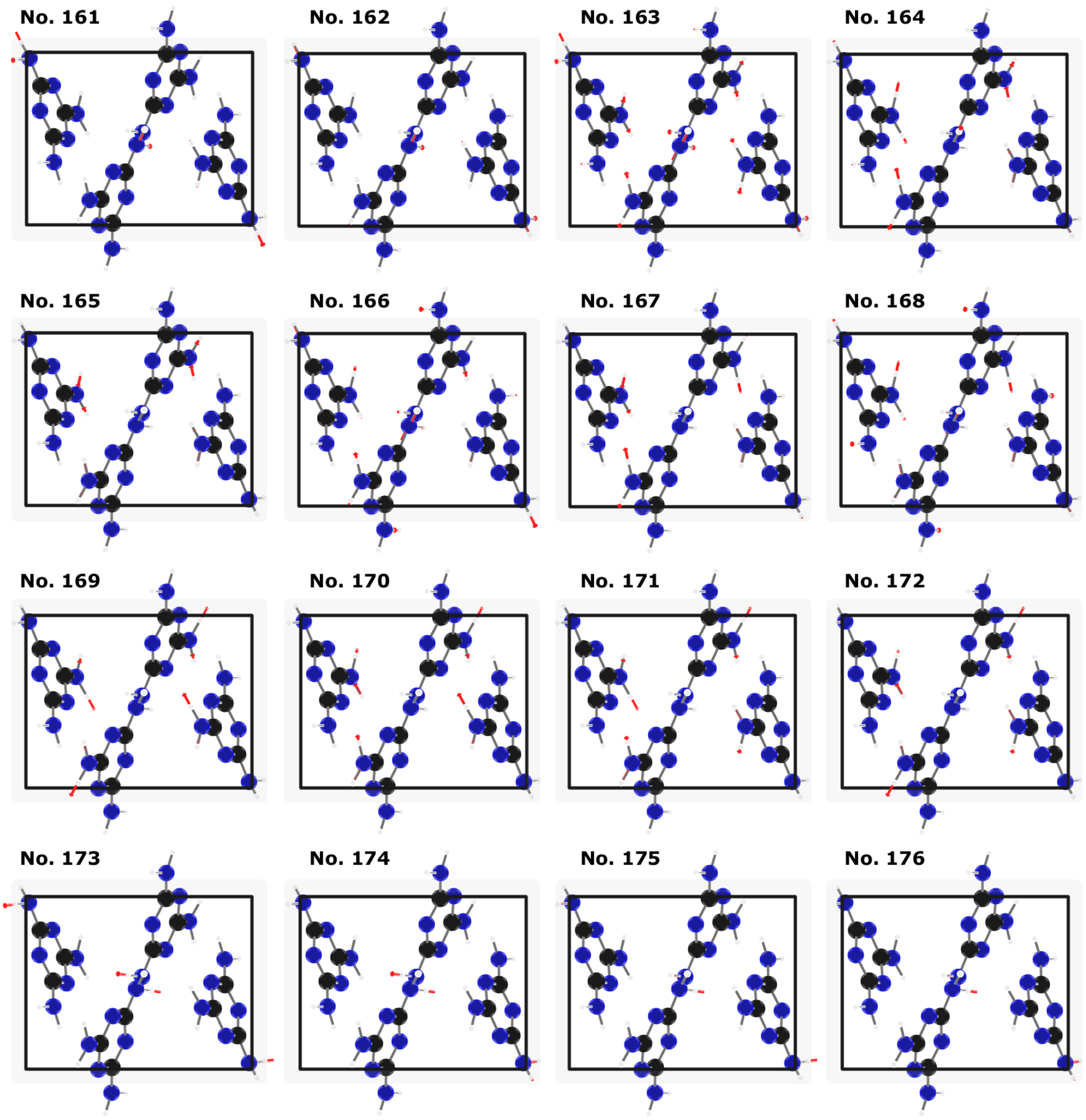

Figure S14. Schematic representation of the zone-centre modes eigenvectors (No. 161 - 176), according to harmonic lattice dynamics calculations (linear-response with PBE-TS). Atoms are shown as solid spheres: hydrogen (grey), carbon (black), and nitrogen (blue). Atomic displacements are shown as solid red vectors.

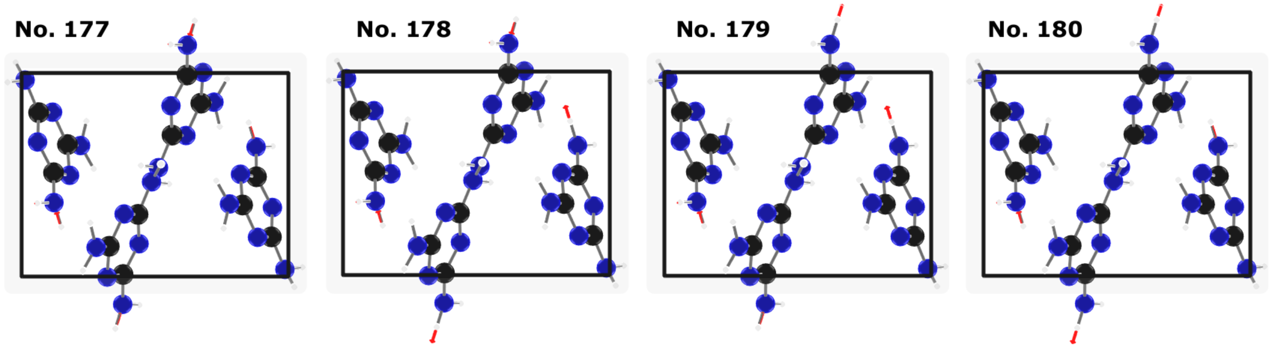

Figure S15. Schematic representation of the zone-centre modes eigenvectors (No. 177 - 180), according to harmonic lattice dynamics calculations (linear-response with PBE-TS). Atoms are shown as solid spheres: hydrogen (grey), carbon (black), and nitrogen (blue). Atomic displacements are shown as solid red vectors.

S3. Phonon properties beyond the Γ–point

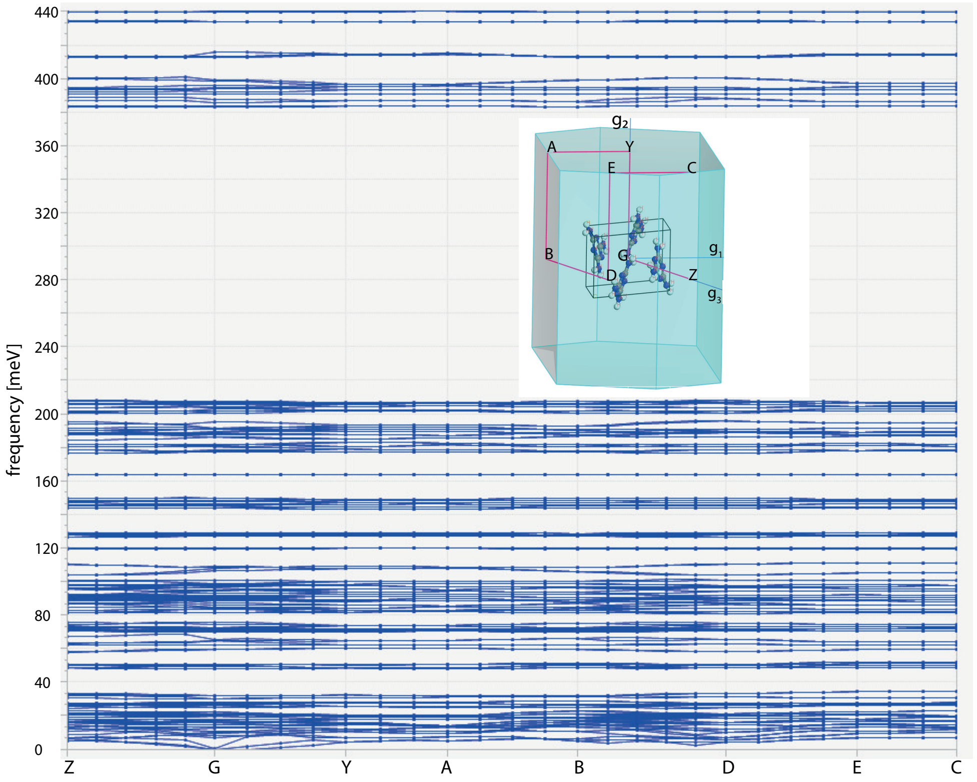

Figure S16. Mode dispersion in melamine as calculated from the output of the HLD simulation in CASTEP software. The inset shows the high symmetry points in the first Brillouin zone of the melamine crystal structure. For details, see the text of the main article.

component (x, y, z)	$\omega(q_1)$	$\omega(q_2)$	$\omega(q_3)$	$\omega(q_4)$	$\omega(q_5)$	$\omega(q_6)$	$\omega(q_7)$	$\omega(q_8)$	$\omega(q_9)$	$\omega(q_{10})$	$\omega(q_{11})$	$\omega(q_{12})$	$\omega(q_{13})$	$\omega(q_{14})$	$\omega(q_{15})$
x	0.0000	0.0000	0.0000	0.0000	0.0000	0.0000	0.0000	0.0000	0.0000	0.0000	−0.16667	−0.33333	−0.50000	−0.50000	−0.50000
y	0.0000	0.0000	0.0000	0.0000	0.0000	0.0000	0.12500	0.25000	0.37500	0.50000	0.50000	0.50000	0.50000	0.37500	0.25000
z	0.50000	0.40000	0.30000	0.20000	0.10000	0.0000	0.0000	0.0000	0.0000	0.0000	0.0000	0.0000	0.0000	0.0000	0.0000

component (x, y, z)	$\omega(q_{16})$	$\omega(q_{17})$	$\omega(q_{18})$	$\omega(q_{19})$	$\omega(q_{20})$	$\omega(q_{21})$	$\omega(q_{22})$	$\omega(q_{23})$	$\omega(q_{24})$	$\omega(q_{25})$	$\omega(q_{26})$	$\omega(q_{27})$	$\omega(q_{28})$	$\omega(q_{29})$
x	−0.50000	−0.50000	−0.50000	−0.50000	−0.50000	−0.50000	−0.50000	−0.50000	−0.50000	−0.50000	−0.50000	−0.33333	−0.16667	0.0000
y	0.12500	0.0000	0.0000	0.0000	0.0000	0.0000	0.0000	0.12500	0.25000	0.37500	0.50000	0.50000	0.50000	0.50000
z	0.0000	0.0000	0.10000	0.20000	0.30000	0.40000	0.50000	0.50000	0.50000	0.50000	0.50000	0.50000	0.50000	0.50000

Table S3. List of q-points for the mode dispersion HLD simulation in CASTEP software for melamine.
For details, see the main article text.

Mode no.	$\omega(q_1)$	$\omega(q_2)$	$\omega(q_3)$	$\omega(q_4)$	$\omega(q_5)$	$\omega(q_6)$	$\omega(q_7)$	$\omega(q_8)$	$\omega(q_9)$	$\omega(q_{10})$	$\omega(q_{11})$	$\omega(q_{12})$	$\omega(q_{13})$	$\omega(q_{14})$	$\omega(q_{15})$	$\omega(q_{16})$	$\omega(q_{17})$	$\omega(q_{18})$	$\omega(q_{19})$	$\omega(q_{20})$	$\omega(q_{21})$	$\omega(q_{22})$	$\omega(q_{23})$	$\omega(q_{24})$	$\omega(q_{25})$	$\omega(q_{26})$	$\omega(q_{27})$	$\omega(q_{28})$	$\omega(q_{29})$
1	4.6283	4.7610	4.2365	3.0079	1.5548	0.0000	0.0000	0.0000	0.94166	2.4462	4.4672	4.9842	5.2780	5.0040	5.6177	6.2054	5.9698	4.9656	3.7270	4.6643	4.4411	2.5962	4.3952	4.4510	5.2693	6.3592	6.8328	6.7795	6.6790
2	5.0573	5.3078	4.5254	3.7629	2.1313	0.0000	0.0000	1.8560	3.7071	5.2049	4.4672	4.9842	5.2780	5.0040	5.6177	6.2054	5.9698	4.9656	5.7144	5.3586	4.4498	4.0555	4.3952	4.4510	5.2693	6.3592	6.8328	6.7795	6.6790
3	6.3083	5.3487	6.5724	5.8682	5.4156	0.091029	0.20718	7.3585	8.6615	6.7026	6.6046	6.4745	6.8092	7.3560	7.6411	7.2965	6.1843	6.1112	5.7219	5.5843	6.4013	6.0046	5.2557	5.9401	7.1254	8.2896	9.2405	9.4699	9.7340
4	6.4621	6.7919	6.9072	7.7751	7.9003	6.9406	6.9406	7.9895	8.6839	8.1966	6.6046	6.4745	6.8092	7.3560	7.6411	7.2965	6.1843	6.1112	7.1849	7.4105	7.0237	7.0014	5.2557	5.9401	7.1254	8.2896	9.2405	9.4699	9.7340
5	6.6418	7.2667	7.0721	8.2363	8.6367	7.9573	7.9536	8.3603	9.2616	9.8258	10.447	10.113	9.5778	9.4662	10.234	11.116	10.601	10.386	9.3596	8.2995	7.1465	7.0262	6.8340	8.0342	10.167	11.221	10.406	10.659	11.385
6	8.6938	8.8760	9.8753	9.9981	8.8946	9.1091	9.1364	9.9373	9.4488	10.308	10.447	10.113	9.5778	9.4662	10.234	11.116	10.601	10.386	11.240	11.118	9.3968	8.3441	6.8340	8.0342	10.167	11.221	10.406	10.659	11.385
7	10.935	11.088	10.922	11.035	9.5443	10.245	10.245	10.257	10.389	11.015	11.663	11.407	10.845	10.404	10.730	11.920	13.142	13.266	12.671	11.703	10.722	8.7768	9.7910	10.420	11.641	12.436	13.353	13.018	12.671
8	11.746	11.764	11.948	12.076	11.167	10.425	10.784	10.374	11.761	11.433	11.663	11.407	10.845	10.404	10.730	11.920	13.142	13.266	12.845	12.039	11.673	11.141	9.7910	10.420	11.641	12.436	13.353	13.018	12.671
9	12.324	12.026	12.510	12.584	11.977	12.460	12.460	12.473	12.597	12.288	11.699	11.942	12.473	13.080	12.820	12.882	13.663	14.209	13.837	13.502	12.845	11.523	11.974	12.121	12.944	14.184	14.804	14.494	13.812
10	12.994	13.564	13.328	12.746	13.142	13.242	13.242	12.808	12.733	12.309	11.699	11.942	12.473	13.080	12.820	12.882	13.663	14.209	14.568	14.023	12.907	12.473	11.974	12.121	12.944	14.184	14.804	14.494	13.812
11	13.440	13.613	13.403	13.192	13.279	13.316	13.316	13.403	13.737	13.911	14.531	14.295	13.812	13.341	13.180	13.601	14.705	15.151	14.705	14.667	14.047	14.469	14.717	14.407	14.035	14.692	15.324	15.238	14.779
12	14.432	14.072	14.816	14.407	14.494	14.444	14.444	14.271	14.581	14.680	14.531	14.295	13.812	13.341	13.180	13.601	14.705	15.151	15.647	15.101	15.089	14.866	14.717	14.407	14.035	14.692	15.324	15.238	14.779
13	15.833	15.399	15.275	15.386	15.535	15.461	15.498	15.076	14.680	14.977	15.386	15.089	14.407	14.023	13.936	14.977	15.882	16.403	15.870	15.486	15.548	15.535	15.461	15.907	16.478	16.354	16.118	16.205	16.292
14	15.907	16.031	15.647	15.572	15.833	15.882	15.845	15.696	15.461	15.473	15.386	15.089	14.407	14.023	13.936	14.977	15.882	16.403	16.589	16.453	15.771	15.597	15.461	15.907	16.478	16.354	16.118	16.205	16.292
15	15.920	16.242	16.242	16.440	16.663	16.713	16.713	16.081	15.548	15.721	16.081	15.672	14.692	14.035	15.312	16.329	17.035	17.221	16.775	16.725	16.453	15.634	15.672	16.341	17.283	17.693	17.643	17.333	17.110
16	16.478	16.453	16.552	16.527	16.763	16.874	16.874	16.465	16.192	16.490	16.081	15.672	14.692	14.035	15.312	16.329	17.035	17.221	17.544	17.234	17.445	16.601	15.672	16.341	17.283	17.693	17.643	17.333	17.110
17	17.717	17.333	16.763	16.564	16.800	17.135	17.135	17.445	17.705	17.283	17.407	17.531	17.668	17.705	17.866	18.089	18.089	18.077	17.556	17.804	18.003	18.102	18.350	18.399	18.312	18.102	17.866	18.275	18.759
18	17.817	17.631	17.581	17.866	18.015	17.990	17.866	17.941	17.804	17.693	17.407	17.531	17.668	17.705	17.866	18.089	18.089	18.077	18.151	18.139	18.077	18.374	18.350	18.399	18.312	18.102	17.866	18.275	18.759
18	18.734	19.044	19.019	18.759	19.007	19.490	19.540	18.957	18.709	18.498	18.846	18.833	18.784	18.771	18.759	18.796	19.007	19.106	19.019	19.193	19.131	19.218	19.515	19.515	19.862	20.160	20.160	19.986	19.652
19	18.957	19.230	19.701	19.726	19.577	19.540	19.763	20.185	19.515	19.391	18.846	18.833	18.784	18.771	18.759	18.796	19.007	19.106	19.713	19.825	19.751	19.937	19.515	19.515	19.862	20.160	20.160	19.986	19.652
20	19.999	20.036	20.259	20.606	20.581	20.259	20.247	20.209	19.949	19.639	19.887	19.800	19.763	19.788	19.713	20.185	20.743	20.953	20.594	20.482	20.519	20.135	20.234	20.470	20.705	20.792	20.879	20.631	20.222
21	20.705	20.743	20.941	21.152	21.139	21.127	21.127	20.867	20.346	20.172	19.887	19.800	19.763	19.788	19.713	20.185	20.743	20.953	21.090	21.090	20.991	20.457	20.234	20.470	20.705	20.792	20.879	20.631	20.222
22	21.548	21.499	21.400	21.511	21.883	21.958	21.958	21.325	20.619	20.309	20.271	20.222	20.098	20.023	20.569	21.437	22.330	22.726	22.454	21.821	21.177	21.177	21.201	21.313	21.685	22.094	22.255	22.491	22.788
23	22.652	22.553	22.280	21.995	21.920	22.044	22.044	21.734	21.189	20.656	20.271	20.222	20.098	20.023	20.569	21.437	22.330	22.726	22.652	22.255	21.710	21.350	21.201	21.313	21.685	22.094	22.255	22.491	22.788
24	24.425	24.512	24.760	24.722	24.574	24.512	24.524	24.598	24.797	25.156	25.404	25.256	25.070	25.045	24.983	24.970	24.946	24.884	24.673	24.499	24.338	24.289	24.474	24.859	25.665	25.851	25.876	25.863	25.851
25	25.256	25.156	24.946	25.132	25.528	25.826	25.851	25.578	25.342	25.355	25.404	25.256	25.070	25.045	24.983	24.970	24.946	24.884	25.144	25.417	25.169	24.784	24.474	24.859	25.665	25.851	25.876	25.863	25.851
26	25.876	25.938	26.074	26.148	26.223	26.136	26.136	25.888	25.640	25.479	25.640	25.603	25.429	25.280	25.442	25.690	25.950	26.148	25.975	25.677	25.900	25.938	26.061	26.012	25.987	26.533	26.867	26.867	26.830
27	26.334	26.297	26.235	26.334	26.433	26.272	26.272	26.533	26.495	26.123	25.640	25.603	25.429	25.280	25.442	25.690	25.950	26.148	26.099	25.975	25.938	26.198	26.061	26.012	25.987	26.533	26.867	26.867	26.830
28	26.867	26.929	26.967	26.781	26.471	26.533	26.508	26.681	26.917	27.277	27.450	27.512	27.574	27.599	27.301	26.979	26.855	26.793	26.743	26.681	26.719	26.781	26.967	27.016	27.128	27.239	27.314	27.326	27.363
29	27.289	27.289	27.227	27.153	27.053	26.991	26.979	27.115	27.401	27.487	27.450	27.512	27.574	27.599	27.301	26.979	26.855	26.793	26.818	26.805	26.743	26.843	26.967	27.016	27.128	27.239	27.314	27.326	27.363
30	27.363	27.314	27.388	27.661	27.648	27.673	27.673	27.673	27.673	27.723	27.896	27.896	27.896	27.909	28.120	28.330	28.492	28.554	28.368	27.996	27.574	27.277	27.301	27.376	27.549	27.599	27.525	27.574	27.686
31	27.463	27.537	27.648	27.686	28.008	28.144	28.144	28.132	28.132	28.070	27.896	27.896	27.896	27.909	28.120	28.330	28.492	28.554	28.504	28.268	27.983	27.673	27.301	27.376	27.549	27.599	27.525	27.574	27.686
32	30.575	30.463	30.178	29.967	30.004	30.165	30.165	30.240	30.413	30.550	30.587	30.674	30.835	30.922	31.008	31.194	31.269	31.256	30.922	30.798	30.860	30.984	31.157	31.058	30.872	30.773	30.773	30.723	30.637
33	31.827	31.752	31.604	31.504	30.946	30.562	30.165	30.302	30.537	30.624	30.587	30.674	30.835	30.922	31.008	31.194	31.269	31.256	31.554	31.554	31.480	31.343	31.157	31.058	30.872	30.773	30.773	30.723	30.637
34	32.819	32.682	32.248	31.604	31.554	31.678	31.703	31.777	31.963	32.112	32.224	32.174	32.075	32.013	31.938	31.802	31.814	31.914	32.000	32.248	32.620	32.943	33.116	33.290	33.687	34.034	34.170	34.170	34.145
35	33.426	33.339	33.104	32.732	32.335	32.075	32.075	32.038	32.013	32.162	32.224	32.174	32.075	32.013	31.938	31.802	31.814	31.914	32.075	32.533	32.905	33.104	33.116	33.290	33.687	34.034	34.170	34.170	34.145
36	48.379	48.242	48.069	47.994	47.982	47.982	47.982	47.994	48.031	48.118	48.292	48.267	48.205	48.180	48.069	48.143	48.317	48.416	48.329	48.230	48.205	48.267	48.366	48.304	48.379	48.490	48.540	48.527	48.503
38	48.465	48.304	48.230	48.180	48.180	48.180	48.180	48.131	48.069	48.155	48.292	48.267	48.205	48.180	48.069	48.143	48.317	48.416	48.428	48.391	48.317	48.292	48.366	48.304	48.379	48.490	48.540	48.527	48.503
39	48.490	48.627	48.850	49.098	49.284	49.346	49.482	49.333	49.023	48.738	48.540	48.490	48.391	48.341	48.701	49.209	49.581	49.705	49.618	49.383	49.036	48.651	48.490	48.788	49.284	49.742	49.953	49.953	49.978
40	48.540	48.813	49.147	49.457	49.705	49.804	49.631	49.569	49.296	48.875	48.540	48.490	48.391	48.341	48.701	49.209	49.581	49.705	49.643	49.420	49.085	48.751	48.490	48.788	49.284	49.742	49.953	49.953	49.978
41	50.015	50.040	50.102	50.189	50.251	50.276	50.276	50.288	50.288	50.201	50.152	50.164	50.214	50.238	50.313	50.474	50.610	50.648	50.536	50.338	50.114	49.990	49.978	50.102	50.449	50.809	50.933	50.933	50.920
42	50.164	50.176	50.226	50.325	50.424	50.437	50.437	50.424	50.375	50.226	50.152	50.164	50.214	50.238	50.313	50.474	50.610	50.648	50.586	50.362	50.164	50.028	49.978	50.102	50.449	50.809	50.933	50.933	50.920
43	50.561	50.548	50.511	50.511	50.548	x50.610	50.685	50.672	50.660	50.660	50.672	50.672	50.660	50.660	50.734	50.846	50.908	50.945	50.871	50.796	50.710	50.635	50.610	50.796	51.255	51.726	51.912	51.887	51.825
44	50.710	50.734	50.772	50.784	50.784	50.796	50.784	50.747	50.685	50.672	50.672	50.672	50.660	50.660	50.734	50.846	50.908	50.945	50.958	50.871	50.747	50.660	50.610	50.796	51.255	51.726	51.912	51.887	51.825
45	57.814	57.963	58.310	58.669	58.917	59.004	59.463	59.525	59.723	59.984	60.120	60.157	60.232	60.269	60.120	59.760	59.426	59.289	59.017	58.657	58.372	58.223	58.223	58.347	58.707	59.190	59.450	59.463	59.488
46	58.397	58.521	58.843	59.252	59.562	59.686	59.674	59.773	59.959	60.095	60.120	60.157	60.232	60.269	60.120	59.760	59.426	59.289	59.277	59.017	58.682	58.384	58.223	58.347	58.707	59.190	59.450	59.463	59.488
47	61.905	61.955	62.054	62.116	62.104	62.091	62.091	62.042	61.918	61.881	62.178	62.228	62.339	62.401	62.401	62.414	62.376	62.339	62.364	62.389	62.414	62.525	62.612	62.525	62.327	62.067	61.918	61.918	61.943
48	63.034	63.120	63.232	63.331	63.443	63.480	64.422	64.125	63.492	62.786	62.178	62.228	62.339	62.401	62.401	62.414	62.376	62.339	62.389	62.414	62.500	62.587	62.612	62.525	62.327	62.067	61.918	61.918	61.943
49	63.344	63.381	63.592	63.939	64.273	64.422	64.906	64.732	64.410	64.323	64.658	64.707	64.831	64.881	64.993	65.265	65.637	65.860	64.881	64.447	64.707	65.141	65.538	65.340	64.819	64.236	63.988	64.075	64.224
50	67.336	67.447	67.782	68.241	68.687	68.898	65.563	65.662	65.588	65.191	64.658	64.707	64.831	64.881	64.993	65.265	65.637	65.860	66.406	66.369	66.146	65.873	65.538	65.340	64.819	64.236	63.988	64.075	64.224
51	70.311	70.237	70.125	70.002	69.840	69.754	69.754	69.828	69.940	69.915	70.026	70.076	70.187	70.262	70.138	70.039	70.014	69.977	69.964	70.076	70.187	70.212	70.249	70.249	70.299	70.411	70.522	70.510	70.522
52	70.485	70.485	70.497	70.497	70.485	70.460	70.460	70.237	70.212	70.274	70.026	70.076	70.187	70.262	70.138	70.039	70.014	69.977	70.163	70.386	70.460	70.336	70.249	70.249	70.299	70.411	70.522	70.510	70.522
53	70.609	70.621	70.621	70.572	70.510	70.522	70.535	70.448	70.535	70.535	70.745	70.683	70.559	70.485	70.609	70.597	70.535	70.547	70.547	70.708	70.820	70.931	71.093	70.956	70.832	70.721	70.609	70.634	70.634
54	70.621	70.708	70.745	70.609	70.547	70.535	70.547	70.783	70.634	70.733	70.745	70.683	70.559	70.485	70.609	70.597	70.535	70.547	70.708	70.969	70.956	71.080	71.093	70.956	70.832	70.721	70.609	70.634	70.634
55	71.105	71.130	71.192	71.179	71.142	70.981	70.981	70.857	70.845	70.981	71.155	71.204	71.303	71.353	71.316	71.328	71.328	71.341	71.130	70.981	71.031	71.155	71.217	71.353	71.465	71.551	71.589	71.551	71.514
56	71.626	71.427	71.254	71.254	71.155	71.130	71.130	71.266	71.365	71.303	71.155	71.204	71.303	71.353	71.316	71.328	71.328	71.341	71.266	71.031	71.217	71.279	71.217	71.353	71.465	71.551	71.589	71.551	71.514
57	71.750	71.923	72.122	71.700	71.440	71.427	71.663	71.725	71.824	71.861	71.948	71.936	71.911	71.886	71.836	71.712	71.675	71.638	71.564	71.650	71.688	71.799	71.985	71.973	71.948	71.923	71.911	71.998	72.134
58	72.556	72.444	72.122	72.320	72.345	72.233	72.233	72.184	72.047	72.010	71.948	71.936	71.911	71.886	71.836	71.712	71.675	71.638	71.960	72.184	72.246	72.159	71.985	71.973	71.948	71.923	71.911	71.998	72.134
59	72.878	72.866	72.866	72.605	72.494	72.630	72.233	72.221	72.469	73.039	73.597	73.535	73.436	73.386	73.138	72.816	72.580	72.481	72.407	72.518	72.692	72.952	73.287	73.188	73.014	72.940	72.952	72.940	72.915
60	73.560	73.324	72.952	72.878	72.828	72.692	72.630	72.717	73.014	73.386	73.597	73.535	73.436	73.386	73.138	72.816	72.580	72.481	72.766	73.213	73.721	73.634	73.287	73.188	73.014	72.940	72.952	72.940	72.915
61	74.353	74.601	74.986	75.147	75.023	74.973	74.353	74.304	74.118	73.857	73.783	73.808	73.857	73.882	74.167	74.564	74.874	74.986	74.701	74.391	74.081	74.391	74.911	74.837	74.688	74.527	74.440	74.440	74.440
62	75.432	75.382	75.271	75.308	75.531	75.606	75.606	75.457	75.023	74.415	73.783	73.808	73.857	73.882	74.167	74.564	74.874	74.986	75.258	75.469	75.506	75.308	74.911	74.837	74.688	74.527	74.440	74.440	74.440
63	81.123	81.148	81.247	81.222	81.222	81.284	81.284	81.346	81.346	81.420	81.631	81.743	81.954	82.053	81.879	81.606	81.495	81.482	81.371	81.110	80.850	80.776	80.925	80.974	81.110	81.247	81.284	81.259	81.210
64	81.482	81.482	81.346	81.383	81.433	81.321	81.371	81.420	81.743	82.065	81.631	81.743	81.954	82.053	81.879	81.606	81.495	81.482	81.594	81.594	81.396	81.185	80.925	80.974	81.110	81.247	81.284	81.259	81.210
65	81.532	81.668	82.090	81.718	81.445	81.433	81.433	81.495	81.755	82.202	82.722	82.710	82.685	82.673	82.710	82.710	82.574	82.499	82.450	82.338	82.115	81.904	81.892	81.854	81.780	81.780	81.842	81.892	81.978
66	82.784	82.499	82.164	82.821	83.466	83.565	83.565	83.268	82.697	82.276	82.722	82.710	82.685	82.673	82.710	82.710	82.574	82.499	82.462	82.350	82.177	82.003	81.892	81.854	81.780	81.780	81.842	81.892	81.978
67	82.797	82.995	83.218	83.417	83.528	83.826	83.578	83.590	83.417	83.131	82.921	82.921	82.908	82.908	83.020	83.243	83.627	83.851	83.615	83.640	83.987	84.136	84.185	84.136	83.925	83.578	83.367	83.392	83.441
68	84.805	84.805	84.743	84.582	84.384	84.185	83.826	83.813	83.801	83.553	82.921	82.921	82.908	82.908	83.020	83.243	83.627	83.851	83.789	83.826	83.999	84.136	84.185	84.136	83.925	83.578	83.367	83.392	83.441
69	86.479	86.429	85.822	85.165	84.681	84.495	84.495	84.520	84.371	84.074	84.074	84.210	84.582	84.942	84.793	84.706	84.780	84.842	84.855	85.202	85.487	86.057	86.243	86.144	85.921	85.785	85.785	85.748	85.710
70	86.702	86.665	86.653	86.380	86.082	85.983	84.756	84.718	84.570	84.297	84.074	84.210	84.582	84.942	84.793	84.706	84.780	84.842	85.314	85.276	85.673	86.070	86.243	86.144	85.921	85.785	85.785	85.748	85.710
71	86.777	86.715	86.863	86.925	87.037	87.087	85.983	85.933	85.871	85.983	86.132	86.045	85.748	85.438	85.673	86.144	86.764	87.297	86.045	86.243	86.975	87.161	87.173	87.025	86.603	86.144	85.909	85.958	86.020
72	87.074	87.012	87.211	87.942	88.413	88.500	87.260	86.925	86.454	86.219	86.132	86.045	85.748	85.438	85.673	86.144	86.764	87.297	87.731	87.620	87.396	87.347	87.173	87.025	86.603	86.144	85.909	85.958	86.020
73	88.401	88.426	88.339	88.612	88.562	88.512	88.500	88.426	88.252	88.078	88.128	88.153	88.190	88.227	88.252	88.376	88.277	87.979	87.905	87.694	87.508	87.644	88.364	88.388	88.438	88.426	88.413	88.264	88.103
74	88.525	88.500	88.674	88.835	88.971	89.021	88.512	88.574	88.512	88.326	88.128	88.153	88.190	88.227	88.252	88.376	88.277	87.979	88.574	88.810	88.971	88.909	88.364	88.388	88.438	88.426	88.413	88.264	88.103
75	88.934	88.946	88.983	88.884	89.120	89.070	89.021	88.872	88.562	88.388	88.525	88.426	88.289	88.240	88.401	88.636	88.785	88.847	88.760	88.884	89.033	89.293	89.690	89.281	88.959	88.835	88.798	88.996	89.219
76	89.368	89.293	89.070	89.095	89.219	89.231	89.070	89.269	89.244	88.996	88.525	88.426	88.289	88.240	88.401	88.636	88.785	88.847	89.231	89.355	89.479	89.603	89.690	89.281	88.959	88.835	88.798	88.996	89.219
77	89.814	89.727	89.467	89.269	89.405	89.715	89.231	89.269	89.368	89.231	89.281	89.393	89.554	89.628	89.517	89.331	89.343	89.417	89.306	89.740	89.913	89.777	89.715	90.013	90.211	90.347	90.434	90.360	90.285
78	89.938	89.789	89.703	89.653	89.827	90.013	90.013	89.901	89.752	89.405	89.281	89.393	89.554	89.628	89.517	89.331	89.343	89.417	89.665	89.963	90.174	90.087	89.715	90.013	90.211	90.347	90.434	90.360	90.285
79	90.112	90.248	90.397	90.533	90.620	90.645	90.595	90.533	90.484	90.818	90.992	90.980	90.942	90.930	90.880	90.781	90.670	90.620	90.570	90.508	90.372	90.496	90.694	90.682	90.645	90.570	90.508	90.570	90.632
80	91.091	91.042	90.955	90.856	90.769	90.732	90.781	90.818	90.918	91.004	90.992	90.980	90.942	90.930	90.880	90.781	90.670	90.620	90.670	90.707	90.756	90.769	90.694	90.682	90.645	90.570	90.508	90.570	90.632
81	91.662	91.662	91.662	91.699	91.810	91.910	91.910	91.835	91.910	92.257	92.629	92.629	92.629	92.629	92.591	92.529	92.467	92.443	92.430	92.319	91.959	91.699	91.624	91.674	91.810	91.972	92.046	91.996	91.885
82	92.269	92.343	92.467	92.567	92.691	92.827	92.827	92.827	92.852	92.815	92.629	92.629	92.629	92.629	92.591	92.529	92.467	92.443	92.517	92.319	91.996	91.724	91.624	91.674	91.810	91.972	92.046	91.996	91.885
83	92.418	92.517	92.852	93.360	93.422	93.112	93.112	93.125	93.187	93.311	93.559	93.670	93.918	94.067	93.943	93.732	93.571	93.497	92.864	92.777	93.112	93.546	94.017	94.017	94.017	94.005	93.992	94.079	94.253
84	95.480	95.208	94.600	93.968	93.968	94.550	94.550	94.699	94.538	94.030	93.559	93.670	93.918	94.067	93.943	93.732	93.571	93.497	94.216	94.935	94.860	94.451	94.017	94.017	94.017	94.005	93.992	94.079	94.253
85	95.617	95.505	95.530	95.443	95.121	94.600	94.984	94.885	94.972	95.195	95.356	95.331	95.257	95.195	95.369	95.716	96.026	96.137	95.741	95.269	95.592	96.100	96.274	96.274	96.113	95.877	95.765	95.617	95.369
86	95.803	96.150	96.336	96.224	96.051	95.989	95.989	95.852	95.641	95.480	95.356	95.331	95.257	95.195	95.369	95.716	96.026	96.137	96.274	96.212	96.199	96.249	96.274	96.274	96.113	95.877	95.765	95.617	95.369
87	96.336	96.348	96.609	97.067	97.414	97.538	96.943	97.005	97.166	97.352	97.514	97.241	96.832	96.658	96.658	96.658	96.683	96.733	96.683	96.609	96.509	96.497	96.683	96.658	96.708	96.794	96.832	96.943	97.166
88	97.687	97.662	97.625	97.588	97.588	97.600	97.600	97.625	97.650	97.613	97.514	97.241	96.832	96.658	96.658	96.658	96.683	96.733	96.720	96.683	96.733	96.794	96.683	96.658	96.708	96.794	96.832	96.943	97.166
89	97.811	97.873	98.010	98.158	98.307	98.419	98.419	98.369	98.295	98.270	98.307	98.518	98.778	98.877	98.815	98.654	98.518	98.468	98.406	98.295	98.022	97.774	97.650	97.712	97.848	97.985	98.047	97.972	97.811
90	98.381	98.307	98.171	98.196	98.456	98.642	98.642	98.605	98.518	98.394	98.307	98.518	98.778	98.877	98.815	98.654	98.518	98.468	98.481	98.332	98.109	97.799	97.650	97.712	97.848	97.985	98.047	97.972	97.811

Table S4. List of modes in melamine calculated from the mode dispersion HLD simulation in CASTEP software. Mode numbers 1 – 90. For details, see the main article text.

Mode no.	$\omega(q_1)$	$\omega(q_2)$	$\omega(q_3)$	$\omega(q_4)$	$\omega(q_5)$	$\omega(q_6)$	$\omega(q_7)$	$\omega(q_8)$	$\omega(q_9)$	$\omega(q_{10})$	$\omega(q_{11})$	$\omega(q_{12})$	$\omega(q_{13})$	$\omega(q_{14})$
91	100.18	100.17	100.09	99.869	99.547	99.324	98.927	99.014	99.200	99.485	99.845	99.869	99.931	99.956
92	100.48	100.61	100.87	100.99	100.84	100.69	100.69	100.64	100.48	100.20	99.845	99.869	99.931	99.956
93	103.61	103.75	104.11	104.68	105.34	105.71	106.33	106.48	106.85	107.25	107.51	107.52	107.54	107.57
94	103.70	103.95	104.61	105.45	106.29	106.76	108.08	108.16	108.08	107.74	107.51	107.52	107.54	107.57
95	110.02	109.92	109.63	109.12	108.49	108.08	108.26	108.39	108.65	108.83	108.92	108.87	108.76	108.71
96	110.07	109.99	109.70	109.21	108.61	108.26	109.01	108.82	108.67	108.86	108.92	108.87	108.76	108.71
97	119.41	119.41	119.42	119.40	119.37	119.36	119.36	119.41	119.52	119.64	119.74	119.76	119.77	119.79
98	119.56	119.48	119.43	119.47	119.55	119.60	119.62	119.63	119.69	119.78	119.74	119.76	119.77	119.79
99	119.60	119.69	119.78	119.76	119.71	119.67	119.67	119.69	119.76	119.79	119.92	119.92	119.91	119.89
100	119.78	119.78	119.81	119.89	119.94	119.95	119.94	119.99	120.04	120.02	119.92	119.92	119.91	119.89
101	126.59	126.59	126.71	126.71	126.84	126.84	127.08	127.08	126.96	126.84	126.71	126.71	126.84	126.84
102	126.71	126.71	126.84	126.96	127.21	127.33	127.21	127.08	126.96	126.84	126.71	126.71	126.84	126.84
103	127.58	127.58	127.70	127.70	127.58	127.46	127.33	127.46	127.70	127.83	127.83	127.83	127.70	127.70
104	127.83	127.83	127.83	127.70	127.58	127.58	127.46	127.70	127.83	127.83	127.83	127.83	127.70	127.70
105	127.95	127.95	127.83	127.83	127.83	127.95	127.83	127.83	128.08	128.32	128.45	128.45	128.57	128.57
106	128.45	128.32	128.08	128.08	128.08	128.20	128.20	128.20	128.45	128.45	128.45	128.45	128.57	128.57
107	128.57	128.70	128.82	128.70	128.57	128.45	128.45	128.45	128.45	128.82	129.07	129.07	129.07	129.19
108	128.94	128.94	128.82	128.82	128.82	128.82	129.19	129.19	129.19	129.19	129.07	129.07	129.07	129.19
109	143.57	143.57	143.70	143.82	143.82	143.82	143.82	143.70	143.33	143.08	142.95	142.95	143.08	143.08
110	143.82	143.82	143.82	143.82	143.95	143.95	143.95	143.82	143.57	143.20	142.95	142.95	143.08	143.08
111	144.81	144.81	144.81	144.81	144.81	144.81	144.81	144.57	144.32	144.19	144.19	144.19	144.19	144.19
112	144.94	144.94	144.94	144.94	144.94	144.94	144.94	144.94	144.69	144.44	144.19	144.19	144.19	144.19
113	145.43	145.31	145.31	145.31	145.19	145.19	145.31	145.43	145.81	146.05	146.05	146.05	146.05	146.05
114	145.68	145.68	145.68	145.68	145.81	145.81	145.81	146.05	146.18	146.05	146.05	146.05	146.05	146.05
115	146.80	146.67	146.67	146.55	146.43	146.30	146.18	146.18	146.43	146.80	147.29	147.29	147.29	147.29
116	147.42	147.54	147.54	147.54	147.54	147.54	147.79	147.67	147.42	147.67	147.29	147.29	147.29	147.29
117	147.79	147.79	147.91	148.04	148.04	148.16	148.16	148.16	148.04	147.79	148.16	148.16	148.29	148.29
118	148.16	148.16	148.16	148.16	148.16	148.16	148.16	148.29	148.53	148.78	148.16	148.16	148.29	148.29
119	149.65	149.65	149.65	149.65	149.65	149.65	149.65	149.52	149.15	148.78	148.91	148.91	148.91	148.91
120	149.65	149.65	149.77	149.90	149.90	149.90	149.77	149.65	149.40	149.15	148.91	148.91	148.91	148.91
121	163.41	163.41	163.41	163.41	163.41	163.41	163.41	163.41	163.41	163.41	163.41	163.41	163.41	163.41
122	163.41	163.41	163.41	163.41	163.41	163.41	163.41	163.41	163.41	163.41	163.41	163.41	163.41	163.41
123	163.41	163.54	163.54	163.66	163.66	163.66	163.66	163.66	163.54	163.54	163.54	163.41	163.41	163.41
124	163.54	163.54	163.66	163.66	163.66	163.78	163.78	163.66	163.66	163.54	163.54	163.41	163.41	163.41
125	176.55	176.55	176.68	176.93	177.05	177.17	177.30	177.42	177.42	177.42	177.30	177.42	177.42	177.55
126	176.68	176.68	176.80	176.93	177.17	177.30	177.55	177.55	177.67	177.42	177.30	177.42	177.42	177.55
127	178.29	178.17	178.04	177.79	177.55	177.55	177.79	177.79	178.04	178.66	178.91	178.79	178.54	178.41
128	178.29	178.29	178.17	178.04	177.92	177.79	178.79	178.79	178.66	178.91	178.91	178.79	178.54	178.41
129	180.40	180.40	180.40	180.40	180.40	180.27	180.27	180.15	180.03	180.15	180.52	180.77	181.26	181.64
130	180.65	180.65	180.52	180.40	180.40	180.40	180.40	180.65	181.02	180.89	180.52	180.77	181.26	181.64
131	181.64	181.76	181.64	181.51	181.39	181.39	181.02	181.51	181.76	182.63	183.25	183.12	182.75	182.38
132	182.01	181.88	181.88	182.01	182.01	182.01	181.51	181.51	182.38	183.12	183.25	183.12	182.75	182.38
133	184.36	184.49	184.74	185.11	185.60	186.10	186.10	185.73	184.98	184.86	185.11	185.23	185.60	185.73
134	187.46	187.46	187.59	187.59	186.97	186.35	186.35	185.98	185.85	185.48	185.11	185.23	185.60	185.73
135	188.08	188.08	187.96	187.59	187.34	187.34	187.34	187.59	187.46	187.22	187.46	187.34	187.22	187.22
136	188.58	188.46	188.08	187.84	187.96	187.96	188.21	188.08	188.08	187.84	187.46	187.34	187.22	187.22
137	188.83	188.83	188.83	188.58	188.58	188.46	189.08	189.20	189.45	189.45	189.57	189.57	189.45	189.45
138	188.83	188.95	188.95	188.95	189.08	189.08	189.70	189.82	189.94	189.70	189.57	189.57	189.45	189.45
139	189.08	188.95	189.45	189.94	190.56	190.69	190.69	190.56	190.44	190.32	190.56	190.56	190.56	190.56
140	190.19	190.19	190.32	190.44	190.56	190.81	190.81	190.81	190.69	190.81	190.56	190.56	190.56	190.56
141	190.19	190.32	190.44	190.56	190.81	190.81	190.81	190.81	190.94	191.43	191.93	191.93	192.05	192.05
142	191.56	191.68	191.80	191.80	191.68	191.68	191.06	191.31	192.18	192.18	191.93	191.93	192.05	192.05
143	194.16	194.16	194.16	193.79	193.04	192.67	191.68	191.93	192.18	192.92	193.54	193.54	193.54	193.54
144	195.40	195.28	194.66	194.16	194.16	194.16	195.15	195.03	194.66	194.16	193.54	193.54	193.54	193.54
145	200.61	200.61	200.61	200.48	200.48	200.48	200.48	200.61	201.10	201.60	201.85	201.85	201.85	201.85
146	200.73	200.73	200.61	200.61	200.61	200.61	201.85	201.85	201.85	201.85	201.85	201.85	201.85	201.85
147	201.23	201.23	201.47	201.60	201.85	201.85	201.97	201.97	201.97	202.09	202.22	202.22	202.09	202.09
148	201.72	201.72	201.72	201.85	201.97	201.97	202.96	202.84	202.71	202.47	202.22	202.22	202.09	202.09
149	203.58	203.58	203.71	203.83	204.08	204.20	203.58	203.58	203.71	203.95	204.33	204.45	204.57	204.82
150	203.58	203.71	203.95	204.45	204.95	205.19	204.20	204.45	204.95	204.82	204.33	204.45	204.57	204.82
151	205.81	205.69	205.69	205.44	205.32	205.32	205.44	205.44	205.19	205.44	205.57	205.44	205.19	204.95
152	206.19	206.19	206.06	205.81	205.69	205.44	206.06	205.81	205.69	205.44	205.57	205.44	205.19	204.95
153	206.43	206.43	206.43	206.31	206.31	206.31	206.31	206.31	206.19	206.06	205.94	206.06	206.31	206.43
154	207.30	207.30	207.30	207.18	206.93	206.93	206.93	206.81	206.68	206.19	205.94	206.06	206.31	206.43
155	207.43	207.43	207.43	207.30	207.18	207.18	207.18	207.05	206.93	206.81	206.68	206.68	206.56	206.56
156	208.05	207.92	207.80	207.67	207.67	207.67	207.80	207.55	207.18	206.81	206.68	206.68	206.56	206.56
157	383.36	383.36	383.36	383.36	383.24	383.24	383.24	383.24	383.36	383.36	383.36	383.36	383.36	383.36
158	383.48	383.48	383.48	383.48	383.48	383.48	383.48	383.48	383.48	383.36	383.36	383.36	383.36	383.36
159	387.20	387.20	387.08	386.95	386.83	386.71	385.71	385.84	386.09	386.46	386.83	386.83	387.08	387.08
160	388.57	388.57	388.57	388.69	388.81	388.81	387.70	387.70	387.45	387.08	386.83	386.83	387.08	387.08
161	390.92	390.92	390.80	390.80	390.80	390.80	390.80	390.80	390.80	390.80	390.92	390.92	390.92	390.92
162	391.05	390.92	391.05	391.05	390.92	390.92	390.92	390.92	390.92	390.92	390.92	390.92	390.92	390.92
163	392.29	392.29	392.29	392.29	392.41	392.41	392.16	392.16	392.29	392.66	393.28	393.28	393.28	393.28
164	393.28	393.28	393.40	393.53	393.53	393.53	393.90	393.90	394.15	394.02	393.28	393.28	393.28	393.28
165	394.02	394.02	393.90	393.90	393.90	393.90	394.52	394.64	394.64	394.64	395.51	395.51	395.51	395.63
166	394.64	394.77	395.01	395.26	395.63	395.76	396.13	395.76	395.39	395.76	395.51	395.51	395.51	395.63
167	399.97	399.85	399.60	399.11	398.86	398.61	398.61	398.11	397.25	396.38	396.63	396.63	396.63	396.50
168	400.22	400.35	400.47	400.72	400.84	400.84	399.48	399.35	398.73	397.74	396.63	396.63	396.63	396.50
169	412.62	412.62	412.62	412.62	412.74	412.74	412.74	412.87	413.36	413.86	414.11	414.11	414.23	414.23
170	412.74	412.74	412.74	412.74	412.74	412.74	413.12	413.36	413.74	413.98	414.11	414.11	414.23	414.23
171	413.12	413.24	413.24	413.24	413.24	413.24	413.24	413.49	414.11	414.60	414.85	414.85	414.85	414.85
172	413.24	413.24	413.24	413.24	413.24	413.36	416.09	415.97	415.47	415.10	414.85	414.85	414.85	414.85
173	433.82	433.82	433.82	433.82	433.70	433.70	433.70	433.70	433.70	433.70	433.70	433.70	433.70	433.70
174	433.94	433.82	433.82	433.82	433.82	433.82	433.82	433.82	433.82	433.70	433.70	433.70	433.70	433.70
175	434.19	434.19	434.19	434.19	434.07	434.07	433.94	433.94	433.94	433.94	433.94	433.94	433.94	433.94
176	434.56	434.44	434.44	434.32	434.19	434.19	434.07	434.07	434.07	433.94	433.94	433.94	433.94	433.94
177	439.65	439.65	439.65	439.65	439.65	439.65	439.65	439.65	439.77	439.90	439.90	439.90	440.02	440.02
178	439.65	439.65	439.77	439.77	439.77	439.77	439.77	439.77	439.77	439.90	439.90	439.90	440.02	440.02
179	439.77	439.77	439.77	439.77	439.77	439.77	439.77	439.90	440.02	440.14	440.14	440.14	440.27	440.27
180	439.77	439.77	439.77	439.77	439.77	439.77	440.27	440.27	440.14	440.14	440.14	440.14	440.27	440.27

Mode no.	$\omega(q_{15})$	$\omega(q_{16})$	$\omega(q_{17})$	$\omega(q_{18})$	$\omega(q_{19})$	$\omega(q_{20})$	$\omega(q_{21})$	$\omega(q_{22})$	$\omega(q_{23})$	$\omega(q_{24})$	$\omega(q_{25})$	$\omega(q_{26})$	$\omega(q_{27})$	$\omega(q_{28})$	$\omega(q_{29})$
91	100.01	100.10	100.17	100.19	100.08	100.12	100.34	100.66	100.94	100.87	100.69	100.49	100.41	100.45	100.51
92	100.01	100.10	100.17	100.19	100.46	100.79	101.02	101.08	100.94	100.87	100.69	100.49	100.41	100.45	100.51
93	107.44	107.04	106.37	105.93	105.39	104.61	103.97	103.69	103.75	103.89	104.27	104.70	104.89	104.89	104.89
94	107.44	107.04	106.37	105.93	105.82	105.23	104.56	104.05	103.75	103.89	104.27	104.70	104.89	104.89	104.89
95	108.65	108.47	108.26	108.15	108.26	108.82	109.44	109.95	110.21	110.36	110.67	110.87	110.93	110.99	111.09
96	108.65	108.47	108.26	108.15	108.61	109.25	109.81	110.16	110.21	110.36	110.67	110.87	110.93	110.99	111.09
97	119.72	119.60	119.48	119.45	119.42	119.38	119.38	119.41	119.48	119.48	119.50	119.52	119.52	119.53	119.55
98	119.72	119.60	119.48	119.45	119.50	119.56	119.60	119.56	119.48	119.48	119.50	119.52	119.52	119.53	119.55
99	119.92	119.91	119.87	119.84	119.79	119.73	119.69	119.69	119.73	119.76	119.84	119.93	119.95	119.95	119.95
100	119.92	119.91	119.87	119.84	119.88	119.88	119.84	119.78	119.73	119.76	119.84	119.93	119.95	119.95	119.95
101	126.84	126.96	126.96	127.08	126.84	126.71	126.59	126.59	126.71	126.71	126.84	127.08	127.33	127.33	127.33
102	126.84	126.96	126.96	127.08	127.08	126.96	126.84	126.71	126.71	126.71	126.84	127.08	127.33	127.33	127.33
103	127.70	127.70	127.70	127.58	127.58	127.70	127.70	127.70	127.83	127.70	127.70	127.70	127.58	127.58	127.58
104	127.70	127.70	127.70	127.58	127.70	127.70	127.70	127.83	127.83	127.70	127.70	127.70	127.58	127.58	127.58
105	128.45	128.08	127.95	128.08	127.95	127.95	127.95	128.08	128.08	128.20	128.45	128.70	128.82	128.70	128.70
106	128.45	128.08	127.95	128.08	128.08	128.20	128.20	128.08	128.08	128.20	128.45	128.70	128.82	128.70	128.70
107	129.07	128.94	128.70	128.57	128.45	128.45	128.57	128.70	128.82	128.94	128.94	128.94	128.94	128.94	129.07
108	129.07	128.94	128.70	128.57	128.82	128.94	129.07	128.94	128.82	128.94	128.94	128.94	128.94	128.94	129.07
109	143.20	143.45	143.82	143.95	143.82	143.70	143.57	143.57	143.57	143.57	143.57	143.45	143.45	143.45	143.45
110	143.20	143.45	143.82	143.95	144.07	143.95	143.82	143.70	143.57	143.57	143.57	143.45	143.45	143.45	143.45
111	144.32	144.57	144.81	144.94	144.81	144.81	144.81	144.81	144.81	144.81	144.57	144.44	144.44	144.44	144.32
112	144.32	144.57	144.81	144.94	144.94	144.94	144.94	144.81	144.81	144.81	144.57	144.44	144.44	144.44	144.32
113	146.05	145.93	145.56	145.43	145.43	145.43	145.43	145.43	145.56	145.68	145.93	146.30	146.43	146.43	146.43
114	146.05	145.93	145.56	145.43	145.43	145.43	145.56	145.56	145.56	145.68	145.93	146.30	146.43	146.43	146.43
115	147.17	146.92	147.05	147.05	146.80	146.80	146.80	146.92	147.05	147.17	147.42	147.67	147.79	147.79	147.79
116	147.17	146.92	147.05	147.05	147.29	147.29	147.29	147.17	147.05	147.17	147.42	147.67	147.79	147.79	147.79
117	148.29	148.29	148.29	148.29	148.29	148.29	148.16	148.04	148.16	148.04	148.04	148.16	148.29	148.29	148.29
118	148.29	148.29	148.29	148.29	148.29	148.29	148.29	148.16	148.16	148.04	148.04	148.16	148.29	148.29	148.29
119	149.03	149.40	149.65	149.65	149.65	149.65	149.65	149.65	149.65	149.52	149.28	148.91	148.78	148.78	148.78
120	149.03	149.40	149.65	149.65	149.65	149.77	149.77	149.65	149.65	149.52	149.28	148.91	148.78	148.78	148.78
121	163.41	163.41	163.41	163.41	163.41	163.41	163.41	163.41	163.41	163.41	163.41	163.54	163.54	163.54	163.54
122	163.41	163.41	163.41	163.41	163.41	163.41	163.41	163.41	163.41	163.41	163.41	163.54	163.54	163.54	163.54
123	163.54	163.66	163.78	163.78	163.78	163.66	163.54	163.54	163.54	163.54	163.66	163.66	163.66	163.66	163.78
124	163.54	163.66	163.78	163.78	163.78	163.66	163.66	163.54	163.54	163.54	163.66	163.66	163.66	163.66	163.78
125	177.55	177.55	177.42	177.30	177.05	176.68	176.55	176.43	176.55	176.68	177.17	177.67	178.17	178.04	178.04
126	177.55	177.55	177.42	177.30	177.42	177.17	176.80	176.68	176.55	176.68	177.17	177.67	178.17	178.04	178.04
127	178.41	178.29	177.79	177.67	177.55	177.67	178.04	178.29	178.41	178.54	178.66	178.54	178.17	178.29	178.41
128	178.41	178.29	177.79	177.67	177.92	178.17	178.29	178.41	178.41	178.54	178.66	178.54	178.17	178.29	178.41
129	181.39	180.89	180.52	180.40	180.40	180.40	180.27	180.40	180.52	180.65	180.77	180.89	181.02	180.89	180.77
130	181.39	180.89	180.52	180.40	180.52	180.77	180.77	180.77	180.52	180.65	180.77	180.89	181.02	180.89	180.77
131	182.26	181.88	181.51	181.51	181.39	181.51	181.51	181.64	181.88	181.88	181.88	182.01	182.26	182.13	181.88
132	182.26	181.88	181.51	181.51	181.64	181.51	181.88	181.88	181.88	181.88	181.88	182.01	182.26	182.13	181.88
133	185.48	185.48	185.98	186.60	186.60	186.35	186.10	185.98	185.98	185.98	186.22	186.47	186.84	186.84	186.97
134	185.48	185.48	185.98	186.60	186.60	186.47	186.22	186.10	185.98	185.98	186.22	186.47	186.84	186.84	186.97
135	187.71	188.33	188.08	187.46	187.09	187.34	187.96	188.46	188.58	188.58	188.33	187.96	187.59	187.59	187.46
136	187.71	188.33	188.08	187.46	187.71	187.96	188.21	188.46	188.58	188.58	188.33	187.96	187.59	187.59	187.46
137	189.20	188.70	188.70	188.70	188.70	188.70	188.83	188.95	188.95	188.83	188.83	188.83	188.95	188.83	188.70
138	189.20	188.70	188.70	188.70	188.83	188.95	189.08	188.95	188.95	188.83	188.83	188.83	188.95	188.83	188.70
139	190.56	190.69	190.44	190.19	189.45	189.20	189.08	189.08	189.32	189.45	189.45	189.20	189.08	189.32	189.57
140	190.56	190.69	190.44	190.19	190.69	190.44	190.07	189.70	189.32	189.45	189.45	189.20	189.08	189.32	189.57
141	191.80	191.31	190.94	190.81	190.81	190.44	190.32	190.32	190.44	190.56	190.94	191.06	191.18	191.18	191.31
142	191.80	191.31	190.94	190.81	191.06	190.94	190.81	190.56	190.44	190.56	190.94	191.06	191.18	191.18	191.31
143	193.42	193.17	192.67	192.42	192.92	194.04	194.66	195.28	195.65	195.40	195.03	194.90	194.90	194.78	194.66
144	193.42	193.17	192.67	192.42	193.17	194.53	195.52	195.77	195.65	195.40	195.03	194.90	194.90	194.78	194.66
145	201.60	201.23	200.73	200.48	200.48	200.48	200.61	200.61	200.73	200.98	201.35	201.35	201.35	201.35	201.35
146	201.60	201.23	200.73	200.48	200.61	200.61	200.73	200.73	200.73	200.98	201.35	201.35	201.35	201.35	201.35
147	201.97	201.97	201.97	201.97	201.85	201.72	201.72	201.60	201.72	201.72	201.97	202.71	203.09	203.09	203.09
148	201.97	201.97	201.97	201.97	202.09	201.97	201.72	201.72	201.72	201.72	201.97	202.71	203.09	203.09	203.09
149	204.70	204.33	204.08	203.95	203.46	203.46	203.71	203.83	203.83	203.83	203.95	204.08	204.45	204.33	204.33
150	204.70	204.33	204.08	203.95	204.33	204.20	203.95	203.83	203.83	203.83	203.95	204.08	204.45	204.33	204.33
151	205.07	205.32	205.57	205.57	205.57	205.69	205.81	205.94	205.94	205.81	205.57	205.07	204.45	204.57	204.70
152	205.07	205.32	205.57	205.57	205.81	205.81	205.81	205.94	205.94	205.81	205.57	205.07	204.45	204.57	204.70
153	206.31	206.31	206.31	206.31	206.43	206.56	206.68	206.81	206.93	206.81	206.56	206.43	206.43	206.43	206.31
154	206.31	206.31	206.31	206.31	206.68	207.18	207.18	207.05	206.93	206.81	206.56	206.43	206.43	206.43	206.31
155	206.81	207.05	207.30	207.43	207.43	207.30	207.55	207.80	207.92	207.80	207.55	207.18	207.05	207.05	207.05
156	206.81	207.05	207.30	207.43	207.55	207.67	207.80	207.92	207.92	207.80	207.55	207.18	207.05	207.05	207.05
157	383.36	383.36	383.36	383.36	383.36	383.36	383.36	383.36	383.36	383.36	383.36	383.36	383.36	383.36	383.36
158	383.36	383.36	383.36	383.36	383.36	383.36	383.36	383.36	383.36	383.36	383.36	383.36	383.36	383.36	383.36
159	386.95	386.71	386.58	386.46	385.84	385.96	386.58	387.33	388.19	387.82	387.08	386.46	386.21	386.21	386.21
160	386.95	386.71	386.58	386.46	387.57	388.44	388.81	388.69	388.19	387.82	387.08	386.46	386.21	386.21	386.21
161	390.92	390.80	390.80	390.80	390.80	390.80	390.80	390.92	390.92	390.92	390.80	390.80	390.67	390.67	390.80
162	390.92	390.80	390.80	390.80	390.80	390.80	390.92	390.92	390.92	390.92	390.80	390.80	390.67	390.67	390.80
163	393.40	393.65	393.65	393.53	393.28	393.15	393.15	393.15	393.15	393.40	393.40	393.15	393.03	393.15	393.15
164	393.40	393.65	393.65	393.53	393.65	393.53	393.28	393.28	393.15	393.40	393.40	393.15	393.03	393.15	393.15
165	395.01	394.39	394.39	394.52	394.27	394.27	394.27	394.27	394.27	394.27	394.27	394.77	395.14	395.14	395.26
166	395.01	394.39	394.39	394.52	394.64	394.52	394.39	394.27	394.27	394.27	394.27	394.77	395.14	395.14	395.26
167	397.25	398.11	398.73	398.98	399.11	399.73	400.10	400.22	400.35	399.85	398.86	397.62	397.00	397.00	396.87
168	397.25	398.11	398.73	398.98	399.35	399.85	400.22	400.35	400.35	399.85	398.86	397.62	397.00	397.00	396.87
169	414.11	413.61	412.99	412.74	412.74	412.74	412.74	412.74	412.74	412.87	413.36	413.74	413.86	413.86	413.86
170	414.11	413.61	412.99	412.74	412.74	412.74	412.74	412.74	412.74	412.87	413.36	413.74	413.86	413.86	413.86
171	414.73	414.23	413.49	413.24	413.12	413.12	413.12	413.24	413.24	413.49	413.98	414.36	414.48	414.60	414.60
172	414.73	414.23	413.49	413.24	413.36	413.36	413.36	413.36	413.24	413.49	413.98	414.36	414.48	414.60	414.60
173	433.70	433.70	433.70	433.70	433.70	433.70	433.82	433.82	433.94	433.82	433.70	433.70	433.57	433.57	433.57
174	433.70	433.70	433.70	433.70	433.70	433.82	433.82	433.94	433.94	433.82	433.70	433.70	433.57	433.57	433.57
175	433.94	433.94	433.94	433.94	433.94	434.07	434.19	434.32	434.44	434.32	434.19	434.07	433.94	433.94	433.94
176	433.94	433.94	433.94	433.94	434.07	434.32	434.44	434.44	434.44	434.32	434.19	434.07	433.94	433.94	433.94
177	439.90	439.77	439.65	439.65	439.65	439.65	439.65	439.65	439.65	439.65	439.65	439.65	439.65	439.65	439.65
178	439.90	439.77	439.65	439.65	439.65	439.65	439.65	439.65	439.65	439.65	439.65	439.65	439.65	439.65	439.65
179	440.14	440.02	439.90	439.77	439.77	439.77	439.77	439.77	439.77	439.77	439.77	439.90	439.90	439.90	439.90
180	440.14	440.02	439.90	439.77	439.77	439.77	439.77	439.77	439.77	439.77	439.77	439.90	439.90	439.90	439.90

Table S5. List of modes in melamine calculated from the mode dispersion HLD simulation in CASTEP software. Mode numbers 91 – 180. For details, please take a look at the main article text.

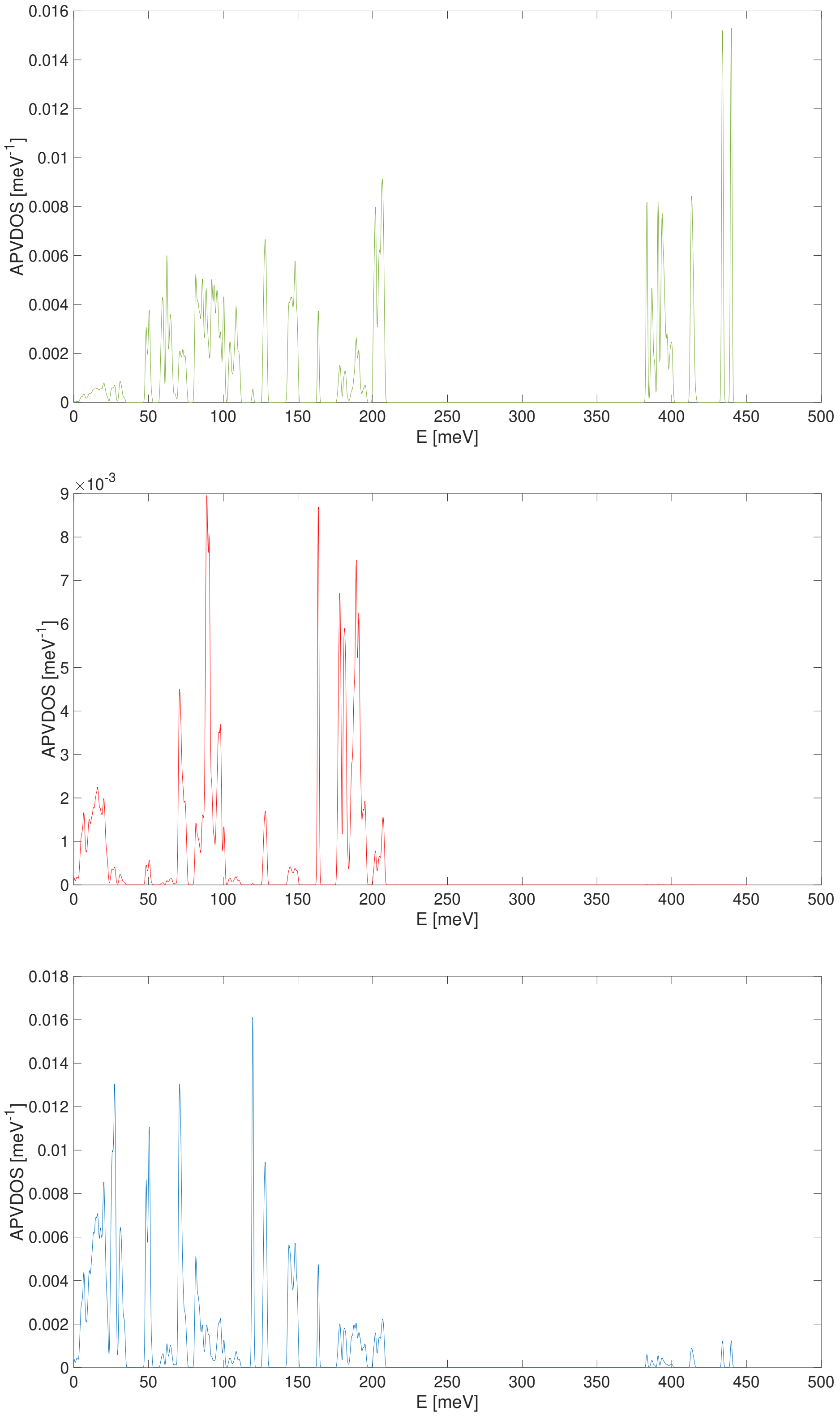

Figure S17. apVDoSs calculated from the mode dispersion HLD simulation in CASTEP software. apVDoS for hydrogen (top panel), carbon (middle panel), and nitrogen (bottom panel). For details, see the text of the main article.

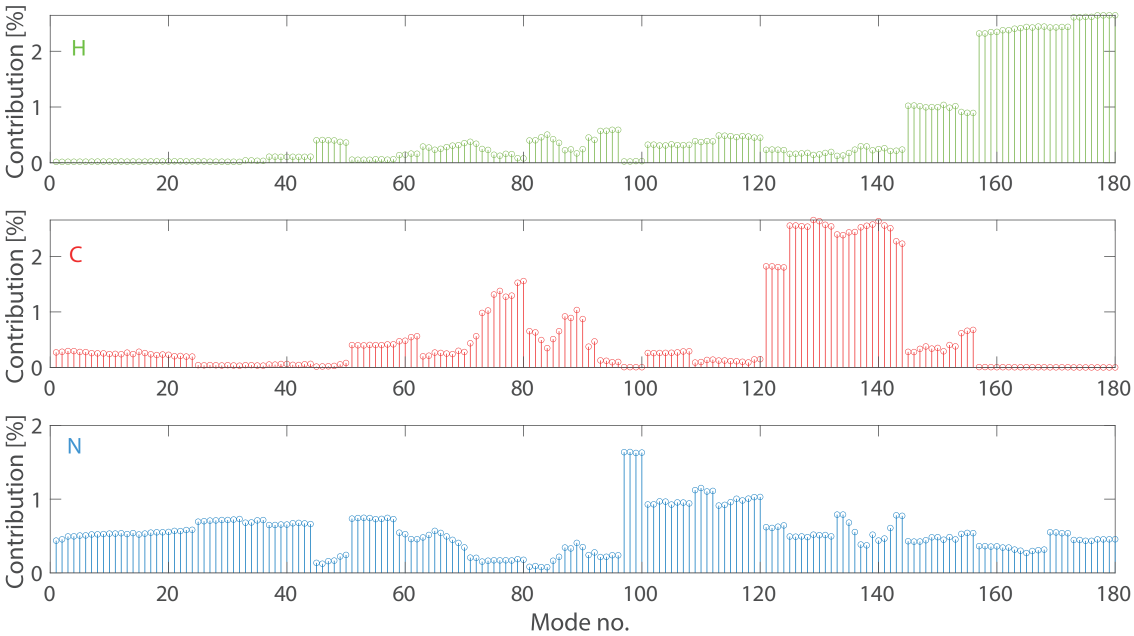

Figure S18. q-averaged individual mode contributions to the total kinetic energy of protons (top panel), carbon (middle panel), and nitrogen (bottom panel) in melamine. For details, see the text of the main article.

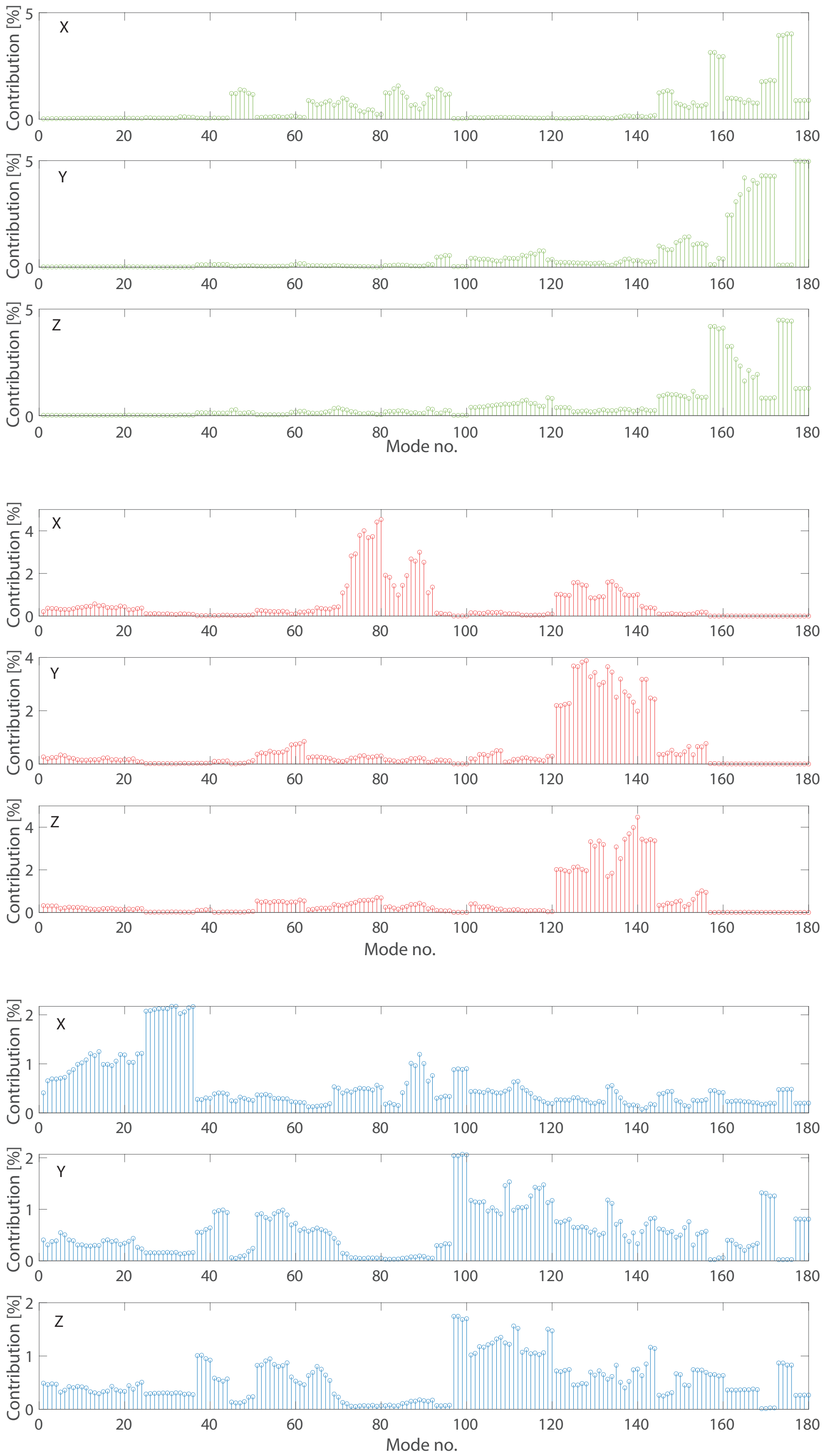

Figure S19. q-averaged individual mode contributions to the total kinetic energy of protons (top panel), carbon (middle panel), and nitrogen (bottom panel) in melamine along the X, Y, and Z crystallographic axes. For details, see the text of the main article.
